# Molecular basis of polyadenylated RNA fate determination in the nucleus

**DOI:** 10.1038/s41586-026-10650-0

**Published:** 2026-06-17

**Authors:** Andrii Bugai, Ulrich Hohmann, Ana Lorenzo, Max Graf, Laura Fin, Jérôme O. Rouvière, Laszlo Tirian, Yuhui Dou, Marion Le Rest, Patrik Polák, Dennis Johnsen, Lis Jakobsen, Jens Skorstengaard Andersen, Julius Brennecke, Clemens Plaschka, Torben Heick Jensen

**Affiliations:** 1 Department of Molecular Biology and Genetics, Aarhus University, Aarhus, Denmark; 2 Research Institute of Molecular Pathology (IMP), Vienna BioCenter (VBC), Vienna, Austria; 3 Institute of Molecular Biotechnology of the Austrian Academy of Sciences (IMBA), Vienna BioCenter (VBC), Vienna, Austria; 4 Vienna BioCenter PhD Program, Doctoral School of the University of Vienna and Medical University of Vienna, Vienna, Austria; 5 Friedrich Miescher Institute for Biomedical Research, Basel, Switzerland; 6 Department of Biochemistry and Molecular Biology, University of Southern Denmark, Odense M, Denmark; 7 Present Address: Institute of Molecular Biology, Mainz, Germany

**Keywords:** RNA quality control, RNA transport

## Abstract

Eukaryotic genomes generate a plethora of polyadenylated (pA^+^) RNAs^1,2^, which are packaged into ribonucleoprotein particles (RNPs). To ensure faithful gene expression, functional pA^+^ RNPs, including protein-coding RNPs, are exported to the cytoplasm, whereas transcripts within non-functional pA^+^ RNPs are degraded in the nucleus^1–4^. How cells distinguish these opposing fates remains unknown. The DExD-box ATPase UAP56 (also known as DDX39B) is a central component of functional pA^+^ RNPs, and promotes their docking to the nuclear pore complex-anchored TREX-2^5,6^, which triggers transcript release from UAP56 to facilitate export^7^. Here we reveal that the poly(A) tail exosome targeting (PAXT) connection^8^ binds a TREX-2-like module, which releases pA^+^ RNAs from UAP56 for decay by the nuclear exosome. The core of this module consists of a LENG8–PCID2–SEM1 trimer, which we show is structurally and biochemically equivalent to the central GANP–PCID2–SEM1 trimer of TREX-2. Mutagenesis and transcriptomic data demonstrate that the nuclear fate of pA^+^ RNPs is governed by the contending actions of nucleoplasmic PAXT and nuclear pore complex-associated TREX-2, which interpret RNA-bound UAP56 as a signal for RNA decay or export, respectively. As RNA targets of PAXT are generally short and intron-poor, we propose an overall model for pA^+^ RNP fate determination whereby the distinct sub-nuclear localizations of PAXT and TREX-2 govern the degradation of short non-functional pA^+^ RNAs while allowing export of their longer and functional counterparts.

## Abstract (web summary)

Biochemical, structural and cell biological analyses reveal that UAP56 (DDX39B) assembles with a TREX-2–like module that redirects non-functional polyadenylated RNAs from export to degradation.

## Main

RNA polymerase II (RNAPII) extensively transcribes mammalian genomes, yielding a wide range of unadenylated and polyadenylated RNAs^1–4^. Moreover, individual transcription units that generate standard full-length transcripts also give rise to an array of shorter isoforms^9,10^. Thus, functional RNAs are produced alongside a wealth of futile RNAPII products. Whereas mature functional pA^+^ RNAs, such as protein-coding mRNAs, are exported from the nucleus to the cytoplasm, their non-functional counterparts are typically retained and degraded^3,4^. This is primarily achieved by the nucleoplasmic PAXT connection, which consists of a heterodimeric core of the RNA helicase MTR4 and the Zn-finger protein ZFC3H1^8,11^. Additional, and less well-described, interactions with the nuclear pA^+^ RNA-binding protein PABPN1 and other transiently interacting RNP components, sometimes referred to as extended PAXT components, may aid in directing transcript turnover by the 3′−5′ exonucleolytic exosome complex^12–15^. However, how these interactions provide a biochemical basis by which PAXT distinguishes non-functional pA^+^ RNAs remains a major unresolved question.

Prior to their nuclear export, pA^+^ RNAs are packaged with proteins into pA^+^ RNPs. Central to this process is the export factor and DExD-box ATPase UAP56, which is recruited to pA^+^ RNPs in preparation for their nuclear export^5,16^. At the nuclear envelope, the activity of the nuclear pore complex (NPC)-associated GANP–PCID2–SEM1 (GANP–PS) trimer of TREX-2^6^ facilitates the release of RNA from UAP56, enabling export^7,17^. Again, how pA^+^ RNP sorting is orchestrated to favour the selected export of functional pA^+^ RNAs is unknown.

Here we interrogate two TREX-2-like human complexes, SAC3D1–PCID2–SEM1 (SAC3D1–PS) and LENG8–PCID2–SEM1 (LENG8–PS), in which the conserved SAC3D1 and LENG8 proteins, respectively, replace GANP. The GANP–PS, SAC3D1–PS and LENG8–PS complexes are structurally similar and share the ability to release UAP56 from RNA. Notably, we show that LENG8–PS offers PAXT a module, that acts on UAP56 to promote transcript turnover in contrast to the RNA export activity of TREX-2. Our findings reveal that nuclear pA^+^ RNA export and decay utilize a shared biochemical mechanism to act on pA^+^ RNPs but with fundamentally different outcomes. Based on the substrate preference of PAXT and its separate nuclear localization from TREX-2, we propose a general model for pA^+^ RNP fate determination.

## TREX-2-like complexes release RNA from UAP56

**Fig. 1:**
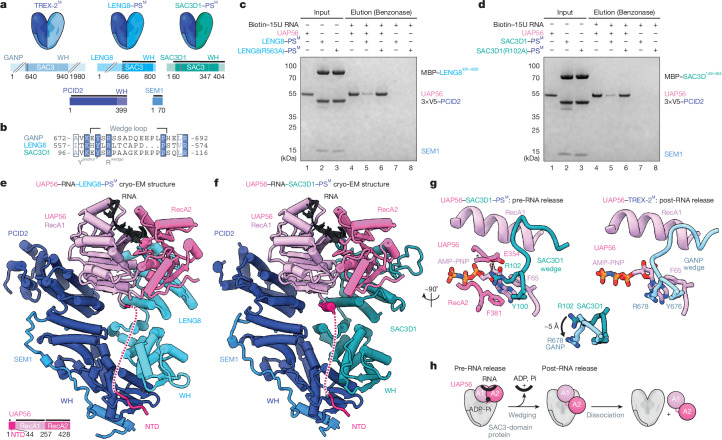
TREX-2-like complexes bind UAP56 to trigger RNA release. **a**, Cartoons of TREX-2 and TREX-2-like core complexes (top) and their domain architectures (bottom). PCID2, dark blue; SEM1, mid blue; SAC3 domain-containing proteins GANP, LENG8 and SAC3D1, shades of blue. Wedge loop positions are shown as grey bars. Regions included in the atomic models in **e**,**f** are indicated by black lines. WH, winged helix. **b**, Multiple sequence alignment of wedge loop sequences of human GANP (UniProt O60318), LENG8 (UniProt Q96PV6) and SAC3D1 (UniProt A6NKF1), with a conserved tyrosine residue anchoring the wedge loop on the SAC3 domain (Y^anchor^) and the central wedge loop arginine (R^wedge^) highlighted. Colouring by conservation (blue letters, conserved residue; blue background, invariant residue). **c**,**d**, UAP56 RNA release assay, demonstrating the stimulatory effects of LENG8–PS^M^ (**c**) or SAC3D1–PS^M^ (**d**) complexes. Bead-immobilized 15 poly-uridine RNA was incubated with UAP56 and ATP to form UAP56–ADP-P_i_–RNA complexes^7^ and subsequently challenged with recombinant LENG8–PS^M^ or SAC3D1–PS^M^ complexes, or their respective wedge loop mutants. Remaining UAP56–ADP-P_i_-RNA complexes were analysed by SDS–PAGE and Coomassie staining. **e**,**f**, Cartoon representation of cryo-EM structures of UAP56–RNA–LENG8–PS^M^ (**e**) or UAP56–RNA–SAC3D1–PS^M^ (**f**) complexes at resolutions ranging from 6 to 12 Å for UAP56–RNA–LENG8–PS^M^ and 2.6 to 4.5 Å for UAP56–RNA–SAC3D1–PS^M^. SEM1, blue; PCID2, dark blue; LENG8^491–800^, light blue; SAC3D1^48–404^, green blue; UAP56, shades of pink; flexible region of UAP56 N-terminal domain, dashed line; RNA, black. **e**, Bottom left, UAP56 domain structure. **g**, Details of the SAC3D1 wedge loop–UAP56–nucleotide interactions for UAP56–RNA–SAC3D1–PS^M^ (left) or UAP56–RNA–TREX-2^M^ (right) (stick representation). Superpositions of the wedge loop anchoring tyrosine and the central arginine of SAC3D1 or GANP are shown in the middle. GANP, light blue; other colours as in **a**. **h**, Cartoon model of UAP56–RNA–TREX-2 or TREX-2-like complex interactions, including RNA release from UAP56. Left to right: (1) pre-RNA release state: TREX-2 and TREX-2-like complexes bind RNA-clamped UAP56; (2) post-RNA release state: RNA is unclamped from UAP56, leaving UAP56 in an open conformation bound to the TREX-2 or TREX-2-like complex; (3) dissociation.

**Fig. 2:**
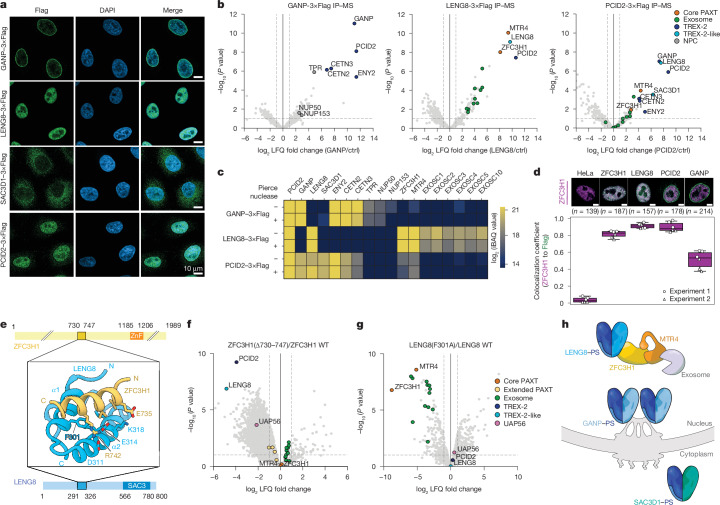
LENG8–PS constitutes a TREX-2-like module of PAXT. **a**, Immunofluorescence analyses of central TREX-2 and TREX-2-like components. Anti-Flag antibody (left column)- and DAPI (mid column)-stained HeLa cell lines (merged signals, right column), expressing C-terminally 3×Flag-tagged endogenous GANP (top row), LENG8 (second row), SAC3D1 (third row) or PCID2 (bottom row). Scale bars, 10 μm. **b**, Volcano plots of Flag IP–MS analyses of extracts from 3×Flag-tagged GANP (left), LENG8 (mid) or PCID2 (right) cells from **a**. log_2_ fold label-free quantification (LFQ) changes of interactor signals in the individual immunoprecipitations over their maternal HeLa cell line control were plotted against −log_10_-transformed two-sided limma-moderated Student’s *t*-test *P* values calculated over biological triplicate data. TREX-2-like, core PAXT, exosome, TREX-2 and NPC components are colour-coded and labelled. **c**, Heat map of mean intensity-based absolute quantification (iBAQ) values, with control immunoprecipitation values subtracted, from triplicate immunoprecipitation experiments with 3×Flag-tagged GANP, LENG8 and PCID2, conducted without (−) or with (+) Pierce universal nuclease treatment. Displayed proteins as in **b**, and with EXOSC1–EXOSC5 and EXOSC10. **d**, Colocalization coefficients between ZFC3H1 and Flag immunofluorescence signals in maternal HeLa cells (from Extended Data Fig. 4j) and HeLa cells expressing 3×Flag-tagged ZFC3H1, LENG8, PCID2 or GANP. Red (displayed in magenta) and green channels were used for the detection of ZFC3H1 and Flag signals, respectively. Example cells with staining overlap and total numbers (*n*) of cells imaged are indicated above the plot. In all box plots, the centre line is the median, box edges delineate the interquartile range and whiskers represent the distribution of data points within 1.5× interquartile range (Methods). **e**, AlphaFold2 model of interacting regions of ZFC3H1 (top) and LENG8 (bottom), shown in cartoon representation with interface residue as sticks (middle). The conserved F301 of LENG8, which is critical for interaction, is highlighted. **f**, Volcano plot as in **b**, but displaying ZFC3H^Δ730−747^–3×Flag relative to wild-type ZFC3H1–3×Flag sample data. Constructs were expressed in HeLa cells expressing ZFC3H1–2×HA–dTAG that were treated with dTAG^V^-1 to deplete endogenous ZFC3H1. Note additional colour coding of extended PAXT components and UAP56 (see **g**). WT, wild type. **g**, As in **f**, but for LENG8(F301A)–3×Flag relative to wild-type LENG8–3×Flag constructs expressed in dTAG^V^-1-treated cells expressing LENG8–2×HA–dTAG, **h**. Cartoon depicting localization-distinct TREX-2-like modules.

To mediate the docking of export-competent pA^+^ RNPs at the NPC, UAP56 binds the five subunit TREX-2 complex^7^ (GANP, PCID2, SEM1, CETN2 or CETN3, and ENY2). Within this complex, UAP56 contacts the TREX-2 complex core (TREX-2^M^), which comprises PCID2, SEM1 and the SAC3 domain of the scaffolding subunit GANP^7^ (Fig. 1a, left). The ability of TREX-2^M^ to release UAP56 from the pA^+^ RNP depends on the conserved ‘wedge loop’ within the SAC3 domain^7,17^ (Fig. 1b). Notably, similar SAC3 domains are found in the UAP56-interacting LENG8 and SAC3D1 proteins^7^. Although they are broadly conserved amongst eukaryotes, these proteins share no sequence features with GANP or each other aside from the SAC3 domain (Fig. 1a,b). Moreover, proteome-wide AlphaFold2 screens suggested that SAC3 domains of LENG8 or SAC3D1 form complexes with PCID2 and SEM1^18–20^, thus mimicking TREX-2^M^. Finally, and central to the present study, LENG8 co-immunoprecipitated with PAXT core components ZFC3H1 and MTR4^8,21^ (also see Fig. 2 below) and was shown to interact with PCID2 and SEM1 in both human and yeast cells^22,23^. Collectively, this prompted us to investigate these TREX-2^M^-like complexes in more detail.

**Extended Data Fig. 1:**
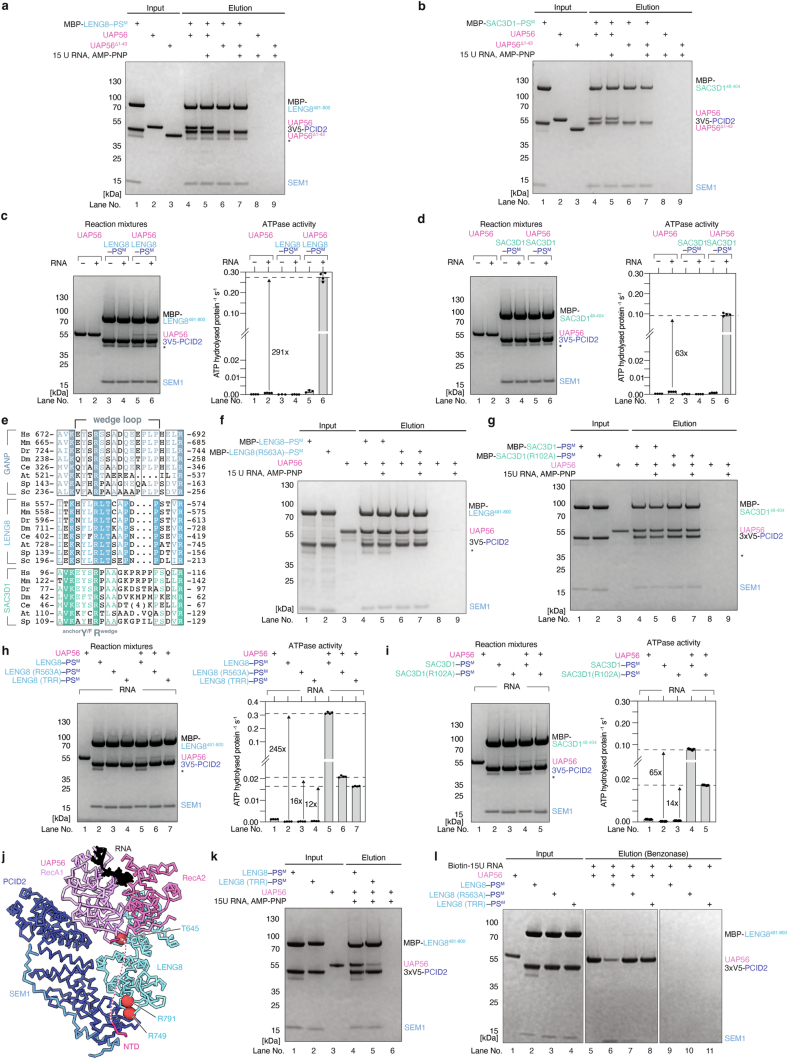
Biochemical characterization of TREX-2-like complexes. **a**,**b**, Coomassie-stained SDS-PAGE gels of in vitro pulldown experiments of LENG8–PS^M^ **a**, or SAC3D1–PS^M^ **b**. Recombinant MBP-LENG8–PS^M^ or MBP-SAC3D1–PS^M^ were immobilized on amylose beads through the MBP-tag and incubated with UAP56, or UAP56 ^Δ1-43^, and with or without 15 nucleotide poly-uridine RNA and AMP-PNP as indicated. After washes, bead-bound proteins were eluted with maltose containing buffer. *Denotes contaminant. **c**,**d**, ATPase assays demonstrating that LENG8–PS^M^ **c**, or SAC3D1–PS^M^ **d**, stimulate the apparent ATPase activity of RNA-bound UAP56. The broken Y-axes show ATPase rates (mean ± SD), the fold stimulations of which are indicated. Note presence (+) or absence (−) of a 15 poly-uridine RNA substrate. Left panels are Coomassie-stained SDS-PAGE gels of the samples used in the respective ATPase assays. *Denotes contaminant. Importantly, the apparent ATPase rate readout does not resolve which step of the ATPase cycle [(i) binding of ATP and RNA, ii) ATP hydrolysis and iii) release of ADP, inorganic phosphate, and RNA] is being stimulated. UAP56 forms highly stable ‘clamped’ complexes with RNA, that can persist for more than 30 min at room temperature^7^. In these complexes, ATP is hydrolysed immediately upon RNA binding, such that UAP56 predominantly exists in an ADP·Pi-bound state rather than an ATP-bound state^7^. Consequently, the apparent ATPase activity of UAP56 in the presence of RNA is low, because the enzyme remains associated with the same RNA molecule for prolonged periods. The rate-limiting step in the disassembly of the UAP56–RNA complex is the release of ADP and Pi from UAP56. TREX-2 and the related complexes described in the present study weaken the interaction between UAP56 and the bound nucleotide, thereby promoting release of both nucleotide and RNA. This action on the rate limiting step in the ATPase cycle leads to the observed apparent ATPase rate increase. **e**, Multiple sequence alignment of wedge loop sequences of GANP (top), LENG8 (middle) and SAC3D1 (bottom) proteins from selected model species. Amino acids were coloured by conservation (coloured letter, conserved residue; colored background, invariant residue). GANP proteins are from *H. sapiens* (Hs, Uniprot ID O60318), *M. musculus* (Mm, Q9WUU9), *D. rerio* (Dr, F1Q712), *D. melanogaster* (Dm, Q9U3V9), *C. elegans* (Ce, Q19643), *A. thaliana* (At, F4JAU2), *S. pombe* (Sp, O74889) and *S. cerevisiae* (Sc, P46674); LENG8 proteins are from *H. sapiens* (Hs, Uniprot ID Q96PV6), *M. musculus* (Mm, Q8CBY3), *D. rerio* (Dr, A4QNR8), *D. melanogaster* (Dm, B3DNL8), *C. elegans* (Ce, Q8I7L2), *A. thaliana* (At, A0A178VUB3), *S. pombe* (Sp, Q1MTP1) and *S. cerevisiae* (Sc, Q12049); SAC3D1 proteins are from *H. sapiens* (Hs, A6NKF1), *M. musculus* (Mm, A6H687), *D. rerio* (Dr, A0AB13A8D7), *D. melanogaster* (Dm, Q9VSZ4), *C. elegans* (Ce, Q8WQE7), *A. thaliana* (At, Q67XV2) and *S. pombe* (Sp, Q9USI4). **f**,**g**, In vitro pulldown assay as in **a**,**b**, but including the wedge loop mutans for LENG8 (R563A, **f**) and SAC3D1 (R102A, **g**). *Denotes contaminant. **h**,**i**, ATPase assays as in **c,d**, but including the wedge loop mutants for LENG8 (R563A, **h**) and SAC3D1 (R102A, **i**) and in addition a UAP56 binding mutant of LENG8 (‘TRR’). Left panels are Coomassie stained SDS-PAGE gels showing aliquots of the samples used in the assays. *Denotes contaminant. **j**, Design of the UAP56 binding mutant of LENG8 (LENG8 ‘TRR’). Both the interface between LENG8 and the RecA2 lobe of UAP56 (introducing a steric hinderance through T645W) and the NTD interacting interface (preventing charged interactions through R749D and R791D) were targeted. Mutated residues are shown as red spheres on a model of the UAP56–LENG8-PS complex (Cα trace). **k**, Coomassie-stained SDS-PAGE gel of in vitro pulldown experiments as in **a**, but for LENG8-PS^M^ complexes of LENG8 wild type or TRR mutant origins. **l**, UAP56 release assays as shown in Fig. 1c, but including the LENG8 wedge loop (R563A)- or UAP56 binding (TRR)-mutants.

Given the critical role of UAP56 in pA^+^ RNP export via TREX-2, we hypothesized that LENG8 and SAC3D1 might target UAP56-bound RNPs to different cellular fates. To address this, we first explored the structure–function relationships of LENG8 or SAC3D1 with UAP56 in vitro. As previously achieved for GANP^7^, we purified stable recombinant complexes of the SAC3 domain-containing constructs of LENG8^491–800^ or SAC3D1^48–404^ in the presence of PCID2–SEM1 (constituting LENG8–PS^M^ or SAC3D1–PS^M^; Supplementary Fig. 2a,b). Both complexes could bind UAP56 in the presence of the non-hydrolysable ATP analogue adenylyl-imidodiphosphate (AMP-PNP) and a 15-nucleotide poly-U RNA substrate (Extended Data Fig. 1a,b, lanes 1–5). TREX-2^M^ facilitates the release of ADP and Pi from UAP56, thus accelerating the rate-limiting step in the disassembly of UAP56–RNA complexes, releasing free UAP56 available for RNA re-binding, and resulting in an increased apparent ATPase activity^7^. Similarly, the LENG8–PS^M^ or SAC3D1–PS^M^ complexes stimulated the apparent ATPase rate of UAP56 in the presence of RNA and ATP in vitro, revealing approximately a 290-fold and 60-fold stimulation, respectively (Extended Data Fig. 1c,d). Moreover, substituting a highly conserved arginine residue in the LENG8 or SAC3D1 wedge loops with an alanine^7^ (LENG8(R563A) or SAC3D1(R102A)) (Fig. 1b, Extended Data Fig. 1e and Supplementary Fig. 2a,b), did not affect UAP56 binding (Extended Data Fig. 1f,g and Supplementary Fig. 2c), but largely abrogated the ATPase stimulatory activity on UAP56 (Extended Data Fig. 1h,i). Finally, to test whether LENG8–PS^M^ and SAC3D1–PS^M^, like TREX-2^M^ (ref. ^7^), would promote the release of RNA from UAP56, we incubated UAP56 with RNA and ATP to form UAP56–ADP-P_i_–RNA complexes, which we immobilized on streptavidin beads via the biotinylated RNA^7^. These complexes were then challenged with either LENG8–PS^M^ or SAC3D1–PS^M^, revealing that both moieties released UAP56 efficiently (Fig. 1c,d, compare lanes 4 and 5), whereas the respective wedge loop mutants did not (Fig. 1c,d, lane 6). Of note, mutating three residues targeting the UAP56 N-terminal domain (NTD) and UAP56 RecA2-binding interfaces of LENG8 (LENG8(TRR)) led to diminished LENG8–PS^M^–UAP56 interaction (Extended Data Fig. 1j,k and Supplementary Fig. 2a) and parallel declines in both the apparent ATPase activity (Extended Data Fig. 1h, lane 7) and the release of UAP56 from RNA (Extended Data Fig. 1l). We conclude that TREX-2-like complexes, like TREX-2, bind UAP56 and trigger the release of its bound RNA through a shared mechanism.

**Extended Data Fig. 2:**
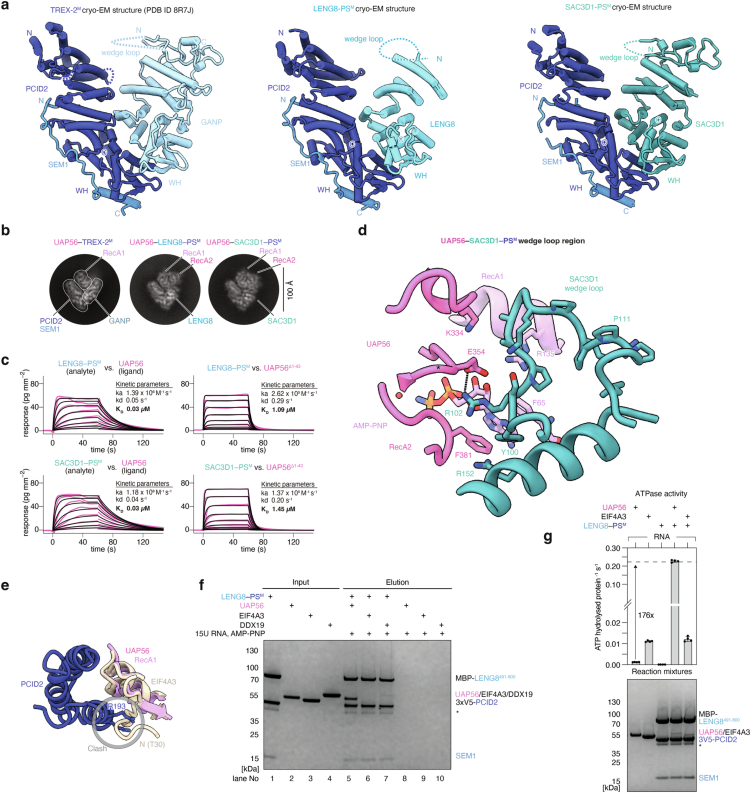
Biochemical characterization of TREX-2-like complexes. **a**, Cryo-EM structures of TREX-2^M^ (PDB ID 8R7J) (ref. ^7^) (left), LENG8^491–800^–PCID2–SEM1 (centre) and SAC3D1^48–404^–PCID2–SEM1 (right) in cartoon representations. PCID2, dark blue; SEM1, blue; GANP, light blue; LENG8, mid blue; SAC3D1, green blue. **b**, Representative cryo-EM 2D classes for UAP56-TREX-2^M^ (left, ref. ^7^), UAP56-LENG8–PS^M^ (middle) and UAP56-SAC3D1–PS^M^ (right), showing UAP56 in the RNA-clamped conformation, when in complex with either LENG8–PS^M^ or SAC3D1–PS^M^, but not with TREX-2^M^. **c**, Grating-coupled interferometry (GCI) assays employing LENG8–PS^M^ (top) or SAC3D1–PS^M^ (bottom) complexes immobilized as the analyte on a microfluidic chip and with UAP56 (left), or its UAP56^D1-43^ variant (right), flown in at increasing concentrations as the ligand. Shown are sensograms (pink lines), the fitted model (black lines) and a binding kinetics summary table. **d**, Stick and cartoon representation of the SAC3D1 wedge loop-UAP56-nucleotide interactions for UAP56-RNA-SAC3D1–PS^M^. **e**, Cartoon showing PCID2 (dark blue) and UAP56 (pink) as positioned in the UAP56–SAC3D1-PS^M^ structure, and EIF4A3 from PDB ID 7ZNJ, chain A (brown). EIF4A3 was superimposed onto UAP56 RecA1 (RMSD = 0.87 Å across 189 atom pairs), revealing clashes between EIF4A3 residues 30-35 and PCID2. **f**, Coomassie-stained SDS-PAGE gel of in vitro pulldown experiments demonstrating that UAP56, but not the related DExD-box ATPases EIF4A3 or DDX19, interacts with LENG8–PS^M^. Recombinant MBP-LENG8–PS^M^ was immobilized on amylose beads through the MBP tag and incubated with UAP56, EIF4A3 or DDX19. After washes, bead-bound proteins were eluted with maltose containing buffer. *Denotes contaminant. **g**, ATPase assays demonstrating that LENG8–PS^M^ stimulates the apparent ATPase activity of RNA-bound UAP56, but not EIF4A3. The broken Y-axes show ATPase rates (mean ± SD), the fold stimulations of which are indicated. Note that due to its high intrinsic ATPase activity DDX19 could not be tested in this experiment. Shown below is a Coomassie-stained SDS-PAGE gel of the samples used in the ATPase assays. *Denotes contaminant.

**Extended Data Fig. 3:**
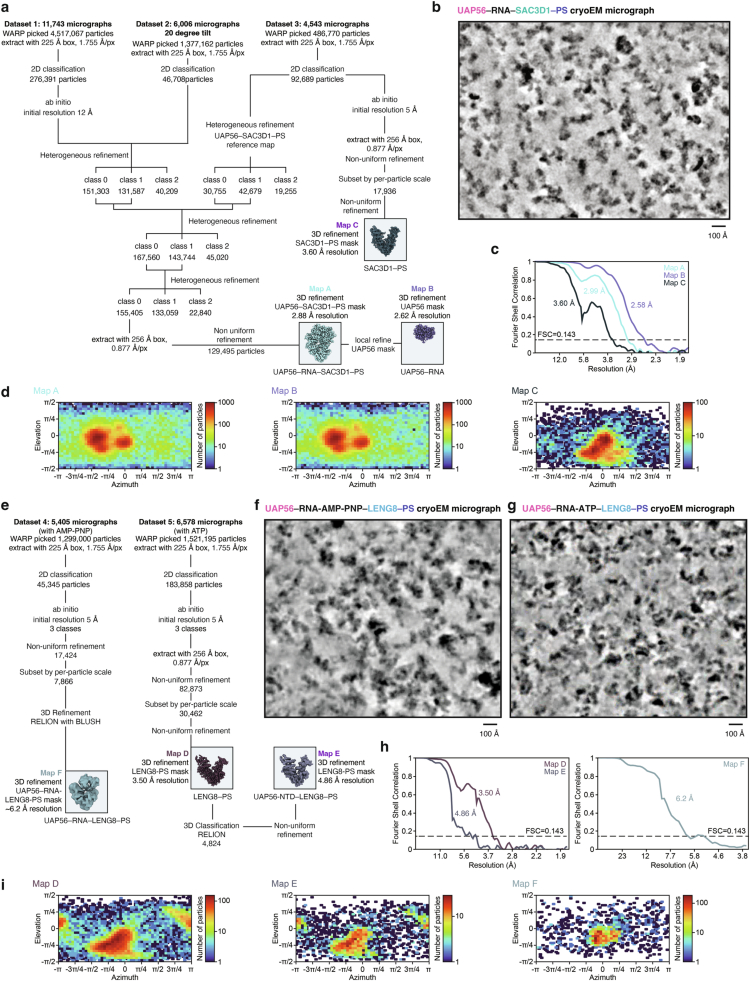
Cryo-EM data processing. **a**, Three-dimensional image classification tree for UAP56–RNA-AMP-PNP–SAC3D1–PS. From three datasets, containing 11,743, 6,006 (collected at 20-degree stage tilt) and 4543 micrographs, 4,517,067, 1,377,162 and 486,770 particles were picked through a BoxNet in WARP and processed in cryoSPARC. The final particle stack contained 126,495 particles and was refined to 3.0 Å resolution for the UAP56–RNA–SAC3D1–PS complex (Map A). A focused refinement using a mask comprising UAP56 and the bound RNA yielded a 2.6 Å density of UAP56–AMP-PNP–RNA (Map B). Ab initio reconstruction considering high spatial frequencies (see Methods) on dataset 3 yielded an interpretable cryo-EM map for a SAC3D1–PS complex, the final particle stack of which contained ~18 k particles and was refined to 3.60 Å resolution (Map C). **b**, Denoised cryo-EM micrograph of the UAP56–RNA–SAC3D1–PS sample. Scale bar, 100 Å. **c**, Gold-standard Fourier shell correlation (FSC = 0.143) of the UAP56–RNA–SAC3D1–PS complex (Map A), UAP56–AMP-PNP–RNA (Map B) and SAC3D1–PS complex (Map C) cryo-EM maps. **d**, cryoSPARC orientation distribution plots for UAP56–RNA–SAC3D1–PS (Map A), UAP56–AMP-PNP–RNA (Map B) and SAC3D1–PS complex (Map C) cryo-EM maps. **e**, Three dimensional image classification tree for UAP56–RNA-LENG8–PS with ATP (dataset 4) or AMP-PNP (dataset 5). From the two datasets, containing 15,405 and 6578 micrographs, 1,299,000 and 1,521,195 particles were picked through a BoxNet in WARP and processed in cryoSPARC and RELION. The final particle stacks contained 30,462 particles, refined to 3.50 Å, for the LENG8–PS complex (Map D) and 7,866 particles, refined to ~6.2 Å resolution for the UAP56–RNA–LENG8–PS complex (Map F). Further 3D classification of the LENG8–PS particles in RELION yielded a subset of 4,824 particles, which refined to ~4.9 Å, with clear UAP56 NTD density (UAP56-NTD–LENG8–PS, Map E). **f**,**g**, Denoised cryo-EM micrographs of the UAP56–RNA–SAC3D1–PS sample in the presence of AMP-PNP (**f**) and ATP (**g**). Scale bar, 100 Å. **h**, Gold-standard Fourier shell correlation (FSC = 0.143) of the LENG8–PS complex (Map D), UAP56-NTD–LENG8–PS complex (Map E) and UAP56–RNA–LENG8–PS complex (Map F) cryo-EM maps. **i**. cryoSPARC orientation distribution plots for LENG8–PS (Map D), UAP56-NTD–LENG8–PS (Map E) and UAP56–RNA–LENG8–PS (Map F) cryo-EM maps.

Although previous structural studies of UAP56–TREX-2^M^ complexes had revealed their protein–protein interfaces, it remained unclear how the wedge loop functions in releasing UAP56 from RNA. To investigate the molecular basis for this function, we analysed LENG8–PS^M^ and SAC3D1–PS^M^ complexes with UAP56 in the presence of 15-nucleotide poly(U) RNA and ATP or AMP-PNP using cryo-electron microscopy (cryo-EM). This revealed a fraction of complexes without UAP56, enabling us to solve the structures of apo LENG8–PS^M^ and SAC3D1–PS^M^ at 3.5 Å and 3.6 Å resolution, respectively (Extended Data Table 1). Both complexes showed the same V-shaped architecture previously observed for TREX-2^M^ (Extended Data Figs. 2a and 3 and Supplementary Figs. 3a–c and 4a–c) and a yeast LENG8–PS^M^ complex^23,24^. Unexpectedly, two-dimensional class averages of the UAP56-engaged fractions of LENG8–PS^M^ and SAC3D1–PS^M^ suggested that UAP56 could be in a closed, RNA-bound state, prior to its release via the wedge loop (Extended Data Fig. 2b). Together with our previously reported UAP56–TREX-2^M^ structure^7^, in which UAP56 was captured after RNA release, this enabled us to investigate the RNA-releasing mechanism of SAC3 domain-containing complexes. We resolved the cryo-EM structures of UAP56–LENG8–PS^M^ and UAP56–SAC3D1–PS^M^ complexes in the pre-RNA release state (Fig. 1e,f, Extended Data Fig. 3, Supplementary Figs. 3d–g and 4f,g and Extended Data Table 1). A severe bias in particle orientation limited resolution to 6–12 Å for UAP56–LENG8–PS^M^ in the RNA-clamped pre-release state. Reconstitution of the complex with ATP yielded a higher resolution structure at 4.9 Å containing density only for the UAP56 NTD (Supplementary Fig. 4d,e). We could, however, resolve the structure of UAP56–RNA–SAC3D1–PS^M^ to 2.6 Å, enabling a detailed structural analysis. The structure of the pre-release state shared key architectural features with UAP56–TREX-2^M^, including the anchoring of the NTD of UAP56 at the base of the SAC3D1–PS complex. Truncating the UAP56 NTD reduced the affinity of UAP56 for both SAC3D1–PS^M^ and LENG8–PS^M^ by more than 30-fold, as measured by grating-coupled interferometry (Extended Data Fig. 2c) and supported by in vitro pulldown assays (Extended Data Fig. 1a,b, lanes 6 and 7). Thus, the UAP56 NTD is equally important for TREX-2-like complex and TREX-2-complex^7^ interactions. In addition, the UAP56–SAC3D1–PS^M^ structure provided insights into the action of the wedge loop. In the structure, this region (residues Y100–P111; Fig. 1b and Extended Data Fig. 1e) is bound near the two RecA lobes through largely electrostatic interactions between the peptide backbone and UAP56 residues R135 in RecA1 and K334 in RecA2 (Fig. 1g and Extended Data Fig. 2d). The critical R102 wedge loop residue in SAC3D1 forms a hydrogen bond with UAP56 E354, positioning R102 close to F381 in the RecA2 lobe of UAP56. By contrast, in the post-release state observed for UAP56–TREX-2^M^, this central wedge loop arginine (R102 in SAC3D1, R678 in GANP) replaced UAP56 F381 in the nucleotide binding site (Fig. 1g, right). The positioning of the wedge loop arginine in the clamped state might prime it to replace UAP56 F381 in a subsequent step, releasing RNA from UAP56 (Fig. 1g,h and ref. ^7^).

The RNA-clamped RecA lobes of UAP56 are bound between PCID2, the wedge loop and the SAC3 domain in these SAC3 domain-containing complexes. Notably, the protein–protein interfaces between UAP56 and PCID2 in both TREX-2^M^ and the TREX-2^M^-like complexes involve only few specific interactions, except for the UAP56 NTD^7^, suggesting that PCID2–SEM1 has an architectural role in ensuring specificity for UAP56. Indeed, superposition of the evolutionarily related and RNA-bound form of the DExD-box ATPase EIF4A3^16^ onto the UAP56–SAC3D1–PS^M^ structure revealed clashes between EIF4A3 and PCID2 (Extended Data Fig. 2e). Consistently, LENG8–PS^M^ bound UAP56, but not the closely related DExD-box proteins EIF4A3 and DDX19 in vitro (Extended Data Fig. 2f and Supplementary Fig. 2d) and did not stimulate the EIF4A3 ATPase (Extended Data Fig. 2g).

We conclude that human cells contain three structurally and biochemically equivalent SAC3 domain-containing complexes. Aided by their complex architecture and the uniqueness of the UAP56 NTD, they all target UAP56 specifically and their conserved wedge loops can dislodge UAP56 from RNA.

## LENG8–PS provides a physical module for PAXT

**Extended Data Fig. 4:**
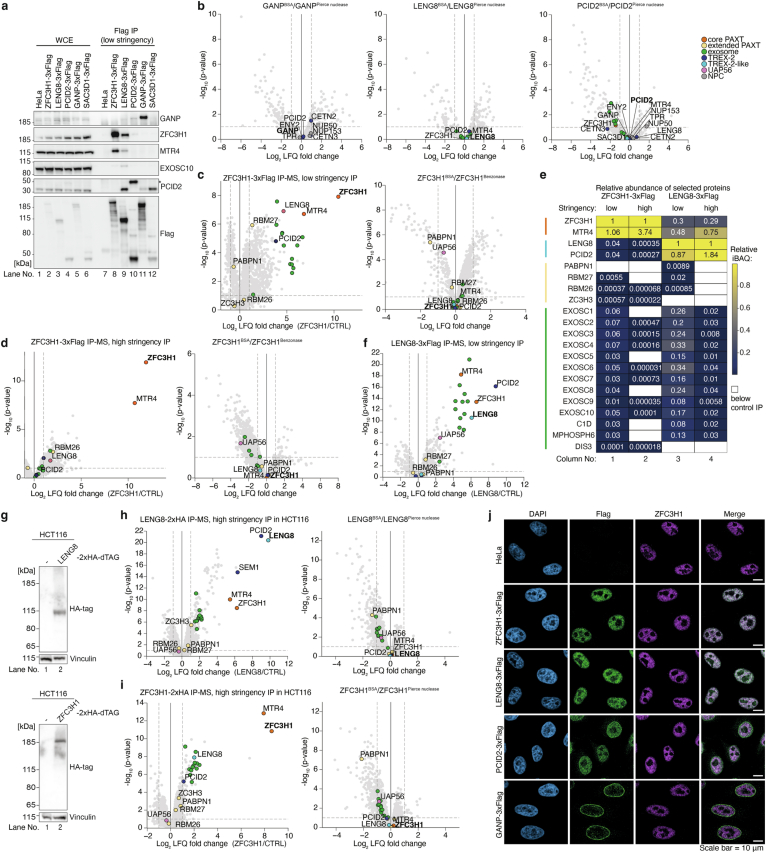
Multiple TREX-2-like complexes in human cells. **a**, Western blotting analysis of whole cell extracts (WCE, left part) or eluates after anti-Flag IP (right part) of extracts from endogenously 3×Flag-tagged ZFC3H1, LENG8, PCID2, GANP or SAC3D1 cells. Maternal HeLa cells were included as a negative control. Asterisk denotes the 3×Flag-tagged PCID2 band on the anti-PCID2 blot. Membranes were probed with GANP-, ZFC3H1-, MTR4-, EXOSC10-, PCID2- and Flag-specific Abs as indicated. For gel source data for this and all subsequent western blotting figures, see Supplementary Fig. 1. **b**, Volcano plots related to Fig. 2b, but showing log_2_ fold changes of LFQ values of interactors in Pierce universal nuclease-treated relative to non-treated IP samples of the indicated bait proteins (x-axes). **c**, Volcano plot as in Fig. 2b, but showing ZFC3H1–3×Flag relative to control IP (left panel) or nuclease-treated relative to non-treated IP (right panel) in low stringency IP condition. Note that Benzonase was employed instead of Pierce nuclease. **d**, Volcano plots as in **c**, but under high stringency IP condition. **e**, Heatmap showing the relative abundance, according to iBAQ values, of selected HeLa cell proteins from ZFC3H1–3×Flag (columns 1-2) or LENG8–3×Flag (columns 3-4) IPs conducted in nuclease-containing conditions at low- or high-stringency as indicated. Colour-coding of proteins as in **b**. **f**, Volcano plot as in Fig. 2b, but showing LENG8–3×Flag low stringency IP. **g**, Western blotting analysis as in **a**, but showing HCT116 cells harbouring 2×HA-tagged-LENG8 (top), or -ZFC3H1 (bottom). Membranes were probed with anti-HA and vinculin Abs. **h**, Volcano plot as in **c**, but for LENG8 high stringency anti-HA IPs from HCT116 WCE. **i**, Volcano plot as in **c**, but for ZFC3H1 high stringency anti-HA IPs from HCT116 WCE. **j**, Immunofluorescence analysis as in Fig. 2a. Shown are DAPI (left column)-, anti-Flag (left-mid column)-and anti-ZFC3H1 (right-mid column)-stained HeLa cell lines, expressing the indicated C-terminally 3×Flag tagged proteins. Right column shows merges of FLAG and ZFC3H1 signals. Maternal HeLa cells are shown as a negative control for the Flag IF. Related to Fig. 2d.

Our biochemical and structural analyses suggested that the GANP–PS, LENG8–PS and SAC3D1–PS complexes can individually act on UAP56. To address where these complexes act in vivo, we generated HeLa cell lines^25^ stably expressing C-terminally 3×Flag-tagged versions of endogenous GANP, LENG8 or SAC3D1 as well as the common subunit PCID2 (Extended Data Fig. 4a, lanes 3–6), and analysed these with immunofluorescence microscopy using a Flag antibody. As previously reported, GANP was found primarily at the nuclear envelope, consistent with its NPC association^6,26^ (Fig. 2a, top row). By contrast, LENG8 and SAC3D1 localized to the nucleoplasm and the cytoplasm, respectively (Fig. 2a, second and third rows). In agreement with its presumed presence in all three complexes, PCID2 was distributed between the nucleoplasm, the nuclear envelope and the cytoplasm (Fig. 2a, bottom row).

**Extended Data Fig. 5:**
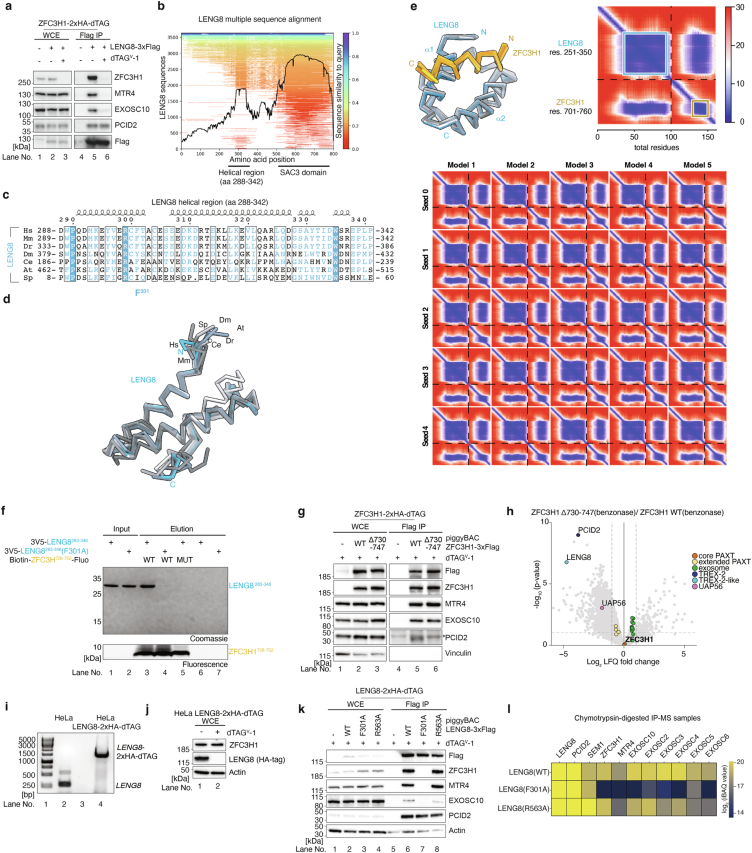
LENG8-PS interaction with PAXT and the exosome is mediated by direct binding of ZFC3H1 and LENG8. **a**, Western blotting analysis of WCE (left part) or eluates of anti-Flag IP (right part) of extracts from HeLa cells, expressing endogenously dTAG-tagged ZFC3H1, and which were not (−) or transiently transfected (+) with plasmid expressing 3×Flag-tagged LENG8, without or in presence of dTAG^V^-1 as indicated. Membranes were probed with ZFC3H1, MTR4, EXOSC10, PCID2 and Flag-tag -specific Abs as indicated. **b**, Multiple sequence alignment of *H. sapiens* LENG8 protein with LENG8 sequences from the AlphaFold2 MMseq2 database. Conserved helical regions and SAC3 domains are indicated by black lines. **c**, Multiple sequence alignment as in Extended Data Fig. 1e, but of the conserved region of human LENG8^288–342^. The phenylalanine (F301) critical for interaction with ZFC3H1 is highlighted. **d**, Superposition of the ZFC3H1-binding domain of LENG8 (residues 288-342 for HsLENG8) from model species from **c** and shown as Cα trace. The RMSD with the domain of human LENG8 is 0.56 Å for *M. musculus*, 0.35 Å for *D. rerio*, 0.69 Å for *D*. *melanogaster*, 0.67 Å for *C. elegans*, 0.64 Å for *A. thaliana* and 0.96 Å for *S. pombe* LENG8. *H*. *sapiens* LENG8, blue; other species, shades of grey. **e**, Predicted Alignment Error (PAE) plots for AlphaFold2 predictions of LENG8 residues 251-350 and ZFC3H1 residues 701-760. Shown is one annotated PAE plot (top right) with the interacting regions of LENG8 and ZFC3H1 highlighted with blue and orange boxes respectively, as well as all PAE plots for the 25 predicted models (bottom). Shown (top left) is the superposition of all 25 predicted models (Cα trace), shown are only the confidently predicted parts LENG8 res. 289-342 and ZFC3H1 res. 733-747; ZFC3H1 prediction 1-25 shades of orange; LENG8 predictions 1-25 shades of blue or grey. RMSDs range from 0.16 Å to 1.1 Å between predictions. Related to Fig. 2e. **f**. Anti-streptavidin pulldown of C-fluorescein-labelled Biotin-ZFC3H1^728–752^ wildtype (WT, lanes 3-4) or mutant (MUT, lane 5) peptides incubated with recombinant LENG8^283–346^ WT- (lane 1,3,5) or F301A- (lane 2,4) MUT peptides. Eluates were analysed by SDS-PAGE and Coomassie-staining. WT and MUT peptide sequences are listed in Supplementary Table 6. **g**, Western blotting analysis as in **a**, but of extracts from HeLa cells, expressing endogenously dTAG-tagged ZFC3H1 without or with piggyBac-integrated exogenous ZFC3H1(WT)–3×Flag or ZFC3H1(Δ730-747)–3×Flag variants as indicated. Membranes were probed with Flag-tag, ZFC3H1, MTR4, EXOSC10, PCID2 and vinculin-specific Abs as indicated. Asterisk indicates unspecific band. **h**, Volcano plot as in Fig. 2f, but showing ZFC3H1(Δ730-747)– relative to ZFC3H1(WT)–3×Flag benzonase-treated samples. **i**, PCR analysis with amplicon spanning the region of CRISPR-mediated modification of the *LENG8* gene, using DNA from maternal HeLa- or LENG8–2×HA–dTAG cells (see Methods). **j**, Western blotting analysis of WCE of LENG8–2×HA–dTAG cells untreated or treated with dTAG^V^-1 for 4 h. Membranes were probed with HA-tag for LENG8, ZFC3H1, actin-specific Abs as indicated. **k**, Western blotting analysis of WCE (left part) or of eluates from anti-Flag IP (right part) from extracts of HeLa cells with endogenously dTAG-tagged LENG8 without or with piggyBac-integrated exogenous LENG8(WT)–3×Flag, LENG8(F301A)- or LENG8(R563A)–3×Flag constructs. Membranes were probed with Flag-tag, ZFC3H1, MTR4, EXOSC10, PCID2 and actin -specific Abs as indicated. **l**, Heatmap as in Fig. 2c, but for LENG8(WT)- LENG8(F301A)-, or LENG8(R563A)–3×Flag IPs from **k**. MS was performed using chymotrypsin treated eluates to enable detection of SEM1. iBAQ values from control Flag IPs in HeLa were subtracted.

With our focus on nuclear RNA sorting, we examined the TREX-2 and LENG8–PS complexes in more detail, performing immunoprecipitation–mass spectrometry (IP–MS) analyses of the 3×Flag-tagged GANP, LENG8 and PCID2 proteins. Stringent immunoprecipitation conditions were used to enrich for high-affinity interactors (Methods). In the GANP–3×Flag immunoprecipitation, this yielded large amounts of PCID2, in addition to the ENY2 and CETN2 or CETN3 subunits of TREX-2^6^, and the nuclear pore basket protein TPR^26^ (Fig. 2b, left). Although LENG8–3×Flag also precipitated PCID2, this immunoprecipitation was instead enriched for the PAXT core components ZFC3H1 and MTR4 along with nuclear exosome subunits (Fig. 2b, middle; note that SEM1 was also detected applying an alternative protocol (Extended Data Fig. 5l)). Finally, reflecting its dual interaction with GANP and LENG8, PCID2–3×Flag precipitated these proteins and their respective interactors (Fig. 2b, right). Parallel, low-stringency immunoprecipitation–western blotting analyses qualitatively recapitulated these interaction patterns (Extended Data Fig. 4a, lanes 9–12) and subjecting the IP–MS experiments to RNase treatment revealed that the interactions were not facilitated by RNA (Extended Data Fig. 4b). Moreover, analysing mean enrichments over background across immunoprecipitation experiments displayed near-identical interaction levels of GANP with TREX-2 components, and LENG8 precipitated similar amounts of PCID2 as well as the core PAXT factors ZFC3H1 and MTR4 (Fig. 2c and Supplementary Table 1). This indicated that LENG8–PS complexes constitute a major module for PAXT. To address this notion further, we generated ZFC3H1–3×Flag cells (Extended Data Fig. 4a, lane 2) and conducted IP–MS experiments at both low and high stringency, which revealed RNase-resistant interactions with LENG8 and PCID2 (Extended Data Fig. 4c,d). Mean enrichments calculations showed that, along with exosome subunits, LENG8 and PCID2 were sub-abundant to MTR4, consistent with the reported presence of inactive nuclear ZFC3H1–MTR4 dimers^12^ (Extended Data Fig. 4e, columns 1 and 2). Nonetheless, the ZFC3H1 immunoprecipitations returned LENG8 and PCID2 in 5- to 10-fold excess of extended PAXT components and reciprocal LENG8 immunoprecipitations recovered abundant amounts of the ZFC3H1–MTR4 dimer (Extended Data Fig. 4e, columns 3 and 4, f). We further reproduced key high-stringency interactions in HCT116 cells, expressing endogenous LENG8–2×HA–dTAG or ZFC3H1–2×HA–dTAG proteins (Extended Data Fig. 4g–i and Supplementary Table 1). Finally, association of LENG8 with ZFC3H1 was mirrored by their measured nuclear colocalization, as revealed by immunostaining of LENG8–3×Flag cells using Flag- and ZFC3H1-specific antibodies. Here, LENG8 and PCID2, but not GANP, displayed weighed colocalization coefficients with ZFC3H1 in the nucleoplasm of more than 0.90 (Fig. 2d and Extended Data Fig. 4j).

Having established a physical link between LENG8 and ZFC3H1, we inquired whether LENG8 interacts with MTR4 and the exosome via ZFC3H1. Indeed, rapid ZFC3H1 depletion, using the FKBP12(F36V)–degron (dTAG)^27^ (Extended Data Fig. 5a, left), prevented interactions of MTR4 and the exosome component EXOSC10 with LENG8–3×Flag (Extended Data Fig. 5a, right). To identify a possible ZFC3H1 interaction site on LENG8, we utilized AlphaFold2, which revealed a conserved motif of two α-helices (residues 288–342) in the otherwise unstructured region N-terminal to the SAC3 domain (Extended Data Fig. 5b–d). A direct interaction was predicted between this helical region of LENG8 and a single α-helix (residues 730–747) of ZFC3H1 (Fig. 2e and Extended Data Fig. 5e), which we verified using an in vitro pulldown assay, comparing relevant wild-type or mutant versions of recombinant LENG8 and ZFC3H1 peptides (Extended Data Fig. 5f and Supplementary Fig. 2e). Further validating the interaction, IP–MS analysis of a ZFC3H1(Δ730–747) compared to a wild-type ZFC3H1 3×Flag construct demonstrated the specific loss of LENG8 and PCID2 over exosome subunits and extended PAXT components (Fig. 2f and Extended Data Fig. 5g) also upon nuclease treatment (Extended Data Fig. 5h). Similarly, mutation of the central LENG8 phenylalanine at the LENG8–ZFC3H1 interface (Fig. 2e) to an alanine (F301A) led to the selective loss of ZFC3H1, MTR4 and exosome subunits in a comparative IP–MS analysis of wild-type LENG8 versus LENG8(F301A) 3×Flag constructs (Fig. 2g, Extended Data Fig. 5i–l and Supplementary Table 1).

Together, we conclude that the TREX-2-like LENG8–PS complex constitutes a physical module of the nucleoplasmic PAXT connection (Fig. 2h).

## LENG8–PS works with PAXT

**Fig. 3:**
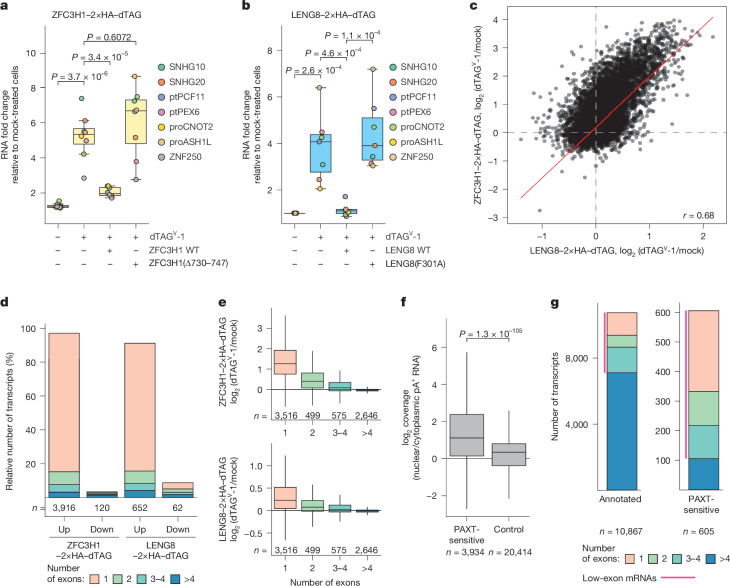
LENG8–PS provides a functional module for PAXT. **a**, Box plots of RT–qPCR analysis of selected PAXT substrates from cells expressing ZFC3H1–2×HA–dTAG and treated with (+) or without (−) dTAG^V^-1 for 4 h and complemented by expression of wild-type ZFC3H1 or ZFC3H1(Δ730–747) as indicated. The prefixes pt and pro indicate prematurely terminated and PROMPT, respectively. Values are relative to mock-treated HeLa cells and normalized to *GAPDH* RNA expression. Coloured dots denote the mean value of *n* = 3 biological replicates. Two-sided Bonferroni-corrected Student’s *t*-tests, calculated between conditions and combining the results for all RNAs tested. Similar statistical tests were conducted for the subsequent RT–qPCR analyses. **b**, As in **a** but for cells expressing LENG8–2×HA–dTAG and treated with (+) or without (−) dTAG^V^-1 for 24 h and complemented by expression of wild-type LENG8 or LENG8(F301A) as indicated. **c**, Scatter plot of mean log_2_ fold abundance changes in pA^+^ RNA of *n* = 3 biological replicates after treatment of cells expressing ZFC3H1–2×HA–dTAG (*y* axis) or LENG8–2×HA–dTAG (*x* axis) with 500 nM dTAG^V^-1 for 4 h. The trendline is shown in red and Pearson correlation coefficient (*r*) is indicated. **d**, Counts of differentially expressed pA^+^ RNAs after treatment of cells expressing ZFC3H1–2×HA–dTAG or LENG8–2×HA–dTAG with dTAG^V^-1 for 4 h. Transcripts are colour-coded by the number of exons. DESeq2 (adjusted *P* value < 0.1) was used and RNAs with log_2_ (fold change) greater than 0.5 and less than −0.5 were counted as upregulated and downregulated, respectively, here and in subsequent analyses. **e**, Box plots of log_2_ fold abundance changes in pA^+^ RNA from **c** after dTAG^V^-1 treatment of cells expressing ZFC3H1–2×HA–dTAG (top) or LENG8–2×HA–dTAG (bottom). All RNAs with measurable fold changes (*P* value < 0.1, per DESeq2) in either of the depletions were grouped by their contained exon numbers. Numbers of RNAs (*n*) examined are shown below the plots. **f**, Box plots of log_2_-scaled means of *n* = 3 biological RNA-seq replicates of nuclear/cytoplasmic ratios in unperturbed HeLa cells of upregulated ZFC3H1- or LENG8-sensitive pA^+^ RNAs compared to all other annotated RNAs. Two-sided unpaired Welch’s *t*-test. **g**, Counts of all annotated (left) or PAXT-sensitive (right) mRNAs stratified by contained exon numbers. PAXT sensitivity was scored as upregulation upon ZFC3H1 or LENG8 depletion. mRNAs containing 1 to 4 exons (classified as low-exon mRNAs) are marked with a pink bar.

**Extended Data Fig. 6:**
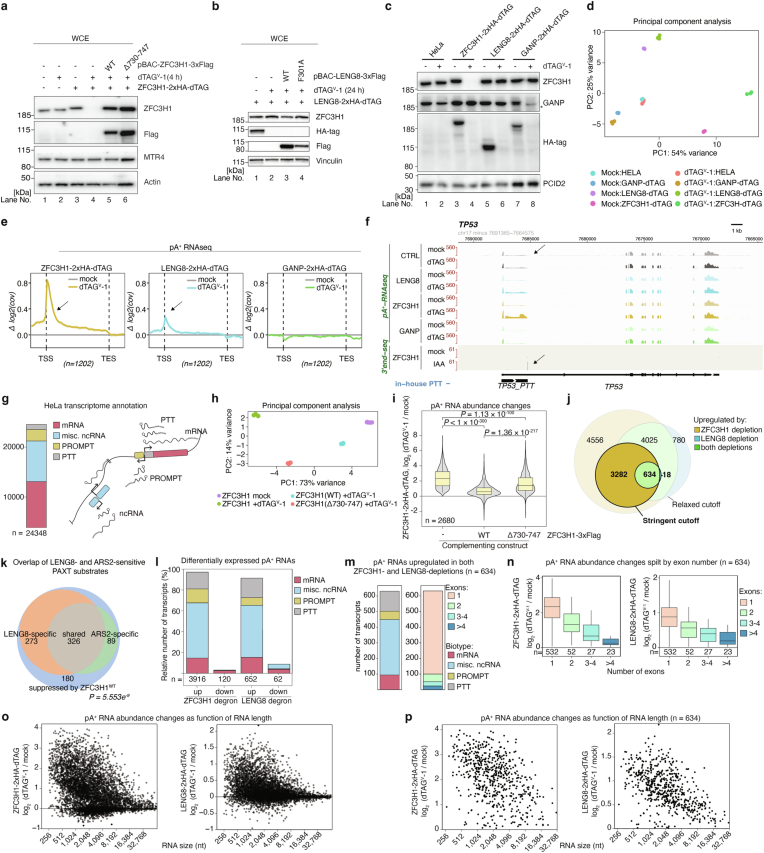
Interaction between ZFC3H1 and LENG8 is required to suppress pA^+^ RNA. **a**, Western blotting analysis of WCEs from maternal HeLa- or ZFC3H1–2×HA-dTAG-cells treated (+), or not (−), for 4 h with dTAG^V^-1. PiggyBac expression of ZFC3H1(WT)–3×Flag or ZFC3H1(Δ730-747)–3×Flag was conducted as indicated. Membranes were probed with ZFC3H1-, FLAG-, MTR4- and actin-specific Abs as indicated. Related to Fig. 3a. **b**, As in **a**, but employing LENG8–2×HA-dTAG cells subjected to 24 h of dTAG^V^-1 treatment (+), or not (−). LENG8 (WT)–, LENG8 (F301A)–3×Flag expression was conducted as indicated. Membranes were probed with ZFC3H1-, HA-, Flag- and vinculin-specific Abs as indicated. Related to Fig. 3b. **c**, Western blotting analysis of WCEs from maternal HeLa- or ZFC3H1-, LENG8- or GANP- 2×HA-dTAG cell lines mock treated (−) or treated for 4 h with dTAG^V^-1 (+) as indicated. Membranes were probed with ZFC3H1-, GANP-, HA- and PCID2-specific Abs as indicated. Truncated GANP isoform is indicated by an asterisk. **d**, Principal component analysis of obtained biological replicates of indicated pA^+^ RNAseq samples. **e**, Aggregate plots of Δlog_2_(coverage) signals of pA^+^ RNA-seq data from ZFC3H1–, LENG8– and GANP–2×HA-dTAG cell lines treated for 4 h with dTAG^V^-1 *vs*. their mock-treated controls. Signals were plotted over TUs expressing identified PTTs and displayed from 500 bp TSS-upstream to 500 bp TES-downstream regions (note arrows pointing to the TSS-proximal area with increased pA^+^ RNA coverage upon dTAG^V^-1 treatment). The TU body (TSS to TES) was normalized to 2 kb for each TU. Data are displayed with 90% confidence intervals (lighter shades around the curves). Numbers (n) of aggregated TUs are indicated. **f**, Genome browser view displaying pA^+^ RNAseq and 3’-RNAseq data, used in the PTT annotation pipeline, over the *TP53* gene with annotated *TP53* and *TP53 PTT* TUs. Arrows point to the TSS-proximal area with increased pA^+^ RNA coverage and to the pA^+^ 3’end of the 3’end-seq data (IAA, iodoacetamide). **g**, Left, simplified biotype overview of the 24348 annotated RNAs of the HeLa transcriptome. Right, cartoon representation of the annotated biotypes. **h**, Principal component analysis of obtained biological replicates of indicated pA^+^ RNAseq samples. **i**, Violin plots of pA^+^ RNA log_2_ fold abundance changes of RNAs upregulated upon ZFC3H1 depletion (per DEseq2, see Methods) after dTAG^V^-1 treatment for 4 h of ZFC3H1–2×HA-dTAG cells not expressing (left), expressing ZFC3H1(WT) (mid) or expressing the LENG8- binding deficient mutant ZFC3H1(Δ730-747) (right). Bonferroni-corrected p-values from two-sided t-tests performed between conditions are shown above the plots. **j**, Venn diagrams showing overlap of upregulated (per DESeq2, see Methods) pA^+^ RNAs between LENG8- and ZFC3H1-depletions using stringent (full colour, adj. p-value < 0.1 and log2 (fold change) > 0.5) or relaxed (low opacity colour in the background, log2(fold change) > 0.3) cutoffs. **k**, Venn diagram of ZFC3H1 substrates (see Methods), which were suppressed by WT ZFC3H1 expression (blue), but not by LENG8- (orange) or ARS2- (cyan) binding deficient ZFC3H1 mutants. The p-value of a Fisher exact test of the overlap between LENG8- and ARS2-specific substrates is shown below the Venn diagram. **l**, Distribution of biotypes as in **g**, but showing DESeq2 analysis of pA^+^ RNAseq data derived from ZFC3H1– and LENG8–2×HA-dTAG cells treated, or not, with dTAG^V^-1 for 4 h. **m**, Distribution of biotypes (left) and exon counts (right) for pA^+^ RNAs upregulated by both LENG8- and ZFC3H1- depletions using stringent cutoff (n = 634). Related to Fig. 3d. **n**, Boxplots as in Fig. 3e, but showing ZFC3H1- (left) or LENG8- (right) depletion pA^+^ RNA abundance changes for the co-regulated pA^+^ RNAs from **m**. Note, unlike in Fig. 3e, only upregulated RNAs are shown. **o**, Scatterplots of pA^+^ RNA log2 fold abundance changes after dTAG^V^-1 treatment for 4 h of ZFC3H1–(left) or LENG8 (right)–2×HA-dTAG cells as functions of log_2_-scaled lengths in the range 256 to 65,536 nucleotides (see Methods) of the respective processed RNAs. All transcripts with measurable fold changes (adj. p-value < 0.1) from DESeq2 analysis were included. **p**, Scatterplots as in **o**, but showing LENG8 and ZFC3H1 co-regulated pA^+^ RNAs from **m**.

Given the physical link between ZFC3H1 and LENG8, we next sought to probe its functional relevance. Expression of wild-type ZFC3H1–3×Flag, but not the ZFC3H1(Δ730–747) LENG8 binding-deficient variant (Extended Data Fig. 6a), suppressed selected formerly established PAXT substrates^8,14^ from accumulating after rapid depletion of endogenous ZFC3H1 (Fig. 3a). Similarly, PAXT substrate accumulation, following rapid depletion of endogenous LENG8, was suppressed by expression of wild-type LENG8–3×Flag but not by the ZFC3H1-binding mutant LENG8(F301A) (Fig. 3b and Extended Data Fig. 6b). Since these analyses corroborated a role for LENG8 in PAXT function, we obtained a transcriptome-wide view of this relationship by sequencing pA^+^ RNA from cells rapidly depleted for either LENG8 or ZFC3H1 (Extended Data Fig. 6c,d). PAXT substrates include numerous prematurely terminated transcripts (PTTs), deriving from transcription start site (TSS)-proximal regions of protein-coding genes^8,14^. To incorporate these in our analysis, we identified transcription units displaying such increased pA^+^ RNA coverage upon depletion of ZFC3H1 or LENG8 (Extended Data Fig. 6e) and intersected data with pA^+^ RNA 3′ end peaks previously identified upon depletion of ZFC3H1^28^ (Methods). As a result, 1,202 pA^+^ PTTs were defined (see Extended Data Fig. 6f for an example) and included in our HeLa transcriptome annotation^29^ (Extended Data Fig. 6g).

Although numbers and changed levels of affected RNAs were higher with ZFC3H1 depletion, individual LENG8- and ZFC3H1-sensitive transcripts were strongly correlated (Fig. 3c), consistent with a shared pathway. This was substantiated by sequencing pA^+^ RNA from ZFC3H1-depleted cells, exogenously expressing either wild-type ZFC3H1 or the LENG8-binding mutant ZFC3H1(Δ730–747) (Extended Data Fig. 6h); only wild-type ZFC3H1 efficiently suppressed the accumulation of ZFC3H1 substrates, whereas ZFC3H1(Δ730–747) retained only partial activity (Extended Data Fig. 6i). Moreover, differential expression analysis, applying DESeq2 (ref. ^30^) with two different cut-offs, revealed an extensive overlap of RNAs that were increased upon depletion of ZFC3H1 or LENG8 (Extended Data Fig. 6j and Supplementary Table 2). We speculated that the stronger effect size with ZFC3H1 depletion might, at least in part, reflect the interaction of ZFC3H1 with the early RNA-processing factor ARS2^14^, possibly allowing residual LENG8-independent ZFC3H1 activity. To test this, we compared ARS2- and LENG8-dependent PAXT targets by intersecting RNA-sequencing (RNA-seq) data from a ZFC3H1 complementation assay using the ZFC3H1(ARM) mutant, which cannot bind ARS2^14^, with RNA-seq data from the LENG8 binding-deficient ZFC3H1(Δ730–747) mutant. There was significant overlap between LENG8- and ARS2-dependent ZFC3H1 substrates, which suggested partially redundant LENG8 and ARS2 activities on a shared substrate pool (Extended Data Fig. 6k).

In agreement with previous studies on PAXT^8,11,13,14^, the ZFC3H1- and LENG8-depleted samples revealed upregulation of promoter upstream transcripts (PROMPTs)^31^, PTTs, other noncoding RNAs (ncRNAs) and a minor fraction of full-length mRNAs, both when each sample was evaluated individually (Extended Data Fig. 6l) and when interrogating the common substrate cohort (Extended Data Fig. 6m, left). In line with this result, the majority of ZFC3H1 and LENG8 substrates contained only one or a few exons (Fig. 3d and Extended Data Fig. 6m, right), and for both depletion conditions RNA accumulation levels decreased with increasing exon number (Fig. 3e and Extended Data Fig. 6n) and mature RNA length (Extended Data Fig. 6o,p).

**Extended Data Fig. 7:**
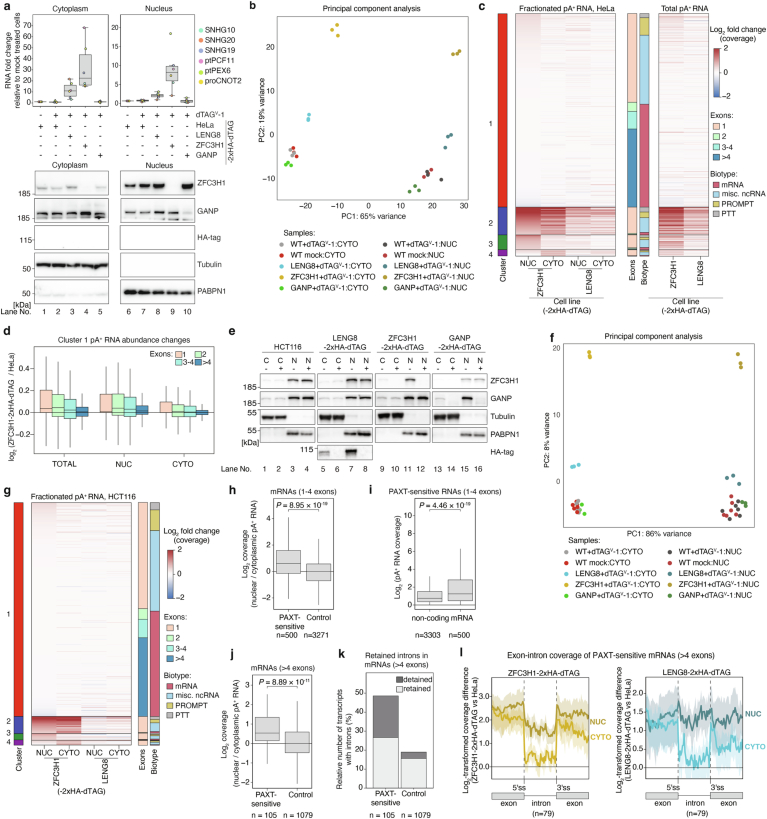
The LENG8-PAXT axis retains nuclear pA^+^ RNAs. **a**, Top: boxplots of RT-qPCR analysis of select PAXT substrates in nuclear and cytoplasmic fractions of biochemically fractionated maternal HeLa cells or the indicated 2×HA-dTAG-tagged cell lines with dTAG^V^-1-induced depletion of LENG8, ZFC3H1 or GANP for 4 h. RNA fold changes were calculated relative to values of non-treated HeLa control cells and normalized to GAPDH mRNA, separately for cytoplasmic (left) and nuclear (right) fractions. Dots indicate mean values of three biological replicates for each RNA. Bottom, Western blotting analysis of employed cytoplasmic (left) and nuclear (right) extracts. Membranes were probed with ZFC3H1-, GANP-, HA-, tubulin- and PABPN1-specific Abs as indicated. **b**, Principal component analysis of obtained biological replicates of indicated pA^+^ RNAseq samples. **c**, Heatmaps of pA^+^ RNA log_2_ fold abundance changes in nuclear (NUC) and cytoplasmic (CYTO) fractions (left panel) or total (right panel) samples of dTAG^V^-1-treated (4 h) ZFC3H1– or LENG8 –2×HA-dTAG cells relative to maternal HeLa cells. Transcripts were clustered based on NUC and CYTO abundance changes upon ZFC3H1 depletion with a cutoff log_2_ fold change of 0.5. Stacked bar plots in between heatmaps display transcript biotypes and contained exon numbers within each cluster. **d**, Boxplots of pA^+^ RNA log_2_ fold abundance changes upon ZFC3H1 depletion for all transcripts in Cluster 1 of panel **c**. Transcripts were grouped in compartments and colour-coded by exon numbers. **e**, Western blotting analysis of cytoplasmic (‘C’) and nuclear (‘N’) extracts from the indicated control and 2×HA-dTAG-tagged HCT116 cell lines used for RNA extraction followed by pA^+^ RNAseq. Membranes were probed with ZFC3H1-, GANP-, HA-, PABPN1- and tubulin-specific Abs as indicated. **f**, Principal component analysis as in **b**, but of pA^+^ RNAseq samples from HCT116 cells from **e**. **g**, Heatmaps as in **c**, left panel, but using pA^+^ RNAseq data from fractionated maternal HCT116 cells or HCT116 ZFC3H1- and LENG8–2×HA-dTAG cell lines treated with dTAG^V^-1. **h**, Boxplots of log_2_-scaled nuclear-cytoplasmic ratios of pA^+^ RNA log_2_ coverages in unperturbed HeLa cells for low-exon (1-4) PAXT-sensitive mRNAs and control mRNAs of the same exon numbers. A two tailed unpaired Welch’s t-test was calculated between groups of RNAs and the p-value displayed above the plot. **i**, Boxplots of pA^+^ RNA log_2_ levels of PAXT-sensitive ncRNAs or mRNAs (1-4 exons) in unperturbed HeLa cells. PAXT-sensitive transcripts were defined as in Fig. 3d. A two tailed unpaired Welch’s t-test was calculated between groups of RNAs and the p-value displayed above the plot. **j**, Boxplots of nuclear-cytoplasmic ratios as in **h**, but for PAXT-sensitive mRNAs (>4 exons) compared to control mRNAs of the same expression levels, lengths and contained exon numbers (Methods). A two tailed unpaired Welch’s t-test was calculated between groups of RNAs and the p-value displayed above the plot. **k**, Proportion of mRNAs (>4 exons) harboring detained or retained introns in PAXT-sensitive mRNAs compared to control mRNAs of the same expression levels, lengths and contained exon numbers (Methods). **l**, Aggregate plots displaying log_2_-scaled differences (note: not log_2_ fold changes, as exons and introns differ in their coverage ranges) of RNAseq coverage over retained introns, and their spanning exons, of PAXT-sensitive mRNAs (>4 exons) in NUC or CYTO fractions of depleted ZFC3H1 (left) or LENG8 (right) cells as compared to unperturbed HeLa cells.

In addition to decay, ZFC3H1 also contributes to nuclear retention of pA^+^ RNA^11,12,15^, and LENG8 has been suggested to have a similar capacity^32^. To analyse the effect of ZFC3H1 or LENG8 on transcript fate, we therefore performed nuclear/cytoplasmic pA^+^ RNA-seq obtained by fractionating HeLa cells before or after the rapid depletion of these factors (Extended Data Fig. 7a,b). Consistent with the RNA-retention capacities of ZFC3H1 and LENG8, PAXT-sensitive RNAs, defined by their increased abundance upon either ZFC3H1 or LENG8 depletion (Methods), displayed higher nuclear-to-cytoplasmic ratios in unperturbed HeLa cells than the remaining pA^+^ transcriptome (Fig. 3f). Clustering all transcripts by their depletion-dependent nuclear or cytoplasmic content changes demonstrated that more than half of the displayed RNAs were immune to ZFC3H1 or LENG8 depletion (Extended Data Fig. 7c, left, cluster 1, Supplementary Table 2 and Methods). Whereas most of these transcripts were accounted for by spliced mRNAs with multiple exons (Extended Data Fig. 7c, biotypes), a closer examination of the low-exon count RNAs from cluster 1 revealed mild, but detectable, sensitivity to ZFC3H1 depletion (Extended Data Fig. 7d). Outside of cluster 1, the remaining transcripts were, to variable extents, upregulated in both compartments upon ZFC3H1 and LENG8 depletions, demonstrating inefficient nuclear retention (Extended Data Fig. 7a, lanes 1–4 and 6–9 and Extended Data Fig. 7c, clusters 2–4). Repeating the same depletion experiments in HCT116 cells (Extended Data Fig. 7e,f) recapitulated these trends (Extended Data Fig. 7g). It therefore appears that PAXT generally retains and mediates decay of short pA^+^ RNAs with few exons, whereas transcripts that escape these fates are commonly longer and more exon-rich.

Although the protein-coding fraction of analysed transcripts was largely insensitive to PAXT, a minor subset of sensitive mRNAs was still detectable (Extended Data Fig. 7c, note biotypes of clusters 2–4). Similar to noncoding PAXT substrates, these were primarily low-exon (1–4 exons) transcripts (Fig. 3g), enriched in nuclei of unperturbed cells (Extended Data Fig. 7h). However, in the same condition, these short mRNAs were present at higher levels than their ncRNA counterparts (Extended Data Fig. 7i), suggesting that they might have acquired means to fend off nuclear turnover (see Discussion). DESeq2 also identified 105 longer, multi-exonic mRNA outliers (more than 4 exons), that were PAXT-sensitive despite their higher exon number and length. As general PAXT targets (Fig. 3f), these transcripts showed increased nuclear-to-cytoplasmic ratios in unperturbed cells compared with PAXT-insensitive controls (Extended Data Fig. 7j), suggesting that prolonged nuclear residence time may drive their sensitivity to PAXT. Notably, the LENG8 mRNA belongs to this transcript category, implying autoregulation of the PAXT pathway (see below). Since introns contribute to nuclear RNA retention^33–35^, we assessed PAXT-sensitive multi-exonic mRNAs (more than 4 exons) for reads spanning both 5′ and 3′ splice junctions (Methods). Indeed, when compared with a control population, these transcripts were enriched for retained introns, including previously described detained introns^36^ (Extended Data Fig. 7k). Moreover, upon ZFC3H1 or LENG8 depletion, PAXT-sensitive multi-exonic transcripts accumulated largely as incompletely spliced precursors in the nucleus and as fully spliced mRNAs in the cytoplasm (Extended Data Fig. 7l). Thus, upon PAXT impairment, this group of transcripts can be post-transcriptionally spliced and exported. Finally, we also identified an additional 3,209 PAXT-sensitive introns, including 161 detained introns (Supplementary Table 3), for which the corresponding spliced mRNAs were PAXT-insensitive as measured by DESeq2. We therefore propose that, in addition to short RNAs, PAXT can also target multi-exonic transcripts, which are retained in the nucleus due to incomplete splicing. In certain cases, this alters the cellular levels of the mature transcript, the extent of which might differ between cell types.

Together, these cellular data indicate that LENG8–PS constitutes a functional module of PAXT, which primarily targets nuclear pA^+^ RNAs with no, or only a few, introns as well as a minor pool of longer intron-containing transcripts. This overall retention–decay regime may be exploited for the regulation of selected mRNAs.

## PAXT and TREX-2 govern UAP56-bound RNP fate

**Fig. 4:**
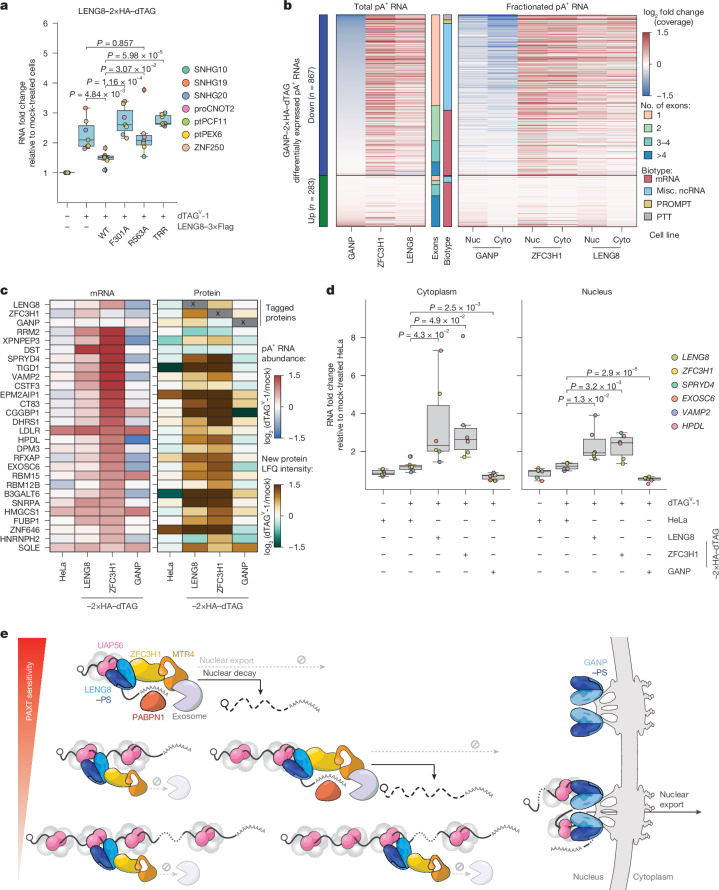
PAXT and TREX-2 compete for pA^+^ RNAs. **a**, Box plots of RT–qPCR analysis of selected PAXT substrates as in Fig. 3b, but for cells expressing wild-type LENG8, or LENG8(F301A), LENG8(R563A) or LENG8(TRR) constructs at endogenous levels (see Extended Data Fig. 8a). **b**, Heat maps as in Extended Data Fig. 7c, but of pA^+^ RNA log_2_ fold abundance changes in total (left) or nuclear (Nuc) and cytoplasmic (Cyto) fractions (right) of samples of dTAG^V^-1-treated (4 h) cells expressing GANP–2×HA–dTAG, ZFC3H1–2×HA–dTAG or LENG8–2×HA–dTAG relative to maternal HeLa cells. Transcripts were clustered and ranked on the basis of differential expression analysis of the total GANP depletion sample. Misc., miscellaneous. **c**, Heat maps of mean log_2_ fold changes of *n* = 3 biological replicates of PAXT-sensitive mRNAs (DESeq2; see Fig. 3) (left) and corresponding SILAC LFQ intensity changes (right) upon dTAG^V^-1 treatment of cells expressing LENG8–2×HA–dTAG, ZFC3H1–2×HA–dTAG or GANP–2×HA–dTAG. mRNA–protein pairs that were significantly (adjusted *P* value < 0.1) differentially expressed (per DESeq2 for mRNAs and DEP for proteins) are shown. Maternal HeLa cells are shown as controls. Heat maps were sorted by descending mRNA sensitivity to ZFC3H1 depletion. LENG8, ZFC3H1 and GANP depletion-induced mRNA and SILAC abundance changes are displayed in the top three rows. **d**, Box plots of RT–qPCR analysis as in Fig. 3a,b, but of selected short TREX-2 or PAXT substrates as well as the *LENG8* and *ZFC3H1* mRNAs in nuclear and cytoplasmic fractions of biochemically fractionated maternal HeLa cells or 2×HA–dTAG-tagged cell lines subjected to dTAG^V^-1-induced depletion of LENG8, ZFC3H1 or GANP for 4 h. RNA fold changes were calculated relative to levels in non-treated HeLa control cells and normalized to *GAPDH* mRNA separately for cytoplasmic and nuclear fractions. **e**, Model of nuclear pA^+^ RNA sorting.

**Extended Data Fig. 8:**
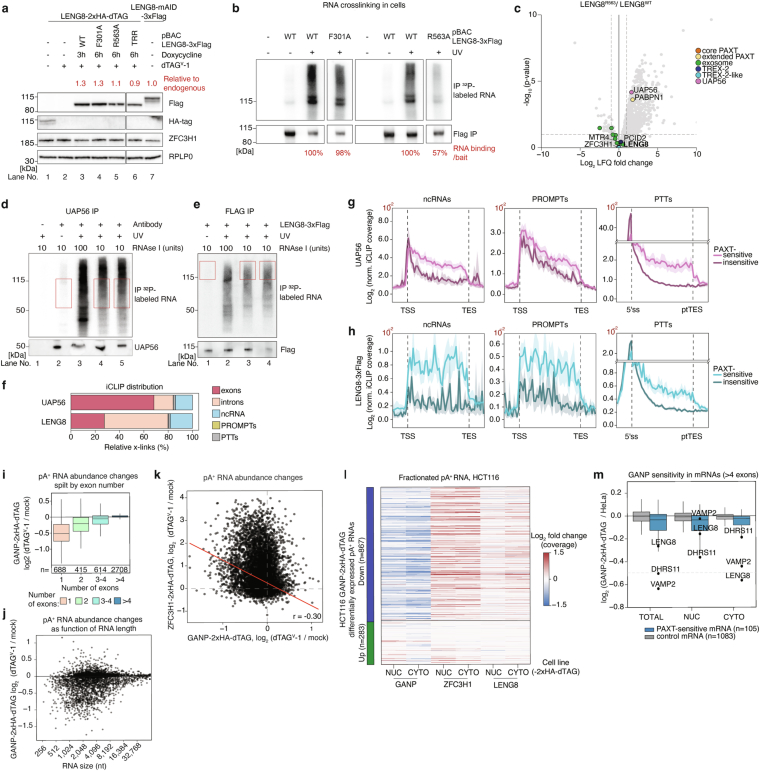
TREX-2 depletion results in downregulation of export-competent PAXT substrates. **a**, Western blotting analysis of WCEs of HeLa LENG8-2×HA-dTAG cells expressing, or not, the listed piggyBAC LENG8–3×Flag variant constructs for 3 or 6 h as indicated. Cells were treated with dTAG^V^-1 for 4 h. Membranes were probed with HA-tag, Flag-, ZFC3H1- and RPLP0- Abs. Lysate from an endogenously tagged LENG8–3×Flag cell line was loaded as a reference for LENG8 expression. **b**, Autoradiogram of RNA signals deriving from IPs of LENG8(WT), LENG8(F301A) or LENG8(R563A) constructs upon 150 mJ/cm^2^ UV crosslinking (+) or not (−). Western blotting analysis of the Flag IPs were probed with Flag Ab (‘Flag IP’). Quantified RNA- to Flag signals relative to the LENG8(WT) construct are shown in red. **c**, Volcano plot as in Fig. 2g, but for LENG8(R563A)–3×Flag relative to LENG8(WT)–3×Flag sample data. **d**, Autoradiogram as in **b**, but of RNA signals from IPs of endogenous UAP56. Red rectangles indicate areas extracted for library preparation. Western blotting analysis of UAP56 is shown below. **e**, Autoradiogram as in **b**, but of RNA signals from LENG8–3×Flag IPs. **f**, Relative distribution of iCLIP signals derived from **d** and **e** across the annotated HeLa transcriptome. The mean of two biological replicates is shown (see Methods). **g**. Aggregate plots of UAP56 mean (n = 2 of biological replicates) iCLIP signal coverage normalized to transcript expression levels as measured by total RNAseq (see Methods). Transcript biotypes are as in Extended Data Fig. 6g and further curated to exclude overlap with other TUs. PAXT-sensitive and -insensitive (control) transcripts for each biotype were defined as shown in Supplementary Fig. 5b (see Methods). **h**, Aggregate plots as in **g**, but for the LENG8–3×Flag iCLIP. **i**, Boxplots of pA^+^ RNA log_2_ fold abundance changes as in Fig. 3e, but for GANP–2×HA-dTAG cells. **j**, Scatterplots like in Extended Data Fig. 6o, but for GANP–2×HA-dTAG cells. **k**, Scatter plot as in Fig. 3c, but of pA^+^ RNA log2 fold abundance changes resulting from dTAG^V^-1 treatment of ZFC3H1– (y-axis) or GANP– (x-axis) 2×HA-dTAG cells. **l**, Heatmaps as in Fig. 4b, but of pA^+^ RNA log_2_ fold abundance changes in nuclear (NUC) or cytoplasmic (CYTO) fractions of dTAG^V^-1-treated (4 h) GANP–, ZFC3H1– or LENG8-2×HA-dTAG cells relative to their maternal HCT116 cells. **m**, Boxplots of total, nuclear and cytoplasmic pA^+^ RNA log_2_ fold abundance changes after dTAG^V^-1 treatment for 4 h of GANP–2×HA-dTAG cells of PAXT-sensitive multi-exonic (>4 exons) mRNAs compared to control group of multi-exonic mRNAs. PAXT-sensitive mRNAs with log_2_ fold changes below or above 0.5 in either of the compartments are shown.

Having defined LENG8–PS as a central physical and functional module of PAXT, we investigated how the ability of LENG8 to release UAP56 from RNA in vitro (Fig. 1c and Extended Data Fig. 1l) affects its function in vivo. Similar to the ZFC3H1-binding–defective LENG8(F301A) mutant, the LENG8(TRR) and LENG8(R563A) variants (Extended Data Fig. 8a)—which were compromised for UAP56 binding (Extended Data Fig. 1j,k) and UAP56 release from RNA (Fig. 1c), respectively, in vitro—were unable to suppress selected PAXT targets that were upregulated upon depletion of endogenous LENG8 (Fig. 4a). Notably, compared with the wild-type and F301A constructs, LENG8(R563A) displayed reduced RNA binding in vivo (Extended Data Fig. 8b), despite its ability to bind major interactors (Extended Data Fig. 8c and Supplementary Table 1). We therefore reasoned that release of RNA from UAP56 by LENG8–PS is central for PAXT-mediated turnover. To further pursue this notion, we conducted crosslinking followed by immunoprecipitation (iCLIP) experiments^37,38^ with endogenous UAP56 and endogenously 3×Flag-tagged LENG8 in HeLa cells (Extended Data Fig. 8d,e and Supplementary Table 4) and quantified the iCLIP coverages of these proteins, normalized to transcript abundance, across three distinct classes of PAXT targets: pA^+^ ncRNAs, PROMPTs and PTTs. Although the majority of the UAP56 iCLIP signal mapped to exons (Extended Data Fig. 8f), consistent with its established role in messenger ribonucleoprotein particle (mRNP) maturation and export^7,16^, both UAP56 and LENG8 exhibited selective enrichment over PAXT-sensitive transcripts compared with their expression-matched and exosome-sensitive control groups (Extended Data Fig. 8g,h, Supplementary Fig. 5a,b and Methods). Thus UAP56 binds a wide range of RNAPII transcripts, including PAXT substrates. Such broad RNP incorporation presumably occurs via UAP56 recruitment through diverse set of factors, many of which include UAP56 binding motifs (UBMs)^7,39,40^.

Together, the above experiments demonstrated the targeting of UAP56-bound RNPs by PAXT–LENG8–PS in vivo, suggesting that UAP56, while being an RNA export factor, is also crucial for nuclear RNA decay. To interrogate possible relations between these two fates of UAP56-bound RNPs, we first analysed the consequence of rapid GANP depletion in cells expressing GANP–2×HA–dTAG (Extended Data Fig. 6c) by pA^+^ RNA-seq (Extended Data Fig. 6d). In agreement with previous studies^26,41^, this mainly resulted in the downregulation of short pA^+^ RNAs with few introns (Fig. 4b and Extended Data Fig. 8i,j), resembling PAXT-sensitive transcripts (Fig. 3d and Extended Data Fig. 6o). Indeed, GANP-sensitive RNAs were largely upregulated upon depletion of ZFC3H1 or LENG8 (Fig. 4b, left and Extended Data Fig. 8k). We therefore propose that the suppressive effect of GANP reduction reflects PAXT-mediated degradation of export-restricted transcripts. Consistently, our fractionated pA^+^ RNA-seq data displayed the coinciding cytoplasmic accumulation of these RNAs following LENG8 or ZFC3H1 depletion (Fig. 4b, right). Equivalent depletion experiments in HCT116 cells (Extended Data Fig. 7e,f) recapitulated these trends (Extended Data Fig. 8l). By contrast, GANP reduction did not significantly affect levels of nuclear retained multi-exonic (more than 4 exons) PAXT substrates (Extended Data Fig. 8m; note the *LENG8* mRNA defying this trend, see also below).

**Extended Data Fig. 9:**
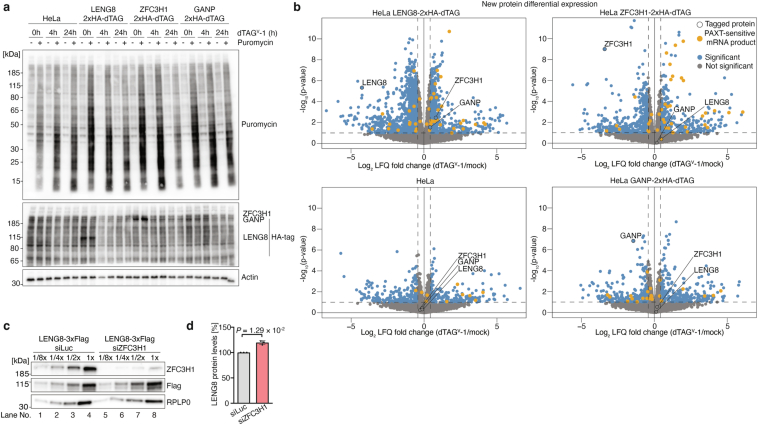
Nuclear pA^+^ RNAs are exported by TREX-2 if not retained and degraded by PAXT. **a**, Western blotting analysis of WCEs from maternal HeLa or ZFC3H1–, LENG8– and GANP–2×HA-dTAG cells mock treated or treated for 4 or 24 h with dTAG^V^-1. Cells were treated (+), or not (−), with 5 µg/ml of puromycin for 30 min before harvest. Membranes were probed with puromycin, HA-tag and actin -specific Abs as indicated. **b**, Scatter plots of differential expression analysis of new proteins synthesized during 24 h labelling in SILAC medium of LENG8–, ZFC3H1– or GANP–2×HA-dTAG cells and maternal HeLa cells treated or non-treated with 500 nM of dTAG^V^-1 for 28 h. Significantly up- (log_2_ fold change > 0.5) or down-regulated (log_2_ fold change < −0.5) proteins are shown in blue. Protein products of PAXT-sensitive mRNAs are shown in orange. LENG8, ZFC3H1 and GANP are highlighted. **c**, Western blotting analysis of serial dilutions (1/8x-1x) of WCEs from LENG8–3×Flag HeLa cells treated for 48 h with siRNA to luciferase (siLUC) or ZFC3H1 (siZFC3H1) as indicated. Membranes were probed with ZFC3H1-, Flag- and RPLP0-specific Abs as indicated. **d**, Quantification of LENG8–3×Flag signal from **c** normalized to RPLP0 levels. Three biological replicates were employed. A two-sided Student’s t-test was calculated between conditions and the p-value displayed above the barplot.

To further elaborate on an apparent competition of TREX-2 and PAXT for their target transcripts, we monitored protein production in TREX-2- or PAXT-perturbed cells. As expected, GANP depletion impaired global protein synthesis, as revealed by decreased puromycin incorporation (Extended Data Fig. 9a). Less intuitive, but consistent with prior reports on ZFC3H1^11,28^, depletion of LENG8 or ZFC3H1 also decreased synthesis of new protein, possibly owing to overloading of ribosomes by capped ncRNAs escaping from the nucleus^42^. We characterized these translational alterations further through the quantitative analysis of nascent protein synthesis using stable isotope labelling by amino acids in cell culture (SILAC) followed by mass spectrometry^43,44^ (Supplementary Table 5). Although only a subset of proteins matching PAXT-sensitive mRNAs were detectable, depletion of LENG8 or ZFC3H1 generally increased de novo peptide synthesis, whereas GANP depletion had little or opposing effects (Fig. 4c and Extended Data Fig. 9b). We note that this occurred despite the general conditions of decreased protein synthesis (Extended Data Fig. 9a). Proteins with increased synthesis upon LENG8 or ZFC3H1 depletion were enriched for RNA-binding proteins and RNA-processing regulators (Supplementary Table 5), which included the reciprocal increases of LENG8 and ZFC3H1 themselves (Fig. 4c and Extended Data Fig. 9b, top). Although only modestly increased in the SILAC experiment (Extended Data Fig. 9b, top right), LENG8 protein upregulation upon ZFC3H1 depletion was confirmed by western blotting analysis (Extended Data Fig. 9c,d). As the *ZFC3H1* mRNA similarly did not pass the DESeq2 analysis threshold (Supplementary Table 2), we used quantitative PCR with reverse transcription (RT–qPCR) to reveal that rapid depletion of LENG8 or ZFC3H1 led to the nuclear and cytoplasmic accumulation of mRNAs encoding these proteins, whereas GANP depletion decreased accumulation of *LENG8*, *ZFC3H1* and *GANP* (encoded by *MCM3AP*) mRNA (Fig. 4d). This supports the previous notion that retained mRNAs—such as *LENG8* mRNA—are subject to regulation by the PAXT pathway.

In conclusion, two structurally and functionally similar modules, LENG8–PS and GANP–PS, are critical interpreters of UAP56-bound nuclear pA^+^ RNPs. Nucleoplasmic PAXT and NPC-associated TREX-2 utilize these equivalent biochemical modules to control nuclear pA^+^ RNA homeostasis by facilitating decay and export, respectively.

## Discussion

Here we demonstrate that short and low exon content pA^+^ RNAs in UAP56-bound RNPs are highly susceptible to PAXT-mediated nuclear turnover. When PAXT function is impaired, NPC-associated TREX-2 seemingly grants export to these transcripts, demonstrating that LENG8–PS and GANP–PS can target similar pA^+^ RNPs. Based on these findings, we propose a general model for nuclear pA^+^ RNA fate determination (Fig. 4e). Both TREX-2 and PAXT may engage UAP56-bound pA^+^ RNPs. Owing to the nucleoplasmic localization of newly made pA^+^ RNPs, they would first encounter PAXT. However, being an adaptor of the 3′–5′ exonucleolytic exosome, PAXT targeting only translates into efficient decay if it occurs in the vicinity of the RNA 3′ end. This condition greatly sensitizes short transcripts. By contrast, for longer RNAs, which are compacted into larger RNPs with multiple UAP56s, a PAXT encounter may merely result in the release of an UAP56 molecule. This would counteract export indirectly, however, as long as a sufficient number of UAP56 molecules remains on the transcript, nuclear export via TREX-2 is still possible. In support of this model, a recent study found that the widespread interaction of ZFC3H1 with long and multiply spliced mRNAs did not affect transcript levels upon ZFC3H1 depletion^15^. We note that the targeting of LENG8–PS to decay-insensitive transcripts might have a role in maintaining sufficient levels of free nuclear UAP56, without which mRNA biogenesis defects, R-loop formation and genomic instability would prevail^45^. The importance of such UAP56 recycling might in fact explain why budding yeast, in which the PAXT complex has been lost, still harbours the LENG8–PS homologue^23^. Similarly, the cytoplasmic presence of a SAC3D1-containing TREX-2-like complex in higher eukaryotes might help remove residual UAP56 from RNA after export.

Although general, the proposed model may be bypassed by specific transcripts. For example, short functional pA^+^ mRNAs, including stress-induced transcripts, must overcome nuclear decay. How this is achieved remains an open area of research. Stress-induced mRNP reorganization might offer protection to the RNA 3′ end, or their robust transcriptional upregulation may ensure that, despite ample nucleoplasmic decay, a sufficient number of RNPs still reaches the NPC. Consistent with the latter notion, we find that PAXT-sensitive short mRNAs are expressed at higher levels than their ncRNA counterparts (Extended Data Fig. 7i). Finally, decay might also be short-circuited by gene gating, positioning a given locus in proximity to the NPC, as was recently demonstrated for *MYC*^46,47^. We also find a small subset of multi-exonic transcripts, that are sensitive to PAXT-mediated decay, violating the general nuclear pA^+^ RNA decay regime (Fig. 3g). We suggest that this sensitivity is enhanced by prolonged residence in the nucleus (Extended Data Fig. 7h,j), exacerbated by intron presence (Extended Data Fig. 7k). In line with nuclear retention of pre-mRNA being able to have a regulatory role^33–36^, our data provide evidence that the PAXT system itself is subject to such control (Fig. 4c). Thus the PAXT axis, which primarily targets short, non-functional pA^+^ RNAs, has been co-opted to regulate a subset of mRNAs that are sensitized either by low exon content or prolonged nuclear retention.

In summary, we propose that the major opposing fates of nuclear pA^+^ RNA—export and decay—exploit a shared molecular logic. At its centre is RNA-bound UAP56 and its highly regulated release from the pA^+^ RNP. This blurs previously held categorizations of proteins as specific RNA export or decay factors, and highlight the competition for common pA^+^ RNP features that ensures faithful gene expression.

## Methods

### DNA sequences

All oligonucleotide plasmid vectors are annotated in Supplementary Table 6.

### Purification of UAP56 and UAP56(Δ1–43)

His-tagged UAP56 constructs (10×His–3C–UAP56 or 10×His–3C–UAP56(Δ1–43), residues 44–428) were expressed in *Escherichia coli* BL21 DE3 RIL using autoinduction medium at 37 °C for 16 h. Following collection, cells were resuspended in lysis buffer (25 mM HEPES pH 7.9, 5% glycerol, 300 mM NaCl, 20 mM imidazole, 0.05% Tween-20, and protease inhibitors), disrupted via sonication, and clarified by centrifugation. The supernatant was sequentially filtered through 1-µm and 0.45-µm filters before affinity purification on a HisTrap HP 5 ml column (Cytiva), equilibrated in buffer A (25 mM HEPES pH 7.9, 5% glycerol, 300 mM NaCl, 20 mM imidazole). After washing with buffer A supplemented with 70 mM imidazole, bound proteins were eluted using a linear gradient of imidazole (70–200 mM in buffer A). Peak fractions were diluted in buffer B (25 mM HEPES pH 7.9, 5% glycerol, 1 mM DTT) to reduce the NaCl concentration to 100 mM and subsequently subjected to anion-exchange chromatography on a HiTrapQ 5 ml column (Cytiva), pre-equilibrated with buffer B. Elution was performed with a linear NaCl gradient (100–500 mM). Fractions containing UAP56 were concentrated and further purified via size-exclusion chromatography using a HiLoad 16/600 Superdex 200 pg column (Cytiva), equilibrated in buffer C (25 mM HEPES pH 7.9, 5% glycerol, 100 mM NaCl, 1 mM DTT). Peak fractions containing the purified protein were pooled, concentrated, flash-frozen, and stored at −80 °C.

### Purification of LENG8–PS^M^ and SAC3D1–PS^M^

Expression constructs encoding LENG8–PS^M^ (10×His–MBP–LENG8^491–800^, 3×V5–PCID2, SEM1), SAC3D1–PS^M^ (10×His–MBP–SAC3D1^48–404^, 3×V5–PCID2, SEM1), SAC3D1–PCID2-UAP56–UCM–N-UBM–SEM1 (10×His–MBP–SAC3D1^48–404^, 3×V5–PCID2–UAP56–UCM–N-UBM, SEM1), LENG8–PCID2–UAP56–N-UBM, SEM1 (10×His–MBP–LENG8^491–800^, 3×V5–PCID2–UAP56–N-UBM, SEM1) and their respective mutants were introduced into *E. coli* BL21 DE3 RIL (UCM is a UAP56-clamping motif). Cultures were grown in LB medium at 37 °C to OD600 ~1.0, at which point expression was induced with 0.5 mM IPTG, followed by overnight incubation at 18 °C. Cells were collected, lysed by sonication, and clarified by centrifugation. The supernatant was filtered (1 µm and 0.45 µm) and loaded onto a HisTrap HP 5 ml column equilibrated with buffer A, followed by washing and elution using a linear imidazole gradient up to 300 mM. Peak fractions were diluted to 50 mM NaCl in buffer B and subjected to anion-exchange purification on a HiTrapQ HP 5 ml column. After washing, complexes were eluted with a NaCl gradient (100–500 mM). Size-exclusion chromatography using a HiLoad 16/600 Superdex 200 pg column (Cytiva) in buffer C containing 250 mM NaCl yielded the final purified complex, which was concentrated, flash-frozen, and stored at −80 °C.

Recombinant EIF4A3 was purified as described previously^16^.

His-tagged DDX19 was expressed in *E. coli* BL21 DE3 RIL using LB medium, induced with 0.5 mM IPTG and expressed at 37 °C for 3 h. Following collection, cells were resuspended in lysis buffer (25 mM HEPES pH 7.9, 5% glycerol, 300 mM NaCl, 20 mM imidazole, and protease inhibitors), disrupted via sonication, and the lysate was cleared by centrifugation. The supernatant was sequentially filtered through 1-µm and 0.45-µm filters before affinity purification on a HisTrap HP 5 ml column (Cytiva), equilibrated in buffer A. The column was washed with buffer A containing 30 mM imidazole and bound proteins were eluted using a linear gradient of imidazole (50–300 mM). The peak fractions were incubated with 3C protease to cleave off the tag, and after 3C cleavage the peak fractions were diluted in buffer B to reduce the NaCl concentration to 50 mM, filtered through a 0.22-µm filter and next subjected to anion-exchange chromatography on a HiTrapQ 5 ml column (Cytiva), pre-equilibrated with buffer B supplemented with 50 mM NaCl. The column was washed with buffer B supplemented with 50 mM NaCl following sample loading. Elution was performed with a linear NaCl gradient (50–500 mM). Peak fractions containing DDX19 were concentrated and further purified via size-exclusion chromatography using a HiLoad 16/600 Superdex 200 pg column (Cytiva), equilibrated in buffer C. Peak fractions containing the purified protein were pooled, concentrated, flash-frozen, and stored at −80 °C.

### Analytical gel filtration

For each purified protein or complex an aliquot of 62.5 μg was loaded onto a Superdex 200 Increase 5/150 column (Cytiva), equilibrated in the respective gel filtration buffers. Peak fractions were analysed via SDS–PAGE (4–12% gradient) and visualized by Coomassie staining.

### UAP56–LENG8 and UAP56–SAC3D1– PS^M^ pulldown

MBP-tagged LENG8–PS^M^ or SAC3D1–PS^M^ was incubated with a fourfold molar excess of UAP56 or UAP56(Δ1–43) in buffer D (25 mM HEPES pH 7.9, 40 mM NaCl, 5% glycerol, 0.01% Igepal CA-630, 1 mM MgCl_2_, 1 mM TCEP), with or without 50 μM 15U RNA and 1 mM AMP-PNP. Reactions were mixed by rotation at 4 °C for 1 h before adding 30 μl of pre-equilibrated amylose resin (E8021S, NEB). After an additional 1-h incubation at 4 °C, unbound proteins were removed by centrifugation (1,500*g*, 2 min, 4 °C) and 3 washes with buffer D. Bound proteins were eluted by incubation at 4 °C for 1 h in buffer D supplemented with 100 mM maltose. Input and elution fractions were analysed via SDS–PAGE (4–12% gradient) and visualized by Coomassie staining.

### LENG8–ZFC3H1 pulldown

Magnetic Streptavidin beads (50 μl in-house produced slurry per reaction) were equilibrated in wash buffer (25 mM HEPES pH 7.9, 100 mM NaCl, 5% glycerol, 1 mM MgCl_2_, 1 mM TCEP, 0.01% Igepal CA-630). Wild-type or mutant ZFC3H1 peptide (200 µg) with an N-terminal biotin and a C-terminal fluorescein, were added to the beads in a 100 μl reaction volume and incubated on a rotating wheel at room temperature for 60 min. To remove excess peptide, beads were washed three times with wash buffer. Subsequently, 15 μg recombinant LENG8(283–346) or LENG8(283–346) F301A in a 100 μl reaction volume were added to the beads and the reaction incubated on a rotating wheel for 60 min at 4 °C. Following the incubation, beads were washed three times with wash buffer before bound proteins were eluted by incubating the beads for 5 min with 200 mM glycine pH 2.5. Elutions were neutralized with Tris pH 10.4 and separated by SDS–PAGE. To detect the fluorescently labelled peptides, gels were imaged in the Fluorescein channel on a Bio-Rad Chemidoc Imager prior to Coomassie staining to visualize the proteins.

### RNA unclamping assay

Biotinylated 15U RNA (33 µM) was mixed with recombinant UAP56 (10 µM) and 1 mM ATP in buffer E (20 mM HEPES pH 7.9, 40 mM KCl, 2 mM MgCl_2_, 5% glycerol, 0.1% Igepal CA-630). This mixture was incubated with 20 µl NeutrAvidin Agarose beads (29202, Thermo Scientific), pre-equilibrated in buffer E, for 30 min at room temperature. After washing to remove excess UAP56 and ATP, beads were resuspended in buffer E and aliquoted. LENG8–PS^M^ or SAC3D1–PS^M^ (2.2 µM or 0.44 µM) was added, followed by a 10-min incubation at room temperature. Unbound proteins were removed by sequential washes in high-salt buffer (buffer E with 500 mM KCl) and buffer E. RNA-bound proteins were eluted using 0.4 μg benzonase in buffer E for 10 min at room temperature, followed by SDS–PAGE analysis and quantification of remaining RNA-clamped UAP56 in Fiji.

### Grating-coupled interferometry

Grating coupled interferometry measurements were conducted using a Creoptix WAVE system (Creoptix) with 4PCP WAVEchips (quasi-planar polycarboxylate surface). Chips were conditioned in borate buffer (100 mM sodium borate pH 9.0, 1 M NaCl) before immobilization of a monoclonal anti-V5 antibody (R960252, Invitrogen; 2 μg ml^−1^ in 10 mM sodium acetate pH 5.0) via amine coupling. The surface was then passivated with 0.5% BSA (in 10 mM sodium acetate pH 5.0) and quenched with 1 M ethanolamine pH 8.0. V5-tagged LENG8–PS^M^ or SAC3D1–PS^M^ complexes were captured to the desired density. UAP56 was injected as a 1:2 dilution series, starting at 5 µM, with or without 200 µM 15U RNA, in 25 mM HEPES pH 7.9, 50 mM KCl, 1 mM MgCl_2_, 1 mM TCEP, with and without 1 mM ATP at 25 °C. Blank injections were used for double referencing, and a DMSO calibration curve corrected for bulk refractive index effects. Data were processed using Creoptix WAVEcontrol software, applying *x*/*y* offset correction, DMSO calibration, and double referencing. A one-to-one binding model was used for fitting, and results were plotted in R.

### ATPase assay

Steady-state ATPase activity of UAP56 was measured using an NADH-coupled enzymatic assay. Final reaction mixtures contained 5 U ml^−1^ rabbit muscle pyruvate kinase (Type III, Sigma-Aldrich), 5 U ml^−1^ rabbit muscle L-lactic dehydrogenase (Type XI, Sigma-Aldrich), 500 µM phosphoenolpyruvate, and 50 µM NADH. Reactions (10 µl) were assembled in 1,536-well plates using buffer F (25 mM HEPES pH 7.9, 40 mM KCl, 0.5 mM MgCl_2_, 5% glycerol, and 0.5 mM ATP), with either 2 µM UAP56 or 0.1 µM UAP56 in the presence of LENG8–PS^M^ or SAC3D1–PS^M^, and 100 µM 15U RNA when indicated. The decrease in NADH fluorescence emission was monitored at 37 °C using a PHERAstar FS plate reader (BMG LABTECH). A calibration curve from a NADH dilution series (0.03–100 µM) was used for quantification. ATPase activity was determined by linear regression of the NADH decay curves, corrected for ATP consumption, and expressed as ATP hydrolysis rates (molecules of ATP hydrolysed per second per enzyme). Reaction components were analysed by SDS–PAGE (4–12% gradient) and visualized using Coomassie staining.

### Cryo-EM sample preparation, imaging, and analysis

#### Cryo-EM sample preparation

For cryo-EM sample preparation we adopted a strategy previously used for UAP56–TREX-2^M^ (ref. ^7^): We fused UAP56 to PCID2 to optimize complex stochiometry and further fused UAP56 to a UCM and N-UBM. The latter was done to further promote the RNA-clamped conformation of UAP56 and more accurately mimic the native mRNP-bound state of UAP56, where N-UBM and UCM are present at high local concentrations to engage RNA-bound UAP56^2^. The N-UBM and UCM peptides are not observed in our cryo-EM structures and hence are not depicted or discussed in the main text. For cryo-EM grid preparation, LENG8–PCID2–UAP56–N-UBM–SEM1 (at 0.5 mg ml^−1^) or SAC3D1–PCID2–UAP56–UCM1–N-UBM–SEM1 (at 0.5 mg ml^−1^) were incubated in buffer G (25 mM HEPES pH 7.9, 5% glycerol, 1 mM MgCl_2_, 1 mM TCEP, 100 μM 15U RNA) with 1 mM AMP-PNP or 1 mM ATP on ice for 10 min. Cryo-EM grids were then prepared by applying 4 µl of the sample to glow-discharged Cu R1.2/1.3 200-mesh holey carbon grids (Quantifoil). Grids were blotted at 8 °C and 90% humidity and plunged into liquid ethane using a Leica EM GP2.

#### Cryo-EM data acquisition and processing of a UAP56–LENG8–PS^M^ complex AMP-RNP

Data collection was performed on a Titan Krios G4 electron microscope operating at 300 kV, equipped with a cold field emission gun, a Selectris energy filter (5 eV slit width, ThermoFisher), and a Falcon 4i direct electron detector (ThermoFisher). The objective aperture was retracted, and a 50 µm C2 aperture was used. A total of 5,405 micrographs were recorded using EPU software in .eer format, a pixel size of 0.575 Å per pixel, a total electron dose of 50 e^−^ Å^−2^, and defocus values ranging from −1 to −2.5 µm. On-the-fly preprocessing, including motion correction and contrast transfer function estimation, was performed using the CryoSPARC^48^ Live v113 workflow. Approximately 1.3 million particles were picked in WARP, extracted with a 400 Å box, binned to 1.8 Å per pixel, and subjected to 2D classification. Ab initio reconstructions of 45,345 particles selected from the 2D classification yielded a initial map for clamped UAP56 bound to LENG8–PS, which was further subjected to non-uniform refinement, from which 7,886 particles were selected by per-particle scale. These were then 3D refined in Relion 5.0 using BLUSH^49^, resulting in a 6.2 Å UAP56–RNA–LENG8–PS Map F.

#### Cryo-EM data acquisition and processing of LENG8–PS^M^ and UAP56-NTD– LENG8– PS^M^ complexes

Data were collected and pre-processed as outlined above. A total of 6,578 micrographs were recorded using EPU software in.eer format, a pixel size of 0.575 Å per pixel, a total electron dose of 50 e^−^ Å^−2^, and defocus values ranging from −1 to −2.5 µm. Approximately 1.5 million particles were picked in WARP, extracted with a 400 Å box, binned to 1.8 Å per pixel, and subjected to 2D classification, yielding 183.858 LENG8–PS particles. Ab inito reconstruction considering high-resolution frequencies resulted in an interpretable LENG8–PS cryo-EM map from 82,873 particles. These were then re-extracted with a 400 Å box, binned to 0.90 Å per pixel and subjected to a non-uniform refinement yielding the 3.5 Å resolution LENG8–PS Map D. Further 3D classification in Relion 5.0 revealed a subset of 4,824 particles with the UAP56 NTD bound, which refined to 4.86 Å (UAP56-NTD–LENG8–PS, Map E).

#### Cryo-EM data acquisition and processing of and a SAC3D1–PS^M^ a UAP56–SAC3D1–PS^M^ complex

We collected three datasets with the same microscope specifications and settings as for UAP56–LENG8–PS. Dataset 1 consists of 11,743 micrographs, dataset 2 consists of 6,006 micrographs collected at a tilt angle of 30° and dataset 3 contains 4,543 micrographs. We again performed on-the-fly preprocessing (patch motion correction and contrast transfer function estimation) using the CryoSPARC live routine before picking 4.5, 1.4 and 0.5 million particles (datasets 1, 2 and 3, respectively) in WARP. For processing in CryoSPARC, particles were extracted with a 400 Å box and binned to 1.8 Å per pixel. After 2D classification we obtained 276,000, 47,000 and 93,000 UAP56–SAC3D1–PS^M^ particles and conducted three rounds of heterogeneous refinement using ab initio models generated with the particles from dataset 1 (ref. ^50^). The resulting 129,495 particles were then re-extracted with a 400 Å box and binned to 0.90 Å per pixel and subjected to a non-uniform refinement yielding the 3.0 Å UAP56–SAC3D1–PS complex Map A. A further local refinement using a UAP56 mask resulted in the 2.6 Å UAP56–AMP-PNP–RNA Map B. The 2D-selected particles from dataset 3 (~93,000) were further subjected to ab initio reconstruction considering high-resolution information, yielding a readily interpretable cryo-EM map of a SAC3D1–PS^M^ complex. Re-extraction with a 400 Å box and binning to 0.90 Å pixel^-1^, non-uniform refinement and selection of particles per scale yielded 17,936 particles, which allowed for the refinement of a SAC3D1–PS^M^ complex cryo-EM map to 3.60 Å (Map C).

#### Model building

Structural modelling of all complexes began with Alphafold2 Multimer^51^ predictions of the respective complexes. The predicted models were docked into the respective maps and manually adjusted using COOT and ISOLDE in ChimeraX. Final refinements were performed in Phenix using the phenix.real_space_refine protocol, applying secondary structure and rotamer restraints to optimize fit and stereochemistry.

### HeLa cell culture and cell line generation

HeLa Kyoto or HCT116 cells were grown in Dulbecco’s modified Eagle’s medium (DMEM) supplemented with 10% fetal bovine serum (FBS) and 1% penicillin/streptomycin at 37 °C, 5% CO_2_. Transient transfections were performed using Lipofectamine 3000 (Invitrogen), according to the manufacturer’s instructions. CRISPR–Cas9 mediated genomic knock-ins using homology dependent repair donor vectors^25^ of C-terminal 3×Flag, 2×HA–FKBP12(F36V)–V(dTAG)^27^ in HeLa Kyoto and HCT116 cells was carried as described before^8^ with single guide RNAs (sgRNAs) and homology arms generated using primers listed in Supplementary Table 6 and cloned into tagging cassettes carrying Hygromycin or Neomycin resistance genes (plasmids listed in Supplementary Table 6). After transfection and antibiotic selection single cell clones were grown and tested by genomic PCR with primers flanking the insertion region, as well as with western blotting analysis. In the GANP–2×HA–dTAG cell line, we observed an additional band, which we interpreted as a truncated protein isoform localized to the cytoplasm. This isoform is produced from an RNA transcript that uses an early polyadenylation site appearing upstream of the tag insertion position. Since dTAG^V^-1 treatment led to rapid and substantial reduction of full-length GANP we opted to utilize this cell line.

To generate stably expressing LENG8–3×Flag and ZFC3H1–3×Flag constructs of wild-type and mutant variants, HeLa cells were transfected with pBAC vectors as described. Human LENG8 and ZFC3H1 cDNA constructs were cloned and inserted into piggyBAC (pBAC) vectors^52^ using NEBuilder HiFi DNA assembly (NEB). The LENG8 CDS was inserted into a doxycycline-inducible pBAC vector, harbouring a C-terminal 3×Flag tag and a puromycin selection marker. The ZFC3H1 CDS was inserted into a constitutively expressed pBAC vector, harbouring a C-terminal 3×Flag tag and a Blasticidin selection marker. Generated constructs are listed in Supplementary Table 6. LENG8- and ZFC3H1–2×HA–dTAG cells were transfected with the pBAC vectors along with a piggyBAC transposase expressing vector (pBASE) with Lipofectamine 3000. Cell pools were selected with puromycin or Blasticidin for 7–10 days until negative control cells died. For induction of expression of LENG8 pBAC constructs, cells were incubated for 24 h in culture medium supplemented with 1 mg ml^−1^ doxycycline (Sigma-Aldrich) before collection. Expression of the constructs was validated by western blotting analysis using antibodies against ZFC3H1 or Flag. Depletion of endogenous dTAG-tagged proteins was performed by the addition of dTAG^V^-1 to the culture medium for indicated time points at a concentration of 500 nM. Induction of expression of exogenous LENG8–Flag constructs was performed by adding 1 µg ml^−1^ doxycycline.

### Western blotting analysis of whole-cell extracts

Whole-cell protein lysates were prepared using lysis buffer (20 mM Tris-HCl, 0.5% NP-40, 150 mM NaCl, 1.5 mM MgCl_2_, 10 mM KCl, 10% glycerol, 0.5 mM EDTA, pH 7.9) freshly supplemented with protease inhibitors (Roche). Samples were clarified by centrifugation at 20,000 rcf for 10 min. Sample concentrations were adjusted after Bradford measurement and denatured by the addition of NuPage Loading Buffer (Invitrogen) and NuPage Sample Reducing Agent (Invitrogen) before boiling at 95 °C for 5 min. SDS–PAGE was carried out on NuPage 4%–12% Bis-Tris (Invitrogen) gels migrated in NuPage MOPS Running Buffer (Thermo) and transferred onto PVDF membranes in NuPage Transfer buffer (Thermo) at 4 °C, 15 V overnight. Western blotting analysis was carried out according to standard protocols with the antibodies listed in the Supplementary Table 6 and HRP-conjugated secondary antibodies (Dako). Bands were visualized by Super Signal West Femto chemiluminescent ECL (Thermo) and captured using an ImageQuant 800 imaging systems (GE Healthcare). The uncropped gel images with reference to panels in main and Extended Data figures are presented in Supplementary Fig. 1.

### Immunoprecipitation followed by western blotting analysis

Approximately 2 × 10^7^ cells per immunoprecipitation were extracted in lysis buffer (20 mM Tris-HCl, 0.5% NP-40, 150 mM NaCl, 1.5 mM MgCl_2_, 10 mM KCl, 10% glycerol, 0.5 mM EDTA, pH 7.9) freshly supplemented with protease inhibitors and cleared by centrifugation at 20,000 rcf for 20 min. Clarified lysates were incubated overnight at 4 °C with Flag antibody and Protein G Dynabeads (Thermo). Beads were washed three times with HT150 extraction buffer, transferring beads to a fresh tube on the final wash. For benzonase-treated immunoprecipitations, samples were resuspended in HT150 buffer freshly supplemented with protease inhibitors and 2 mM MgCl_2_ and split in two. One half of each sample was mock-treated and the other incubated with 500 units of benzonase for 20 min at 25 °C, 12,000 rpm. Samples were washed twice for 5 min at room temperature in 20 mM Tris-HCl pH 8 freshly supplemented with 2 mM CaCl_2_. Proteins were eluted by boiling in 1× NuPage loading buffer (Invitrogen) for 5 min. Supernatants were mixed with 10× Reducing Agent (Invitrogen) and denatured for a further 5 min at 95 °C before proceeding with western blotting analysis.

### Immunoprecipitations followed by mass spectrometry

All immunoprecipitations were performed label-free and in triplicates. GANP–3×Flag, PCID2–3×Flag, LENG8–mAID–3×Flag, and control HeLa Kyoto cells were collected as described above. Protein extractions were performed using material from 15 million cells per immunoprecipitation with 1 ml extraction buffer (20 mM Tris-HCl, 1% IGEPAL, 150 mM NaCl, 1.5 mM MgCl_2_, 10 mM KCl, 10% glycerol, 0.5 mM EDTA, pH 7.9) supplemented with 1× protease inhibitors cocktail (Roche). After brief sonication (3× 10 s, Amplitude 1, Branson Sonifier 250), the protein extracts were clarified by centrifugation (20,000 rcf for 10 min at 4 °C). Anti-Flag magnetic beads were prepared with anti-Flag M2 antibodies (Sigma F3165) conjugated to Dynabeads M-270 Epoxy (Invitrogen) as previously described^53^. Beads were washed three times with lysis buffer, for endogenous GANP–3×Flag, LENG8–3×Flag and PCID2–3×Flag immunoprecipitations, lysis buffer with additional NaCl to 450 mM final concentration was used (high stringency). For nuclease treatment beads were resuspended in 40 μl extraction buffer with 2 mM MgCl_2_, containing 1 μl Pierce Nuclease (for TREX-2 and TREX-2-like immunoprecipitations, Sigma E1014), Benzonase (for ZFC3H1 immunoprecipitations, Sigma) or as a control 1 μl of 1 mg ml^−1^ BSA (as indicated for the different experiments) and incubated with agitation at 25 °C for 20 min. Beads were washed with extraction buffer once and then proteins were eluted with SDS buffer (2% SDS, 100 mM Tris pH 6.8, 10% glycerol) at 25 °C, shaking for 5 min. Milder lysis and wash conditions using HT150 buffer (20 mM HEPES pH 7.4, 150 mM NaCl, 0.5% Triton X-100) were applied for 3×Flag immunoprecipitations of ZFC3H1—both endogenously and exogenously expressed—as well as for exogenously tagged LENG8–3×Flag. Mass spectrometry sample preparations were performed with the protein aggregation capture (PAC) procedure with proteolytic digestion on MagResyn HILIC beads using trypsin or chymotrypsin as indicated^12^. The peptides were purified and concentrated on C18 stage tips before subjected to liquid chromatography–mass spectrometry analysis with an Easy nanoLC system coupled directly to a Thermo Scientific Orbitrap Exploris 480 mass spectrometer. Mass spectrometry data were acquired by data dependent acquisition and searched against the UniProt protein sequence database using MaxQuant, with ‘match between runs’ and ‘label-free quantification’ enabled. The MaxQuant protein group output was analysed with the DEP package as previously described^44,54,55^.

### Chemical fractionation of HeLa cells

Chemical fractionation was performed using a protocol adapted from ref. ^56^. In brief, cells collected using trypsin digestion were first lysed using cytosol extraction buffer (0.15% NP-40, 10 mM Tris pH 7.4, 150 mM NaCl). Then nuclei were separated from cytoplasmic fractions using centrifugation, followed by nuclei washes using PBS solution and extraction of protein using lysis buffer extraction buffer (20 mM Tris-HCl, 1% IGEPAL, 150 mM NaCl, 1.5 mM MgCl_2_, 10 mM KCl, 10% glycerol, 0.5 mM EDTA, pH 7.9) or RNA using TRIzol reagent according to the manufacturer’s instructions.

### Immunofluorescence and colocalization analysis

Cells seeded on microscope coverslips were fixed with 4% paraformaldehyde in PBS for 20 min at room temperature, washed twice with PBS, and permeabilized with 0.1% Triton X-100 in PBS for 10 min at room temperature. Subsequently, cells were washed with PBS twice and blocked with 5% BSA in PBS-T for 1 h at room temperature. Coverslips were incubated for 1 h at room temperature with primary antibody dilution in 1% BSA, followed by three 5 min washes with PBS. Then, coverslips were incubated in a secondary antibody dilution with 1% BSA for 1 h at room temperature. Finally, cells were washed three times for 5 min with PBS, counterstained with DAPI and mounted onto glass slides using ProLong Gold Antifade Mountant. Images were acquired using a Zeiss LSM 980 confocal microscope equipped with Airyscan 2 under 40× or 63× oil-immersion Plan-Apochromat objectives. All images within the same experiment were taken with the same excitation power and exposure time and processed similarly using ZEN Blue 3.6 software. All antibodies and applied concentrations are listed in Supplementary Table 6. Pixel-based colocalization analyses were performed using the ZEN 3.6 (blue edition) colocalization module, with threshold setting based on the control background images and extracting the weighted colocalization coefficients for each image. For each cell line, the colocalization coefficient was calculated from six 40× images in two independent experiments, with at least 139 cells in total included in the analyses.

### RNA extraction and RT–qPCR

HeLa and LENG8–2×HA–dTAG, ZFC3H1–2×HA–dTAG and GANP–2×HA–dTAG cells were treated with 500 nM of dTAG^V^-1 or untreated for 4 h. RNA was extracted using TRIzol (Invitrogen) and treated with TURBO DNase (Invitrogen) according to the manufacturer’s protocol. To measure RNA levels, reverse transcription was carried out with SuperScript III reverse transcriptase (Invitrogen) using 1 µg RNA and a mixture of 20 pmol random hexamer in a 20 µl reaction at 50 °C according to the manufacturer’s protocol. Subsequently, quantitative PCR (qPCR) was performed using Platinum SYBR Green qPCR SuperMix-UDG (Invitrogen) in a ViiA 7 Real-Time PCR machine (Life Technologies with the primers listed in Supplementary Table 6). Relative quantities were calculated by normalizing samples to *GAPDH* mRNA levels. For pA^+^ RNA-seq, RNA was quality checked on an Agilent 2100 Bioanalyzer (Agilent Technologies) for integrity before shipping to the sequencing provider.

### pA^+^ RNA-seq library generations

All library construction and sequencing were paid services from the Beijing Genome Institute (BGI) in case of total pA^+^ RNA-seq and from Lexogen in case of the fractionated and exogenously expressing ZFC3H1 pA^+^ RNA-seq. Total RNA was extracted using TRIzol reagent according to the manufacturer’s instructions and transferred to BGI or Lexogen, which performed pA^+^ RNA selection using oligo-dT beads followed by strand-specific library preparation and sequencing.

### 3′ end-seq RNA library preparation

Triplicates of total 3′ end-seq libraries in presence or absence of EPAP, in EXOSC3–2×HA–dTAG cells, treated or not with dTAG^V^-1 for 4 h were generated and processed as before^57^. In brief, to discriminate pA^+^ from non-polyadenylated (pA^−^) RNA 3′ ends, 10 μg of RNA was split in two, subjecting one aliquot to in vitro polyadenylation by *E. coli* poly(A) polymerase (Invitrogen) in a 40 μl reaction at 30 °C (EPAP treated) according to the manufacturer’s protocol, while mock treating the other. Samples were then purified with the PureLink RNAmini kit (Invitrogen) and submitted for RNA 3′ end sequencing.

### Analysis of RNA-seq data

#### Annotation of pA^+^ PTTs

Polyadenylated PTTs, displaying sensitivity to ZFC3H1 and/or LENG8 depletion, were annotated using a custom pipeline. In brief, starting from our transcriptome annotation of HeLa cells^29^, transcription units were filtered to be longer or equal to 10 kb. At these transcription units, the pA^+^ RNA-seq coverage for the ZFC3H1–2×HA–dTAG and LENG8–2×HA–dTAG cell lines treated with DMSO or dTAG, was measured from TSS to transcription end site (TES) with a bin of 50 bp using rtracklayer^58^. Gene bodies were then scaled to 2 kb, replicates averaged, a pseudocount of 1 added and the log_2_ fold change (LFC) dTAG/Mock performed for each cell line. Using data from each cell line separately, transcription units were then filtered to display increased signal (LFC > 0.2) within the first 200 bp of the scaled gene body and no such difference in the last 200 bp (LFC < 0.2). Of note, the lenient LFC reflected the accumulation of a PTT overlapping with the full transcription unit, and the present criteria filtered out cases where both the full-length transcription unit and the PTT displayed sensitivity to the specific depletions. Following this, the PTT-harbouring transcription units, identified in ZFC3H1 and LENG8 depletions, were pooled. For each of these the maximum LFC value within the first 200 bp was defined and the last bin reaching 80% of this value along the scaled gene body was used to define an area to screen for the PTT TES. For each transcription unit, in the defined region ±5% of the transcription unit length, we measured, without binning or gene body scaling, the coverage from 3′ end RNA-seq, non-EPAP treated data from ZFC3H1-mAID cells mock or AID treated^28^. LFCs were measured as before and 3′ end peaks with ZFC3H1-sensitivity (LFC > 1) over the areas of interest were called. In each area, the strongest peak was considered as the PTT TES. A manual curation of the identified PTTs was then performed to filter out artifacts.

#### Annotation of pA^−^ PTTs

At first introns, the LFCs of normalized coverage at individual positions were calculated upon EXOSC3 depletion in EPAP- and non-EPAP-treated conditions (M.L.R. et al., unpublished observations; Supplementary Fig. 5a). To ensure annotation of unadenylated ends only, the LFC of the non-EPAP condition was subtracted from that of the EPAP condition. The most downstream position, displaying a residual LFC >0.2 was subsequently considered as the TES of the longest unadenylated PTT of the locus.

#### Processing of total and fractionated pA^+^ RNA-seq data

The raw sequence reads received from service providers were first quality checked using FastQC (https://www.bioinformatics.babraham.ac.uk/projects/fastqc/). Reads were then trimmed for adaptors and filtered using Trim Galore (https://www.bioinformatics.babraham.ac.uk/projects/trim_galore/). Trimmed reads were mapped to hg38, using Hisat2 in paired-end mode^59^. Mapped files were sorted and checked for pairing using SAMtools^60^. Reads were then deduplicated using MarkDuplicates (Picard; https://broadinstitute.github.io/picard/) and further filtered to keep only unique mappers by using SAMtools. Relative samples size was then estimated by generating coverage counts using htseq-count^61^ (HTSeq-counts) over the Gencode annotation to avoid any bias due to accumulation of short unstable transcripts present in our in-house annotation and then analysed by DESeq2^30^ to define size factors. In the case of fractionated pA^+^ RNA-seq, size factors were measured separately for the nuclear and cytoplasmic fractions to avoid any compensation of compartment specific phenotypes. Finally, reads were converted to bigwig files normalized to size factors using bamCoverage (Deeptools)^62^. The RNA sample of HeLa 4-h dTAG^V^-1 replicate 4 from the nuclear fraction appeared to suffer from a strong technical issue arising from large ribosomal RNA contamination. This replicate was therefore eliminated from all analyses but is still listed as part of the Gene Expression Omnibus (GEO) dataset.

#### Differential expression analysis

RNA sensitivities to LENG8, ZFC3H1 and GANP depletion were defined based on DESeq2 differential expression analysis of total pA^+^ RNA-seq using untreated cells as controls. For each depletion transcription units with adjusted *P* values < 0.1 in DESeq2^30^ analysis were considered as measurable and LFC over control >0.5 was counted as upregulated, while the LFC coverage over control < −0.5 was counted as downregulated. Owing to the strong correlation between coverage changes of LENG8 and ZFC3H1 depletion (Fig. 3c), ‘PAXT-sensitive’ transcription units were defined as upregulated in either of the two depletions. Plots exploring the relationship between exons or processed RNA lengths and PAXT sensitivities (Fig. 3e and Extended Data Figs. 6k and 8i,j) were based on our published in-house HeLa transcript annotation^29^ and LFC coverage for all transcription units with adjusted *P* values < 0.1 in DESeq2.

#### Nuclear to cytoplasmic ratios measurements

Nuclear to cytoplasmic ratios were calculated for each transcription unit using non-log transformed counts of nuclear and cytoplasmic pA^+^ RNA coverages. Zero value counts were filled with minimal values.

#### Transcription unit clustering based on fractionated pA^+^ RNA-seq behaviour

First the average LFC coverage, as measured by rtracklayers, was calculated for the total, nuclear and cytoplasmic fractions. For the fractionated sequencing, all proteins depletions were compared to the maternal HeLa cell line treated with dTAG^V^-1. The LFC upon ZFC3H1 depletion was then used separately in the nuclear and cytoplasmic fractions, at each transcription unit, to define a behaviour as ‘up’ (>0.5), ‘down’ (<−0.5), or ‘unaffected’. Nine clusters were then generated corresponding to all possible combinations (nuclear ‘up’/cytoplasmic ‘up’, nuclear ‘up’/cytoplasmic ‘unaffected’, etc.). Small clusters (with less than 200 transcription units) were removed from the final heat map in Extended Data Fig. 7c.

##### Analysis of transcripts with retained introns

For every intron unspliced reads spanning the 5′ and 3′ splice sites were counted using custom code relying on Samtools^60^, and every intron with at least one unspliced read at both junctions in unperturbed HeLa cells was considered as retained. The genomic coordinates of detained introns were obtained from Boutz et al.^36^. As this annotation originates from four combined cell lines, we first merged it with our in-house HeLa specific annotation^29^. Considering the generally unspliced nature of detained introns, we first filtered out these when overlapping totally or partially introns from our annotations. We then further filtered detained introns to be fully included in our exons to avoid overhang at the TSS or TES due to alternative isoforms. Finally, the few cases where a detained intron was starting or ending a transcription unit, without being preceded or followed by an exon, were filtered out. Similarly, reads spanning 5′ and 3′ splice junctions for both retained and detained introns were counted in total, nuclear and cytoplasmic fractions. Introns where dual splice junctions showed an increase upon ZFC3H1 or LENG8 depletion were counted as PAXT-sensitive.

##### Metagene profiles, heat maps and display of sequencing information

Metagene profiles and heat maps were produced using custom R and Python scripts. In brief, the rtracklayer R package was used to collect read coverage values for the window ±500 bp relative to the TSS or TES, or over specific exonic/intronic features. Coverage values were then binned in 50 nt bins and log_2_-transformed after the addition of a pseudocount of 1. This measurement of coverage was then used to compute LFC values and generate subsequent plots. Heat maps were made using custom R or Python code based on the R package ComplexHeatmap^63^ or Seaborn^64^, respectively. The mean of coverage values across transcription units over each bin were also computed and plotted as metagene profiles using custom R code. A 95% confidence interval of the mean coverage was displayed for each sample and was measured through 50 steps of bootstrap samplings with replacement. Aggregate plots and heat maps of sequencing data were generated based on BigWig files using customized R scripts. Genome browser views based on BigWig files were generated using the R package seqNdisplayR^65^.

### In vivo RNA-binding assays

Cells expressing LENG8–3×Flag of wild-type or R563A mutant variants were induced with doxycycline for 24 h and crosslinked with 150 mJ cm^−2^ of 254 nm UV lamp using Stratalinker2400 (Stratagene). Lysate preparations, anti-Flag immunoprecipitations, RNAse I (Thermo Scientific), TurboDNAse treatments (Thermo Scientific), radiolabelling using γ-^32^P ATP (PerkinElmer) and PAGE of RNA–protein complexes were performed as described^37^. The phosphor imaging of gels with radiolabelled samples was performed using a Typhoon scanner (Amersham).

### iCLIP experiments

iCLIP was performed as previously described^37^ with minor modifications. In brief, HeLa or HeLa LENG8–mAID–3×Flag cells at ~80% confluency were UV-crosslinked at 254 nm with a dose of 150 mJ cm^−2^ using a Stratalinker 2000. One (UAP56) or two (LENG8–3×Flag) 15-cm plates were used per immunoprecipitation and experiments were performed in duplicate. Whole-cell extracts were sonicated for 30 s and treated with TURBO DNase and RNase I prior to immunoprecipitation, using anti-UAP56 (E7W7M, Cell Signaling Technology) or anti-Flag M2 antibodies immobilized on Protein G Dynabeads. Protein–RNA complexes were subjected to high-salt washes, including freshly added 2 M urea in the wash buffer, and separated by PAGE. RNA was subsequently extracted and iCLIP libraries were constructed^38^ before sequencing on a NovaSeq platform (Lexogen).

Crosslink positions were mapped to the hg38 human genome using as previously described^66^. Counts were then generated over our in-house HeLa transcript annotation^29^ and normalized to transcript abundance through division by the respective transcripts mean log_2_(coverage) of average replicates of the no dTAG^V^-1 EXOSC3 control sample (M.L.R. et al., unpublished observations). To display iCLIP log2-transformed coverages across PAXT-sensitive or -insensitive transcript classes (ncRNAs, PROMPTs and PTTs), typical exosome targets were classified based on their sensitivity to ZFC3H1 depletion in pA^+^ RNA-seq using LFC (>0.5 for ZFC3H1-sensitive and <0.3 but >−0.3 for insensitive transcripts) and curated to remove overlapping transcripts. Comparisons were further restricted to monoexonic ncRNAs.

### Puromycin labelling assays

HeLa and LENG8–2×HA–dTAG, ZFC3H1–2×HA–dTAG and GANP–2×HA–dTAG-expressing cells were grown in the presence of 500 µM dTAG^V^-1 for additional 0, 4 and 24 h. Before collection by snap freezing, 5 µg ml^−1^ of puromycin was added to cell medium for 30 min. Puromycin incorporation was assessed by western blotting analysis.

### Whole-cell proteome analysis using pulsed SILAC

HeLa and LENG8–2×HA–dTAG, ZFC3H1–2×HA–dTAG and GANP–2×HA–dTAG cell lines were initially cultured in DMEM medium in the presence of 73 mg l^−1^ l-lysine HCl and 28 mg l^−1^ l-arginine HCl, (Sigma) (Lys0/Arg0 medium) for 24 h. Cells were then pre-treated with either 500 µM dTAG^V^-1 or an equivalent volume of DMSO for 4 h. Following this, the medium was switched to medium containing 73 mg l^−1^ l-lysine HCl and 28 mg l^−1^ l-arginine HCl l-lysine (^13^C_6_^15^N_2_) and l-arginine (^13^C_6_^15^N_4_), (for the Lys8/Arg10 medium) with either dTAG^V^-1 or DMSO, and cells were cultured for an additional 24 h under the same conditions. In parallel, a matched set of cells was maintained in Lys0/Arg0 medium under the same condition (dTAG^V^-1 or DMSO). After treatment, cells were collected by snap freezing and SILAC sample preparation and mass spectrometry were carried out as described^43^. SILAC ratios of Lys8/Arg10 versus Lys0/Arg0 peptides were calculated for each sample. To calculate differential protein expression, the DEP package^44^ was used to analyse mean LFQ intensities differences of Lys8/Arg10-labelled peptides.

### Statistics and reproducibility

In addition to the built-in statistical tests provided by software packages such as Zen Blue, DESeq2 and DEP, further statistical analyses were performed using two-sided *t*-tests or Welch’s *t*-test when group sizes differed substantially. Pearson correlation coefficients were used to assess the correlation between LFCs following ZFC3H1, LENG8 or GANP depletion.

Box plots show the median (centre line), interquartile range (box limits), and whiskers represent distribution of most extreme data points within 1.5× the interquartile range; *P* values of statistical tests are indicated directly on the plots. Outlier dots were excluded from the visual display for clarity but were included in the statistical analysis.

All real-time qPCR assays, RNA-seq, IP–MS and SILAC whole-cell proteomics were performed using three independent biological replicates, each comprising multiple technical measurements. Immunofluorescence staining (at least 100 cells were analysed per condition) and RNA-binding assays were repeated two times using independent batches of cells and all attempts of replication were successful. iCLIP libraries were prepared using two biological replicates. All other experiments, except cryo-EM data collection and processing, were performed at least three times with similar results and all attempts of replication were successful.

### Reporting summary

Further information on research design is available in the Nature Portfolio Reporting Summary linked to this article.

## Online content

Any methods, additional references, Nature Portfolio reporting summaries, source data, extended data, supplementary information, acknowledgements, peer review information; details of author contributions and competing interests; and statements of data and code availability are available at 10.1038/s41586-026-10650-0.

## Supplementary information

Supplementary Information Supplementary Figs. 1–5.

Reporting Summary

Supplementary Table 1 Results of immunoprecipitations followed by mass spectrometry.

Supplementary Table 2 Differential expression results.

Supplementary Table 3 Genomic coordinates of sensitive retained and detained introns.

Supplementary Table 4 Raw crosslink counts of iCLIP of UAP56 and LENG8.

Supplementary Table 5 SILAC whole-cell proteomics results.

Supplementary Table 6 Key resources table with vectors, primers, antibodies cell line information.

Supplementary Information Guide

Peer Review File

## Figures and Tables

**Extended Data Table 1:**
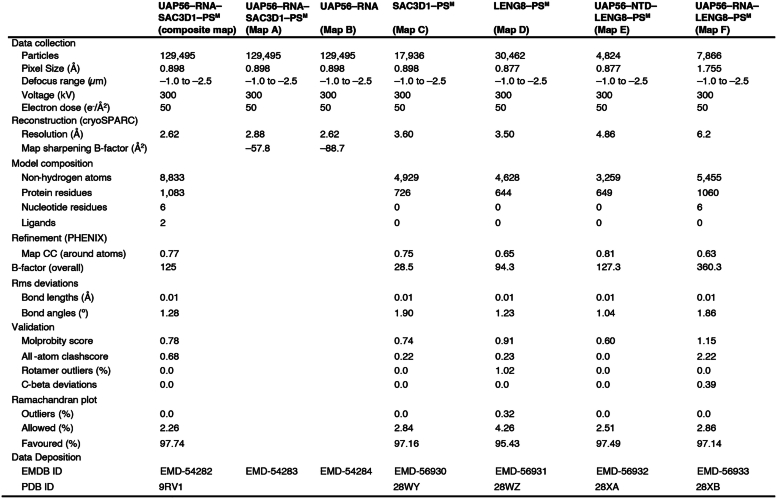
Cryo-EM data collection and refinement statistics

## Data Availability

Three-dimensional cryo-EM density maps of UAP56–RNA–SAC3D1–PS^M^ have been deposited to the Electron Microscopy Data Bank under the accession numbers EMD-54282 (composite map), EMD-54283 (Map A) and EMD-54284 (Map B). The coordinate file of UAP56–RNA–SAC3D1–PS^M^ has been deposited to the Protein Data Bank under the accession number 9RV1. Cryo-EM density maps of SAC3D1–PS^M^, LENG8–PS^M^, UAP56-NTD–LENG8–PS^M^ and UAP56–RNA–LENG8–PS^M^ have been deposited to the Electron Microscopy Data Bank under the accession numbers EMD-56930 (Map C), EMD-56931 (Map D), EMD-56932 (Map E) and EMD-56933 (Map F). The respective coordinate files of SAC3D1–PS^M^, LENG8–PS^M^, UAP56-NTD–LENG8–PS^M^ and UAP56–RNA–LENG8–PS^M^ have been deposited to the Protein Data Bank under the accession numbers 28WY, 28WZ, 28XA and 28XB. All newly generated RNA-seq data are available at GEO accession code GSE301785. All newly generated proteomics data are available at PRIDE accession code PXD076297. Raw immunofluorescence images have been deposited in the EMBL Bioimage Archive under accession code S-BIAD3166.
