# Supplementary material for: Molecular basis of polyadenylated RNA fate determination in the nucleus
Source: Nature. 2026 Jun 17;655(8124):1070–8. doi: 10.1038/s41586-026-10650-0 (PMC13391367; doi:10.1038/s41586-026-10650-0)
Supplement: Supplementary file 2 — Reporting Summary [file 41586_2026_10650_MOESM2_ESM.pdf]

nature portfolio

Corresponding author(s): Torben Heick Jensen

Last updated by author(s): Apr 15, 2026

Reporting Summary

Nature Portfolio wishes to improve the reproducibility of the work that we publish. This form provides structure for consistency and transparency in reporting. For further information on Nature Portfolio policies, see our Editorial Policies and the Editorial Policy Checklist.

Statistics

For all statistical analyses, confirm that the following items are present in the figure legend, table legend, main text, or Methods section.

n/a	Confirmed	
☐	☒	The exact sample size (*n*) for each experimental group/condition, given as a discrete number and unit of measurement
☐	☒	A statement on whether measurements were taken from distinct samples or whether the same sample was measured repeatedly
☐	☒	The statistical test(s) used AND whether they are one- or two-sided<br>*Only common tests should be described solely by name; describe more complex techniques in the Methods section.*
☒	☐	A description of all covariates tested
☐	☒	A description of any assumptions or corrections, such as tests of normality and adjustment for multiple comparisons
☐	☒	A full description of the statistical parameters including central tendency (e.g. means) or other basic estimates (e.g. regression coefficient) AND variation (e.g. standard deviation) or associated estimates of uncertainty (e.g. confidence intervals)
☐	☒	For null hypothesis testing, the test statistic (e.g. *F*, *t*, *r*) with confidence intervals, effect sizes, degrees of freedom and *P* value noted<br>*Give P values as exact values whenever suitable.*
☒	☐	For Bayesian analysis, information on the choice of priors and Markov chain Monte Carlo settings
☒	☐	For hierarchical and complex designs, identification of the appropriate level for tests and full reporting of outcomes
☒	☐	Estimates of effect sizes (e.g. Cohen's *d*, Pearson's *r*), indicating how they were calculated

Our web collection on statistics for biologists contains articles on many of the points above.

Software and code

Policy information about availability of computer code

Data collection	Grating Coupled Interferometry: Creoptix WAVE v3 software; Cryo-EM data were collected EPU 3;<br>Cryo-EM data acquisition and processing: Thermo Scientific EPU and CryoSPARC Live v113 software;<br>Coomassie stained gels: BioRad ChemiDoc MP Imaging System software (v2.4.0.03);<br>Immunofluorescence imaging: ZEN Blue v3.6;<br>Immunoblot-, gel- and phosphorimaging: Amersham™ ImageQuant™ 800 with control software v1.2.0, or Typhoon LA 9500 with control software v1.0;<br>Real-time qPCR: Agilent AriaMx software v2.1;<br>Mass spectrometry: Thermo Scientific Orbitrap Exploris 480 software and MaxQuant;<br>RNA sequencing data: Illumina platforms NextSeq 2000 or DNBSEQ-G400 software (data collected by external service provider, see Methods).
Data analysis	Grating Coupled Interferometry: Creoptix WAVE v3 software;<br>CryoEM data were analysed with WARP 1, RELION 5, cryoSPARC 4, Coot 0.9, Phenix 1.2, ISOLDE 1.6 and ChimeraX 1.7-1.11. ATPase assays were analysed with GraphPad Prism8 and R 4.0;<br>ATPase assays and RT-qPCR analysis: GraphPad Prism10 and Microsoft Excel v16.106.2;<br>Immunofluorescence imaging: ZEN Blue 3.6; Real-time qPCR: Agilent AriaMx software v2.1;<br>Mass spectrometry: MaxQuant, DEP package (Bioconductor, v1.28.0);<br>RNA sequencing data: FastQC v0.11.9, HT Seq v2.0.2, DESeq2 v1.42.1, deepTools v3.5.1, HISAT2 v2.2.1, SAMtools v1.13, Trim Galore v0.6.7, rtracklayer v1.54.0, Picard Toolkit v2.26.2;<br>Data visualization: seqNdisplayR v1.1.2, seaborn v0.13.2, plotly v5.24.1.

For manuscripts utilizing custom algorithms or software that are central to the research but not yet described in published literature, software must be made available to editors and reviewers. We strongly encourage code deposition in a community repository (e.g. GitHub). See the Nature Portfolio guidelines for submitting code & software for further information.

Data

Policy information about availability of data

All manuscripts must include a data availability statement. This statement should provide the following information, where applicable:

- Accession codes, unique identifiers, or web links for publicly available datasets
- A description of any restrictions on data availability
- For clinical datasets or third party data, please ensure that the statement adheres to our policy

Three-dimensional cryo-EM density maps of UAP56–RNA–SAC3D1–PSM have been deposited to the Electron Microscopy Data Bank under the accession numbers EMD-54282 (Composite Map), EMD-54283 (Map-A) and EMD-54284 (Map-B). The coordinate file of UAP56–RNA–SAC3D1–PSM has been deposited to the Protein Data Bank under the accession number 9RV1. Cryo-EM density maps of SAC3D1–PSM, LENG8–PSM, UAP56-NTD–LENG8–PSM and UAP56–RNA–LENG8–PSM have been deposited to the Electron Microscopy Data Bank under the accession numbers EMD-56930 (Map C), EMD-56931 (Map D), EMD-56932 (Map-E). and EMD-56933 (Map-F). The respective coordinate files of SAC3D1–PSM, LENG8–PSM, UAP56-NTD–LENG8–PSM and UAP56–RNA–LENG8–PSM have been deposited to the Protein Data Bank under the accession numbers 28WY, 28WZ, 28XA and 28XB.
All newly generated RNA-seq data are available at GEO accession code GSE301785.
All newly generated proteomics data are avaliable at PRIDE accession code PXD076297.
Raw immunofluorescence images have been deposited in the EMBL Bioimage Archive under accession code S-BIAD3166.

Research involving human participants, their data, or biological material

Policy information about studies with human participants or human data. See also policy information about sex, gender (identity/presentation), and sexual orientation and race, ethnicity and racism.

Reporting on sex and gender	N/A
Reporting on race, ethnicity, or other socially relevant groupings	N/A
Population characteristics	N/A
Recruitment	N/A
Ethics oversight	N/A

Note that full information on the approval of the study protocol must also be provided in the manuscript.

Field-specific reporting

Please select the one below that is the best fit for your research. If you are not sure, read the appropriate sections before making your selection.

☒ Life sciences ☐ Behavioural & social sciences ☐ Ecological, evolutionary & environmental sciences

For a reference copy of the document with all sections, see nature.com/documents/nr-reporting-summary-flat.pdf

Life sciences study design

All studies must disclose on these points even when the disclosure is negative.

Sample size	No sample size calculation was performed. Sample sizes were determined either by experimental constraints or by the number of transcription units or RNAs present in the human genome.
Data exclusions	Single RNA-seq sample (nuclear pA+ RNA of dTAGV-1-treated HeLa, replicate 2) was excluded from all analysis (while still provided at GEO, with remark) due to high ribosomal RNA contamination. Since all RNA-seq experiments were done in triplicates the read counts for two other replicate samples of the same experimental condition were used for downstream analyses.
Replication	All real-time qPCR assays, RNA-seq, immunoprecipitations followed by mass spectrometry, and SILAC whole-cell proteomics were performed using three independent biological replicates, each comprising multiple technical measurements.<br>Immunofluorescence staining (at least 100 cells were analyzed per condition) and RNA-binding assays were repeated two times using independent batches of cells and all attempts of replication were successful.<br>iCLIP libraries were prepared using two biological replicates.<br>All other experiments, except cryo-EM data collection and processing, were performed at least three times with similar results and all attempts of replication were successful.
Randomization	Experimental samples were assigned to groups based on the specific objectives and design of each experiment, rather than by random allocation. Appropriate control samples were included in every experiment to enable reliable interpretation of the results.

Blinding	Blinding was not considered relevant for this study, as the experimental outcomes were either binary or quantitatively measured, minimizing the potential for subjective interpretation.

Reporting for specific materials, systems and methods

We require information from authors about some types of materials, experimental systems and methods used in many studies. Here, indicate whether each material, system or method listed is relevant to your study. If you are not sure if a list item applies to your research, read the appropriate section before selecting a response.

Materials & experimental systems

n/a	Involved in the study	
☐	☒	Antibodies
☐	☒	Eukaryotic cell lines
☒	☐	Palaeontology and archaeology
☒	☐	Animals and other organisms
☒	☐	Clinical data
☒	☐	Dual use research of concern
☒	☐	Plants

Methods

n/a	Involved in the study	
☒	☐	ChIP-seq
☒	☐	Flow cytometry
☒	☐	MRI-based neuroimaging

Antibodies

Antibodies used	Primary antibodies:<br>FLAG-tag (M2) (Sigma, F3165), (β)-actin (Sigma, A2228), tubulin (Rockland, 600-401-880), EXOSC10 (Santa Cruz, sc-374595), EXOSC3 (Abcam, ab184961), GANP (Abcam, ab113295), HA-tag (Roche, 11867423001), PABPN1 (Abcam,ab75855), PCID2 (Laszlo Tora lab), MTR4 (Abcam, ab70552), vinculin (Sigma, V9131), ZFC3H1 (Sigma, HPA-007151), UAP56 (Cell Signaling, 47258), puromycin (Millipore, MABE343), RPLP0 (Abcam, ab192866).<br><br>Secondary antibodies:<br>Goat anti-rabbit-HRP (Dako, P0448); goat anti-mouse-HRP (Dako, P0447), goat anti-rat-HRP antibody (Dako, P0450), goat anti-mouse IgG Alexa Fluor™ 488 (Thermo Fisher, A11001), goat anti-rabbit IgG Alexa Fluor™ 594 (Thermo Fisher, A11012).<br><br>The dilutions for antibodies are listed in the Supplementary table 6 and referenced in Methods.
Validation	Puromycin antibody(Millipore, MABE343) is validated with human cells for western blotting analysis in the manufacture's manual;<br>RPLP0 (Abcam, ab192866) is validated with human cells for western blotting analysis in the manufacture's manual;<br>UAP56 (Cell Signaling, 47258) is validated with human cells for western blotting analysis in the manufacture's manual;<br>ZFC3H1 (Sigma, HPA-007151) is validated in this study using IP-WB in human HeLa Kyoto ZFC3H1-3xFLAG cell line using CRISPR knock-in of 3xFLAG sequence into terminal exon of ZFC3H1 gene: upon IP using anti-FLAG antibody a clear band of expected size is detectable in both whole cell extract and enriched in IP eluate (Extended Data Fig. 6a, lanes 2,8);<br>Vinculin (Sigma, V9131) is validated with human cells for western blotting analysis in the manufacture's manual;<br>MTR4 (Abcam, ab70552) is validated with human cells for western blotting analysis in the manufacture's manual;<br>PCID2 (gift from Laszlo Tora lab) is non-commercial antibody validated in this study using IP-WB in human HeLa Kyoto PCID2-3xFLAG cell line using CRISPR knock-in of 3xFLAG sequence into terminal exon of PCID2 gene: upon IP using anti-FLAG antibody a clear bands of expected sizes of endogenous and 3xFLAG-tagged proteins are detectable in both whole cell extract and enriched in IP eluate (Extended Data Fig. 6a, lanes 4,10);<br>PABPN1 (Abcam,ab75855) is validated with human cells for western blotting analysis in the manufacture's manual;<br>HA-tag (Roche, 11867423001) is validated with human cells for western blotting analysis in the manufacture's manual;<br>GANP (Abcam, ab113295) is validated with human cells for western blotting analysis in the manufacture's manual;<br>EXOSC3 (Abcam, ab184961) is validated with human cells for western blotting analysis in the manufacture's manual;<br>EXOSC10 (Santa Cruz, sc-374595) is validated with human cells for western blotting analysis in the manufacture's manual;<br>Tubulin (Rockland, 600-401-880) is validated with human cells for western blotting analysis in the manufacture's manual;<br>(β)-actin (ACTB) (Sigma, A2228) is validated with human cells for western blotting analysis in the manufacture's manual;<br>FLAG-tag (M2) (Sigma, F3165) is validated with human cells for western blotting analysis in the manufacture's manual.

Eukaryotic cell lines

Policy information about cell lines and Sex and Gender in Research

Cell line source(s)	HeLa Kyoto cell line (RRID:CVCL_1922, source: the Anthony Hyman Laboratory, DOI:10.1016/j.cell.2015.09.053) or HCT116 CMV-OsTIR1 (RRID:CVCL_RJ11, source: the Edouard Bertrand Laboratory, Université de Montpellier) were used as control and maternal cell line for genomic modifications.<br><br>All other cell lines used in the study was produced and validated in-house from maternal HeLa or HCT116:<br>HeLa Kyoto GANP-3xFLAG<br>HeLa Kyoto PCID2-3xFLAG<br>HeLa Kyoto LENG8-mAID-3xFLAG<br>HeLa Kyoto SAC3D1-3xFLAG

	HeLa Kyoto ZFC3H1-3xFLAG<br>HeLa Kyoto GANP-2xHA-dTAG<br>HeLa Kyoto LENG8-2xHA-dTAG<br>HeLa Kyoto ZFC3H1-2xHA-dTAG<br>HeLa Kyoto LENG8-2xHA-dTAG/pBAC LENG8-3xFLAG WT<br>HeLa Kyoto LENG8-2xHA-dTAG/pBAC LENG8-3xFLAG R563A<br>HeLa Kyoto LENG8-2xHA-dTAG/pBAC LENG8-3xFLAG F301A<br>HeLa Kyoto LENG8-2xHA-dTAG/pBAC LENG8-3xFLAG TRR<br>HeLa Kyoto ZFC3H1-2xHA-dTAG/ pBAC ZFC3H1-3xFLAG WT<br>HeLa Kyoto ZFC3H1-2xHA-dTAG/ pBAC ZFC3H1-3xFLAG delta aa 730-747<br>HCT116 CMV-OsTIR1 GANP-2xHA-dTAG<br>HCT116 CMV-OsTIR1 LENG8-2xHA-dTAG<br>HCT116 CMV-OsTIR1 ZFC3H1-2xHA-dTAG
Authentication	Maternal HeLa Kyoto and HCT116 CMV-OsTIR1 were showing expected morphology and growth patterns. HCT116 CMV-OsTIR1 show expected resistance to puromycin (DOI:10.1016/j.celrep.2016.03.001).<br>No additional authentication was performed for maternal cell lines.
Mycoplasma contamination	The cell lines used in the study were tested negative for Mycoplasma contamination.
Commonly misidentified lines<br>(See ICLAC register)	No commonly misidentified cell lines were used.

Plants

Seed stocks	N/A
Novel plant genotypes	N/A
Authentication	N/A
