# Supplementary material for: Molecular basis of polyadenylated RNA fate determination in the nucleus
Source: Nature. 2026 Jun 17;655(8124):1070–8. doi: 10.1038/s41586-026-10650-0 (PMC13391367; doi:10.1038/s41586-026-10650-0)
Supplement: Supplementary file 9 — Supplementary Information Guide [file 41586_2026_10650_MOESM9_ESM.docx]

**SI Guide**

**Molecular basis of polyadenylated RNA fate determination in the nucleus**

Andrii Bugai^1*^, Ulrich Hohmann^2,3,#,*^, Ana Lorenzo^1*^, Max Graf ^2,4*^, Laura Fin^2^, Jérôme O. Rouvière^1^, Laszlo Tirian^3^, Yuhui Dou^1,5^, Marion Le Rest^1^, Patrik Polák^1^, Dennis Johnsen^1^, Lis Jakobsen^6^, Jens Skorstengaard Andersen^6^, Julius Brennecke^3**^, Clemens Plaschka^2**^, Torben Heick Jensen^1**^

*These authors contributed equally to this work. **Correspondence

Authors affiliations:

^1^Department of Molecular Biology and Genetics, Aarhus University, Universitetsbyen 81, 8000 Aarhus, Denmark.

^2^Research Institute of Molecular Pathology (IMP), Vienna BioCenter (VBC), 1030 Vienna, Austria. ^3^Institute of Molecular Biotechnology of the Austrian Academy of Sciences (IMBA), Vienna BioCenter (VBC), 1030 Vienna, Austria.

^4^Vienna BioCenter PhD Program, Doctoral School of the University of Vienna and Medical University of Vienna, 1030 Vienna, Austria.

^5^Friedrich Miescher Institute for Biomedical Research, 4058 Basel, Switzerland.

^6^Department of Biochemistry and Molecular Biology, University of Southern Denmark, Campusvej 55, 5230 Odense M, Denmark.

^#^Current address: Institute of Molecular Biology (IMB) gGmbH, 55128 Mainz, Germany.

Correspondence to be addressed: thj@mbg.au.dk, julius.brennecke@imba.oeaw.ac.at, clemens.plaschka@imp.ac.at

**Table of content:**

**Supplementary Fig. 1 | Uncropped gel images**

Uncropped gel images are presented in the order of their appearance in the manuscript. Subtitles indicate the corresponding figure and panel numbers. Antibody names are shown in the left and right corners of each image. Chemiluminescent signals are aligned with the corresponding colorimetric images of the protein size marker. The cropped areas appearing in Extended Data presented as dashed rectangles.

**Supplementary Fig. 2 | Size-exclusion chromatography of recombinant proteins**

Size exclusion chromatograms of recombinant proteins (UV280 traces, in shades of grey, left), with Coomassie-stained SDS-PAGE gels depicting indicated peak fraction shown alongside (right). Shown are **a**, LENG8–PS^M^, LENG8 TRR–PS^M^, LENG8 R563A–PS^M^; **b**, SAC3D1–PS^M^, SAC3D1 R102A–PS^M^; **c**, UAP56, UAP56^Δ1-43^; **d**, EIF4A3 and DDX19; **e**, LENG8 283-346, LENG8 283-346 F301A.

**Supplementary Fig. 3 | SAC3D1–PS and UAP56–RNA–SAC3D1–PS cryo-EM analysis**

**a.** SAC3D1–PCID2–SEM1 cryo-EM density (Map C), colored by subunit identity. SEM1, blue; PCID2, dark blue; SAC3D1 48-404, green blue. **b.** SAC3D1–PCID2–SEM1 cryo-EM density (Map C) as in **a**, bottom and top views. **c**. SAC3D1–PCID2–SEM1 cryo-EM density (Map C) colored by local resolution. **d**. UAP56–RNA–SAC3D1–PCID2–SEM1 cryo-EM density (Map A), colored by subunit identity. SEM1, blue; PCID2, dark blue; SAC3D1 48-404, green blue; UAP56, shades of pink; RNA, black. **e.** UAP56–RNA–SAC3D1–PCID2–SEM1 cryo-EM density (Map A) as in **d**, bottom and top views. **f.** UAP56–RNA–SAC3D1–PS (Map A), and UAP56–AMP-PNP–RNA (Map B) cryo-EM maps colored by local resolution. **g.** Representative segments from Map A (SAC3D1, PCID2, SEM1, UAP56 NTD) and Map B (UAP56, RNA, AMP-PNP) in cartoon representation with side chains shown as sticks and superimposed on the respective cryo-EM densities.

**Supplementary Fig. 4 | LENG8–PS, UAP56-NTD–LENG8–PS and UAP56–RNA–LENG8–PS cryo-EM analysis**

**a.** LENG8–PCID2–SEM1 cryo-EM density (Map D), colored by subunit identity. SEM1, blue; PCID2, dark blue; LENG8 491-800, green blue; UAP56, shades of pink. **b.** LENG8–PCID2–SEM1 cryo-EM density (Map D) as in **a**, bottom and top views. **c.** LENG8–PCID2–SEM1 cryo-EM density (Map D) colored by local resolution. **d.** UAP56–NTD–LENG8–PCID2–SEM1 cryo-EM density (Map E), colored by subunit identity, and with a superposition of the cartoon model (colored by subunit identity) and the map (transparent, grey) shown alongside. SEM1, blue; PCID2, dark blue; LENG8 491-800, green blue; UAP56, shades of pink. **e.** UAP56–NTD–LENG8–PCID2–SEM1 (Map E) cryo-EM maps colored by local resolution. **f.** UAP56–RNA–LENG8–PCID2–SEM1 cryo-EM density (Map F), colored by subunit identity, and with a superposition of the cartoon model (colored by subunit identity) and the map (transparent, grey) shown alongside. SEM1, blue; PCID2, dark blue; LENG8 491-800, green blue; UAP56, shades of pink; RNA, black. **g.** UAP56–RNA–LENG8–PCID2–SEM1 (Map F) cryo-EM maps colored by local resolution.

**Supplementary Fig. 5 | PAXT-sensitive and non-sensitive non-coding RNAs**

**a.** Principal component analysis as in Extended Data Fig. 6d but for 3’-end RNAseq upon rapid depletion of EXOSC3 in HeLa EXOSC3-2xHA-dTAG cells (Le Rest et al. unpublished results). **b-d.** Sensitivities to EXOSC3- **b**, ZFC3H1- **c**, or LENG8- **d** depletions of the curated ‘PAXT-sensitive’ and ‘non-sensitive’ RNA populations used for iCLIP data representations in Extended Data Fig. 8g,h. Aggregate plots show log_2_ coverages and log_2_-transformed coverage changes of RNAseq data following rapid EXOSC3 depletion (Le Rest et al. unpublished results), and pA^+^ RNAseq following rapid depletions of ZFC3H1 or LENG8 (this study). From left to right: Exosome substrates are monoexonic ncRNAs, PROMPTs and PTTs, each subdivided into PAXT-sensitive and -insensitive (control) groups as described in Methods.

**Supplementary Data Tables, supplied as separate files:**

**Supplementary Table 1 | Results of immunoprecipitations followed by mass spectrometry**

**Supplementary Table 2 | Differential expression results**

**Supplementary Table 3 | Genomic coordinates of sensitive retained and detained introns**

**Supplementary Table 4 | Raw crosslink counts of iCLIP of UAP56 and LENG8**

**Supplementary Table 5 | SILAC whole cell proteomics results**

**Supplementary Table 6 | Key resources table with vectors, primers, antibodies cell line information**
