# Supplementary material for: Molecular basis of polyadenylated RNA fate determination in the nucleus
Source: Nature. 2026 Jun 17;655(8124):1070–8. doi: 10.1038/s41586-026-10650-0 (PMC13391367; doi:10.1038/s41586-026-10650-0)
Supplement: Supplementary file 1 — Supplementary Figs. 1–5. [file 41586_2026_10650_MOESM1_ESM.pdf]

nature portfolio
https://doi.org/10.1038/s41586-026-10650-0

Supplementary information

Molecular basis of polyadenylated RNA fate determination in the nucleus

In the format provided by the authors and unedited

Extended Data Fig. 4a

ab: ZFC3H1

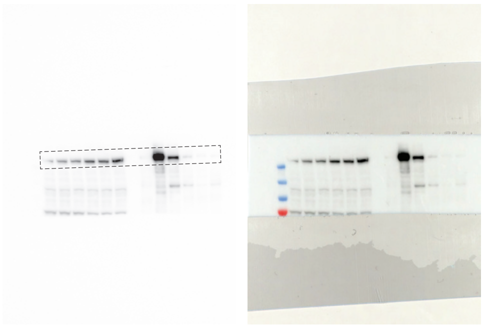

ab: GANP

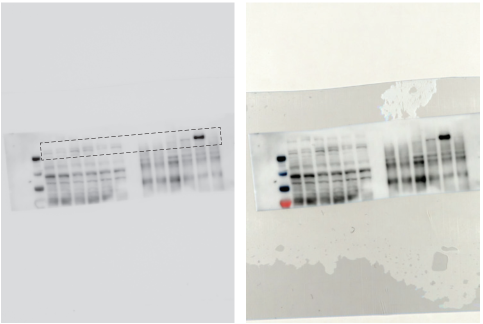

ab: EXOSC10

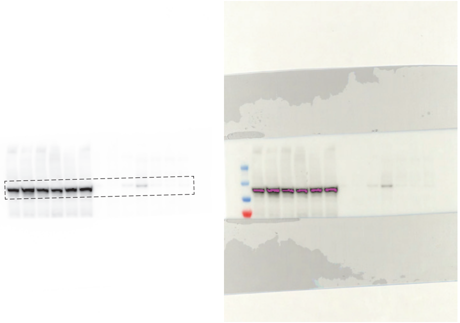

ab: FLAG

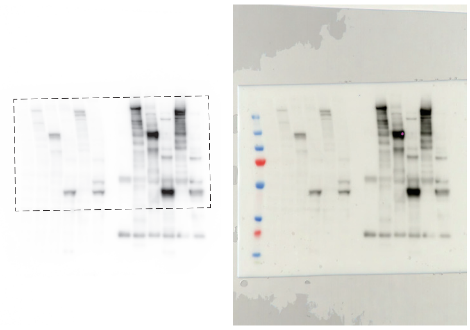

ab: MTR4

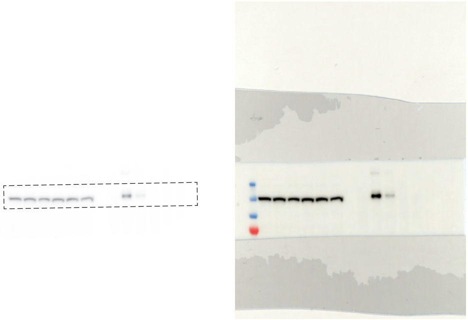

ab: PCID2

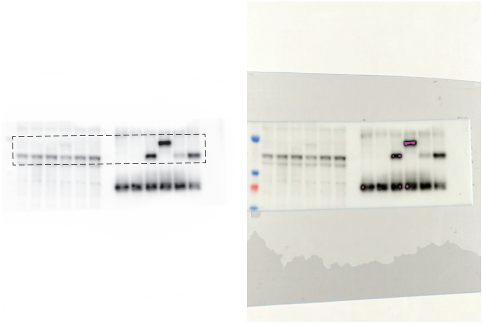

Extended Data Fig. 4g

ab: HA-tag

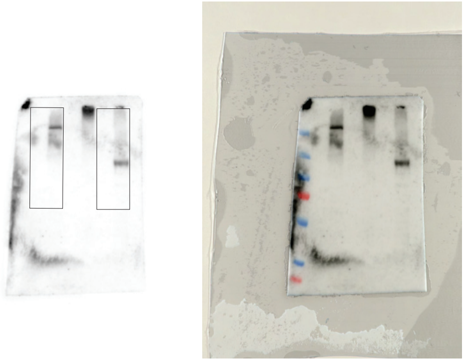

ab: Vinculin

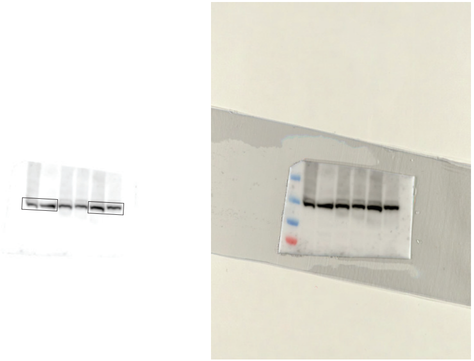

Extended Data Fig. 5a

ab: PCID2

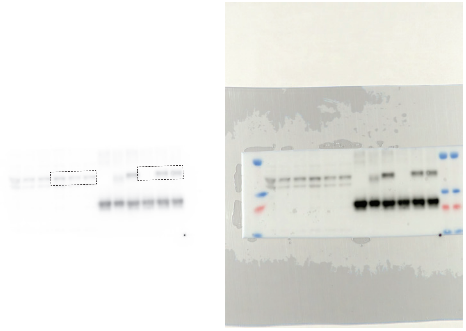

ab: EXOSC10

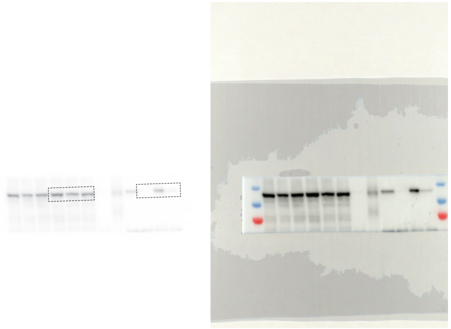

ab: MTR4

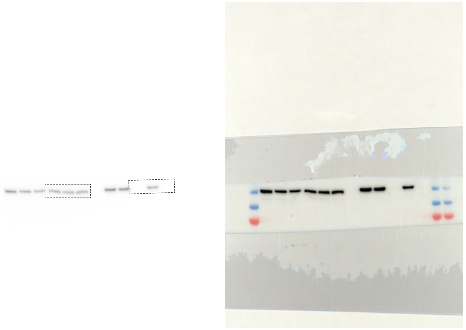

ab: ZFC3H1

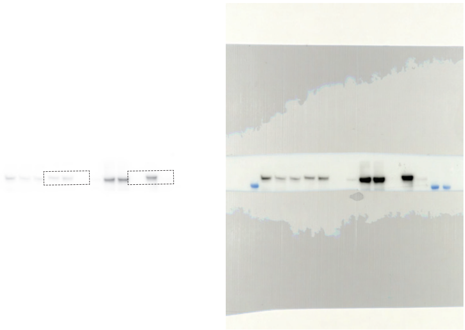

ab: FLAG

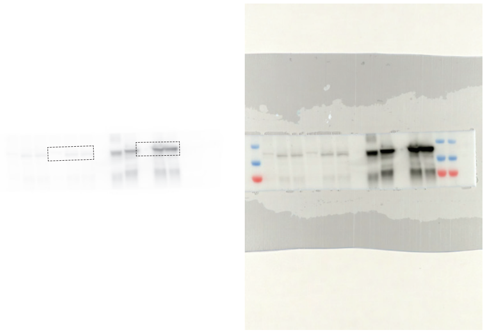

Size marker: PageRuler™ Plus Prestained Protein Ladder (Thermo Fisher)

Extended Data Fig. 5g

ab: ZFC3H1 (WCE)

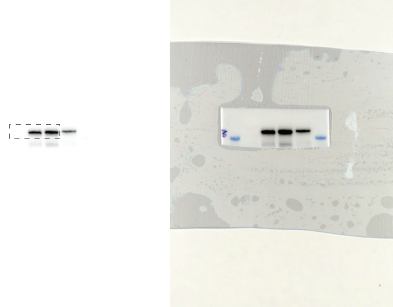

ab: ZFC3H1 (IP)

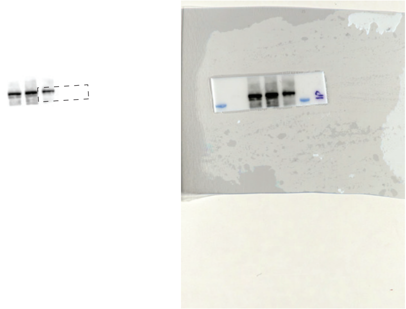

ab: FLAG (WCE)

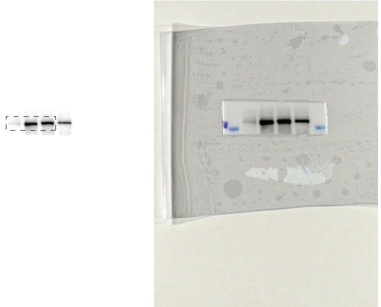

ab: MTR4 (WCE)

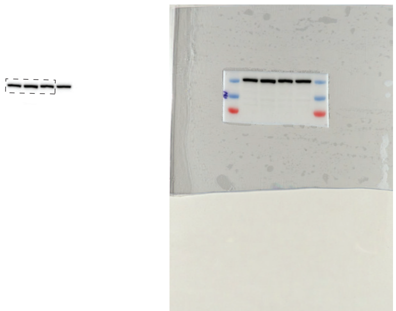

ab: MTR4 (IP)

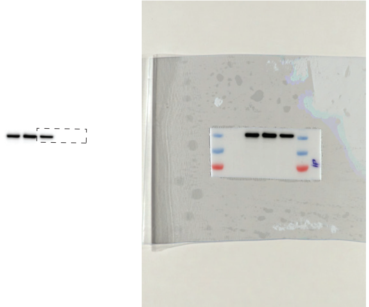

ab: FLAG (IP)

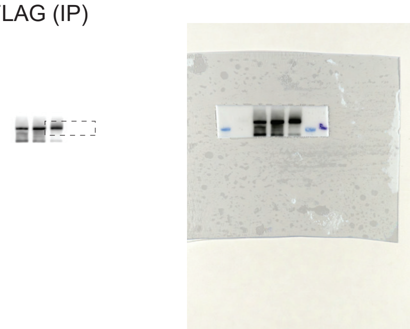

ab: EXOSC10 (WCE)

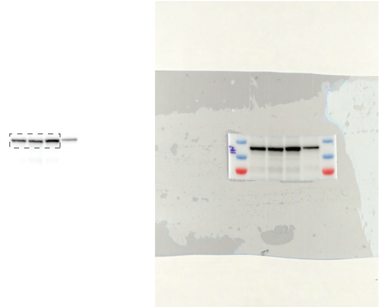

ab: EXOSC10 (IP)

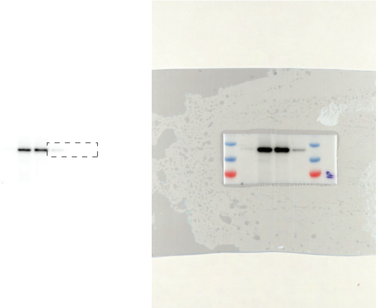

ab: Vinculin (WCE and IP)

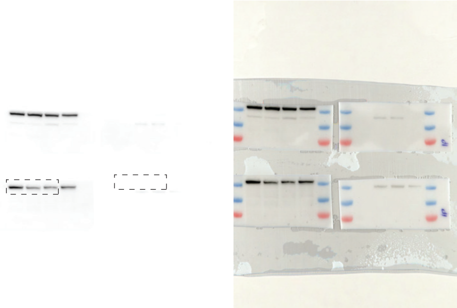

ab: PCID2 (WCE)

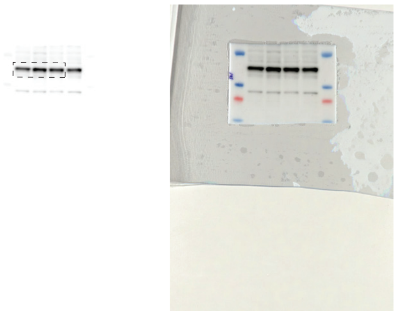

ab: PCID2 (IP)

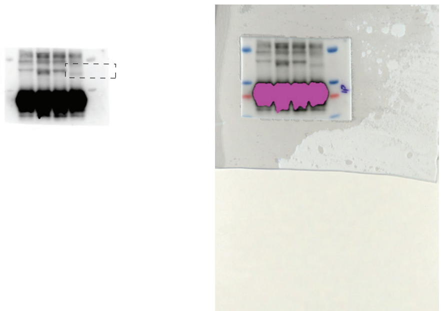

Extended Data Fig. 5i

Size marker (for Extended Data Fig. 7i):
GeneRuler 1 kb DNA Ladder (Thermo Fisher)

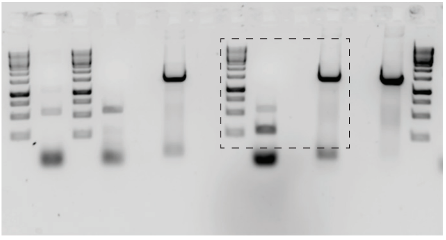

Extended Data Fig. 5j

ab: HA-tag

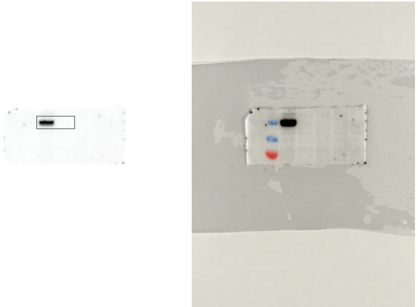

ab: ZFC3H1

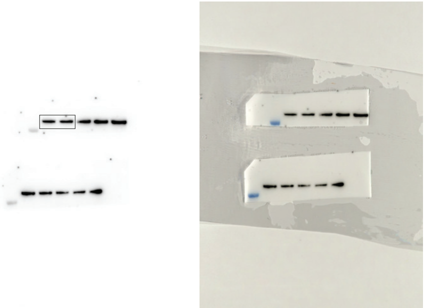

ab: ACTB

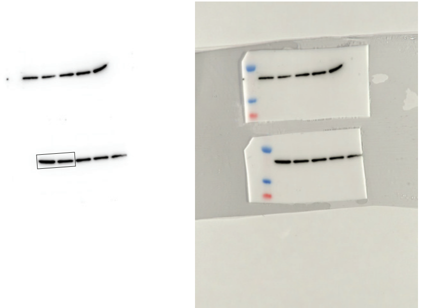

Size marker (except for Extended Data Fig. 7i): PageRuler™ Plus Prestained Protein Ladder (Thermo Fisher)

Extended Data Fig. 5k

ab: ZFC3H1

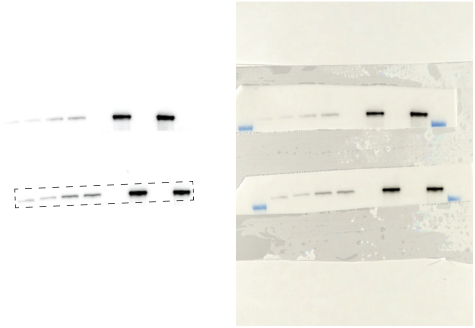

ab: MTR4

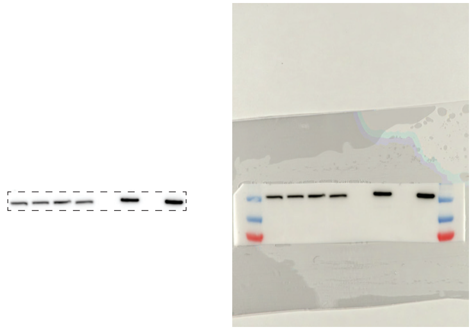

ab: ACTB

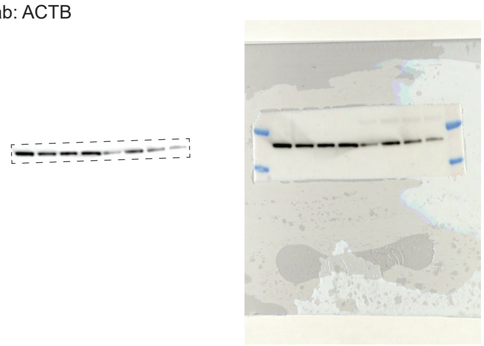

ab: FLAG

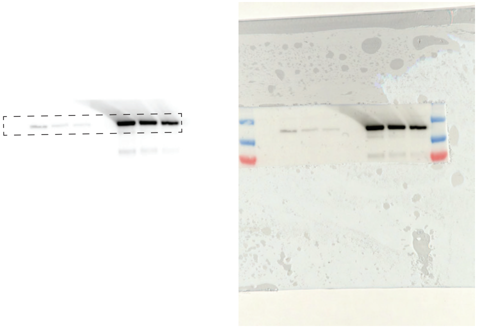

ab: EXOSC10

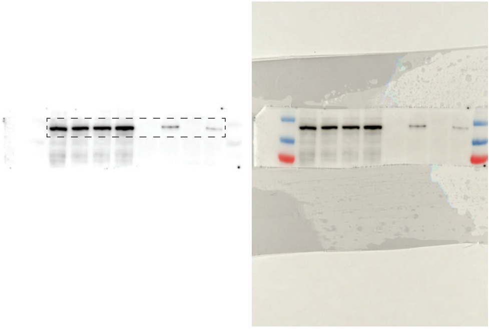

ab: PCID2

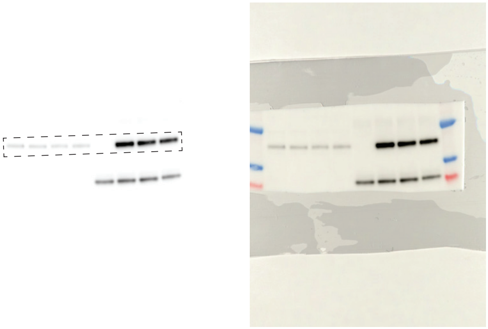

Extended Data Fig. 6a

ab: ZFC3H1

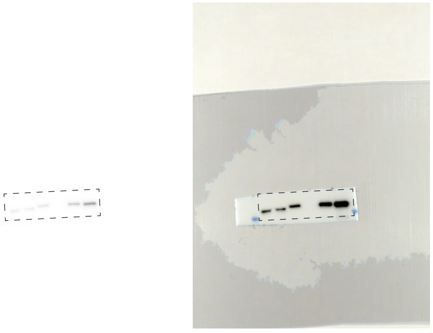

ab: MTR4

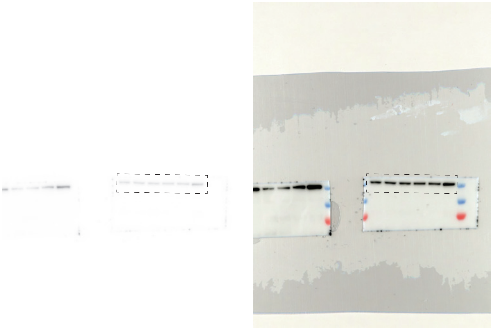

ab: FLAG

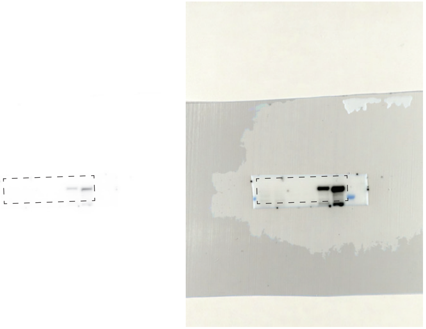

ab: ACTB

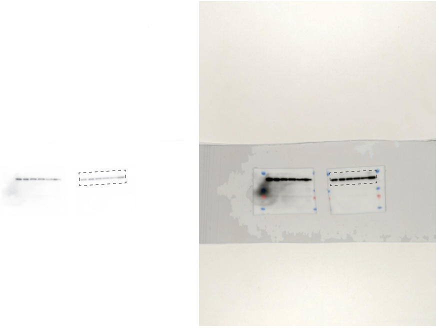

Extended Data Fig. 6b

ab: ZFC3H1

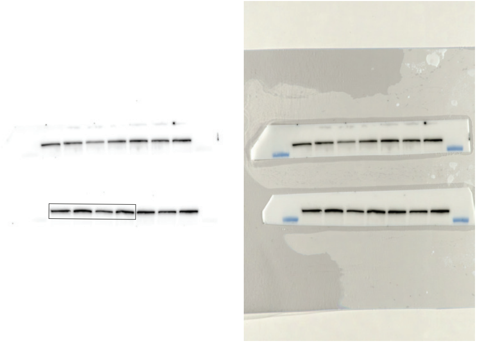

ab: FLAG

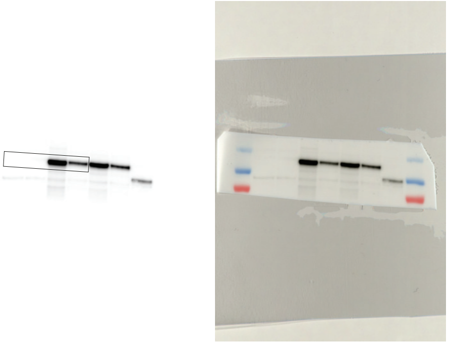

ab: Vinculin

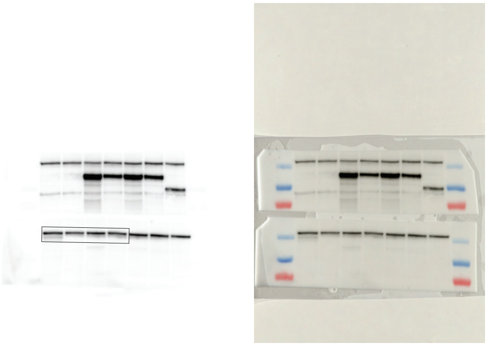

ab: HA

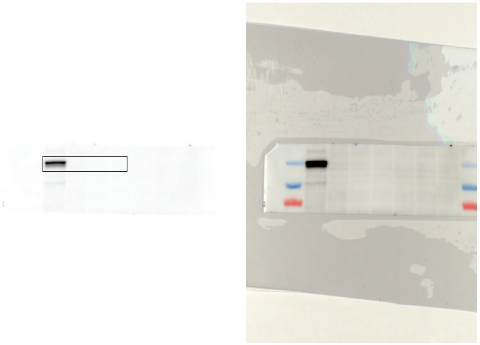

Size marker: PageRuler™ Plus Prestained Protein Ladder (Thermo Fisher)

Extended Data Fig. 6c

ab: ZFC3H1

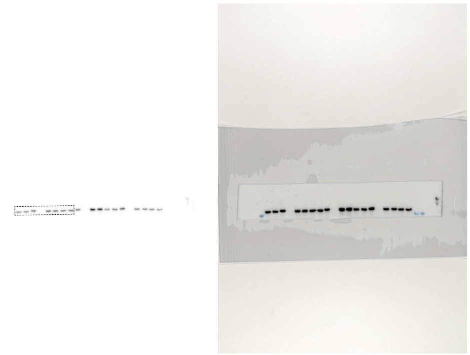

ab: HA

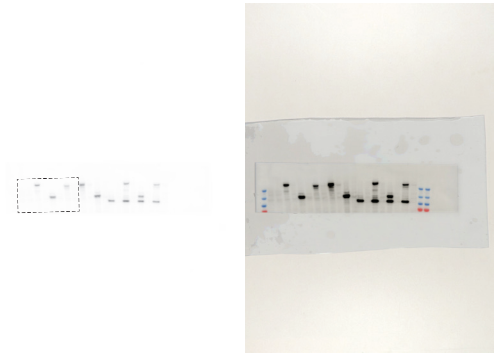

ab: PCID2

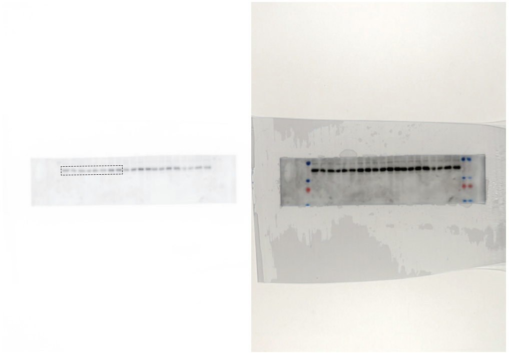

ab: GANP

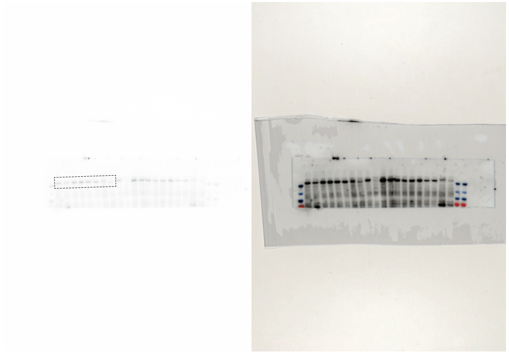

Extended Data Fig. 7a

ab: Tubulin

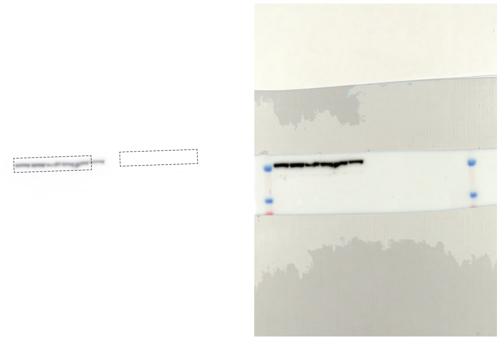

ab: PABPN1

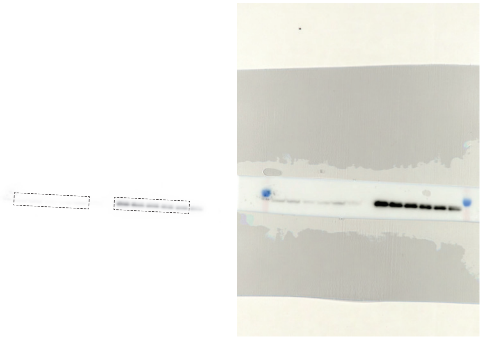

ab: HA-tag

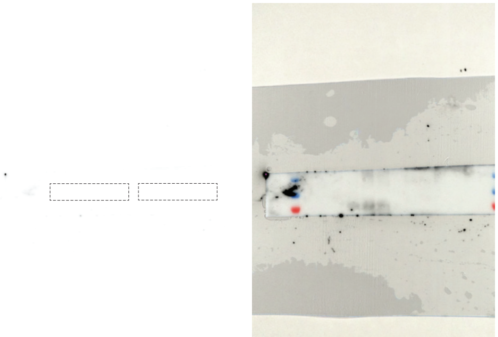

ab: ZFC3H1

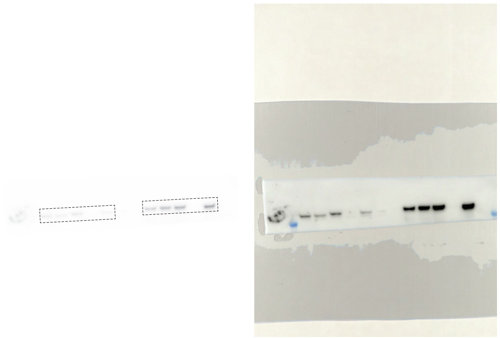

ab: GANP

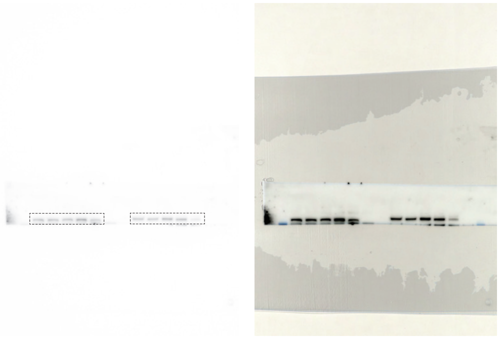

Size marker: PageRuler™ Plus Prestained Protein Ladder (Thermo Fisher)

Extended Data Fig. 7e

ab: ZFC3H1 (top), Tubulin (bottom)

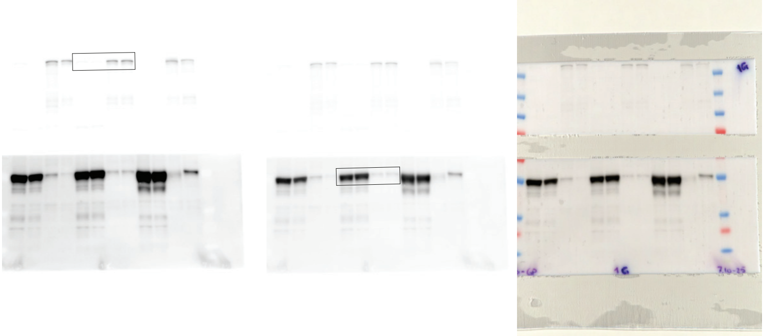

ab: GANP (top), PABPN1 (bottom)

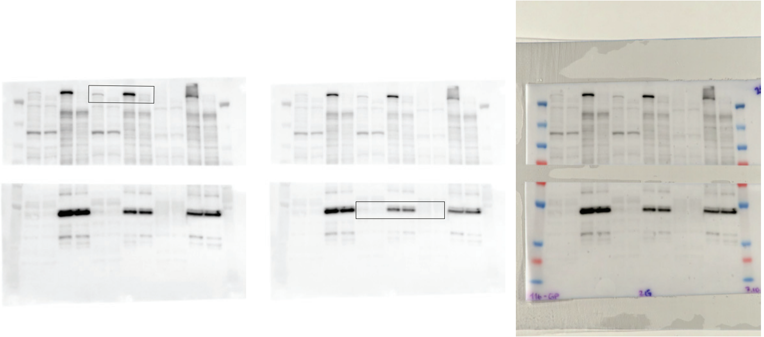

ab: GANP (top), PABPN1 (bottom)

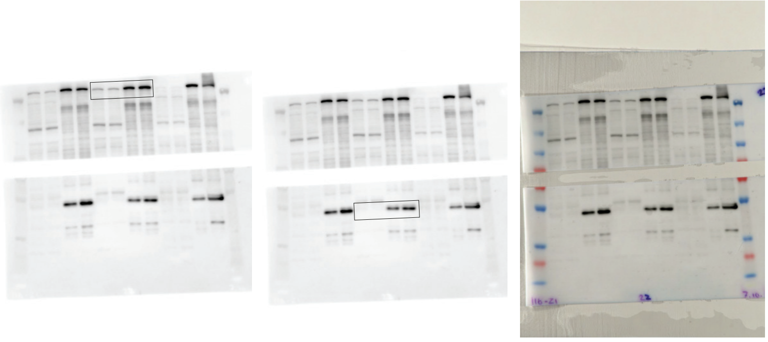

ab: ZFC3H1 (top), Tubulin (bottom)

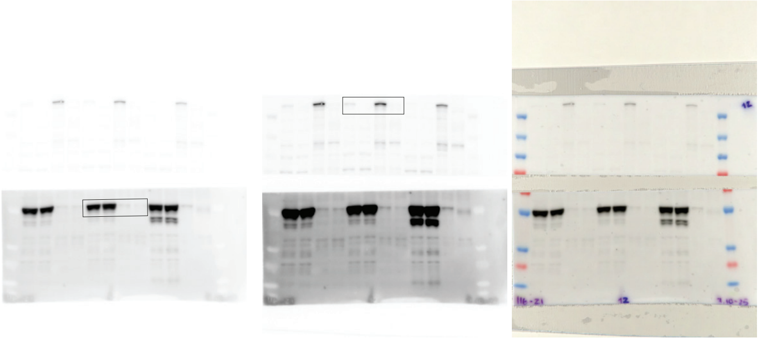

ab: ZFC3H1 (top), Tubulin (bottom)

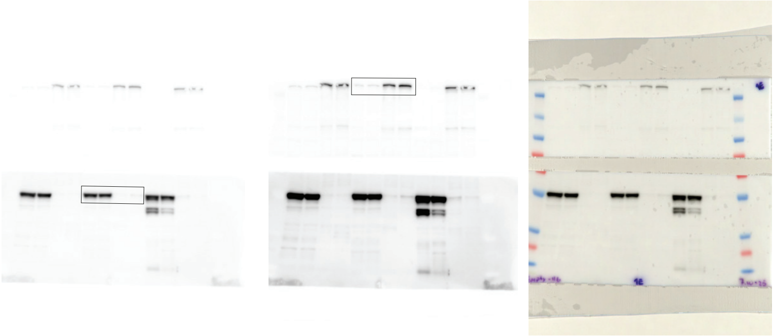

ab: GANP (top), PABPN1 (bottom)

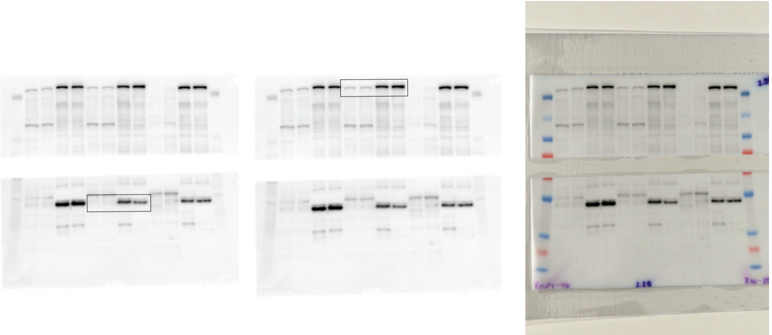

ab: ZFC3H1 (top), HA-tag (middle), Tubulin (bottom)

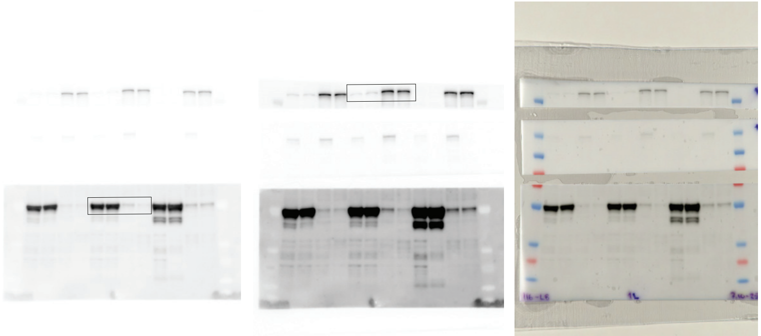

ab: GANP (top), PABPN1 (bottom)

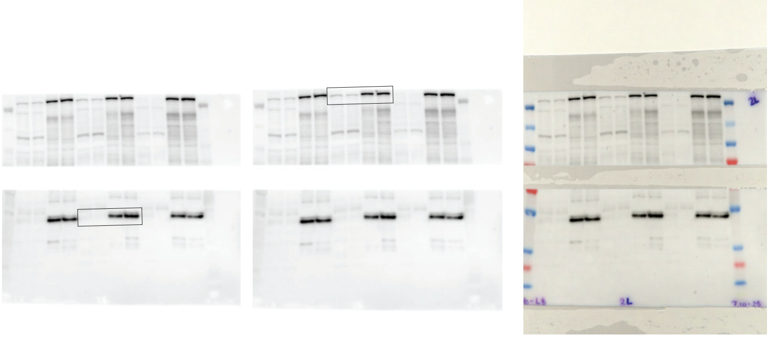

ab: HA-tag

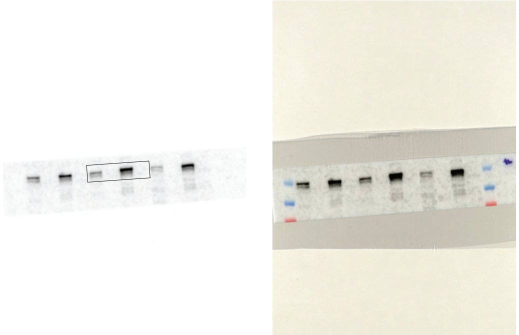

Size marker: PageRuler™ Plus Prestained Protein Ladder (Thermo Fisher)

Extended Data Fig. 8a

ab: FLAG-tag

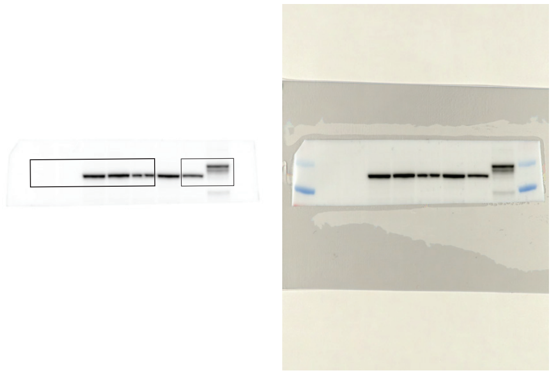

ab: RPLP0

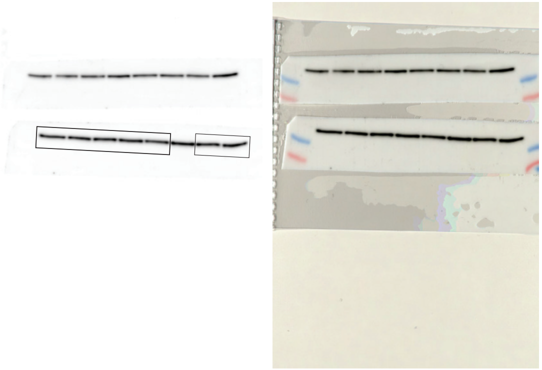

ab: HA-tag

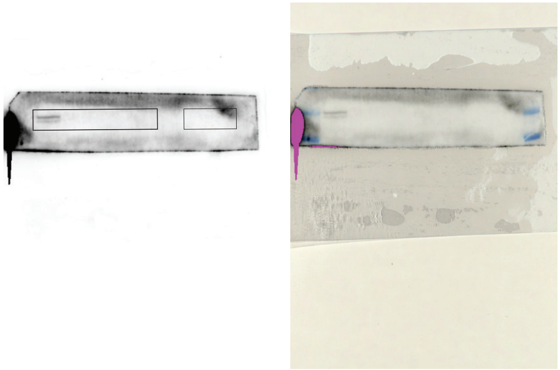

ab: ZFC3H1

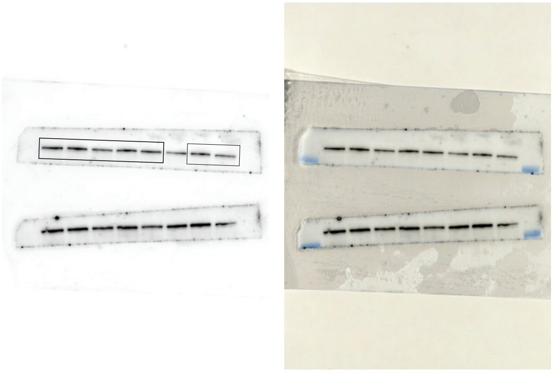

Extended Data Fig. 8b

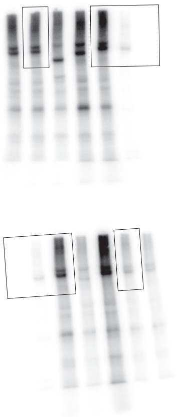

ab: FLAG

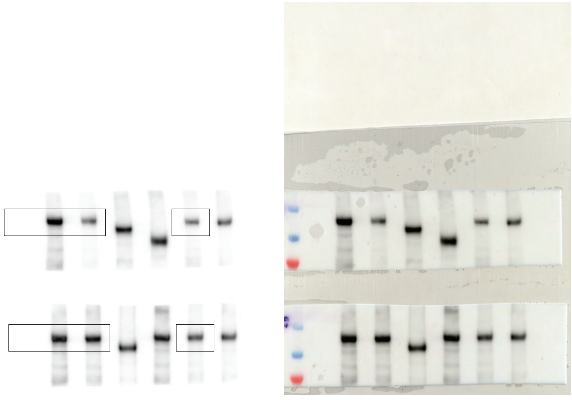

Extended Data Fig. 8d

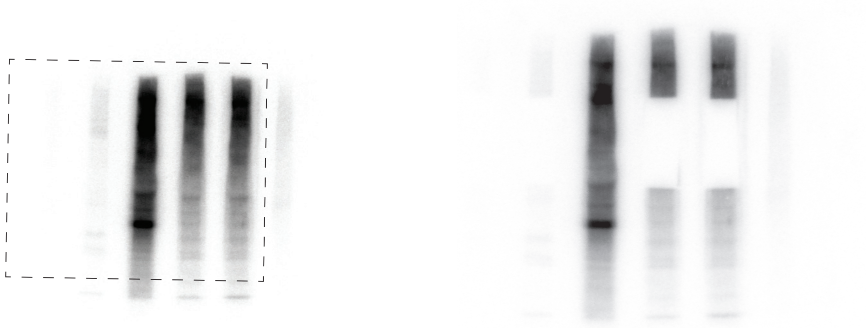

ab: UAP56

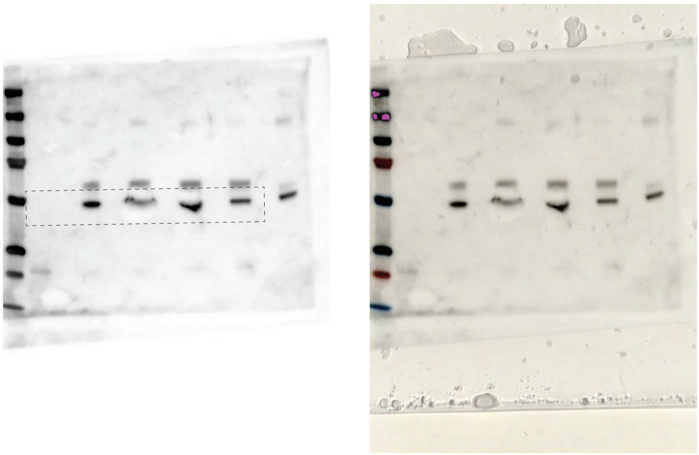

Size marker: PageRuler™ Plus Prestained Protein Ladder (Thermo Fisher)

Extended Data Fig. 8e

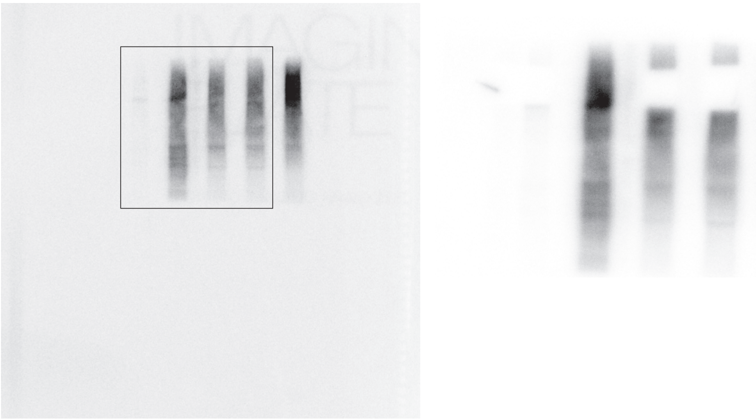

ab: FLAG

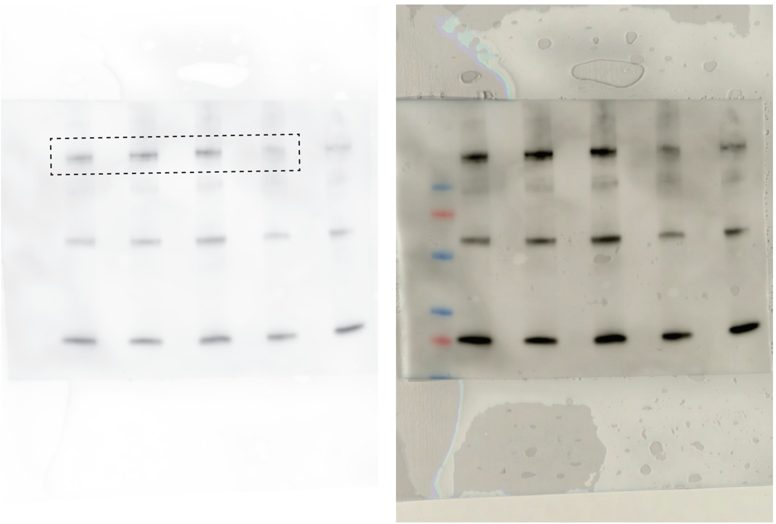

Extended Data Fig. 9a

ab: Puromycin

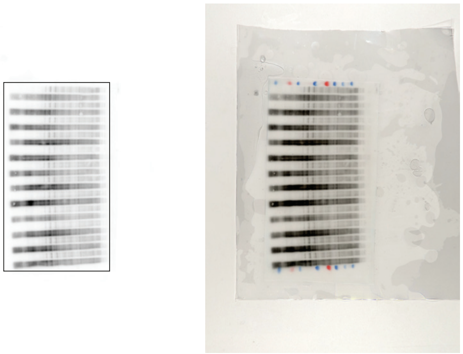

ab: HA

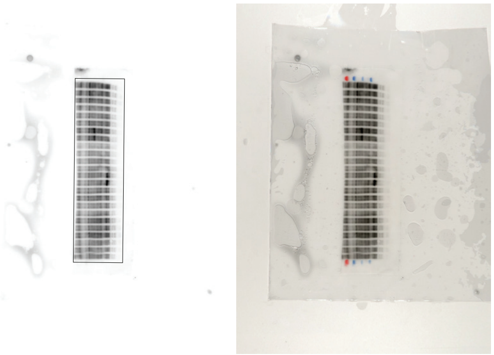

ab: ACTB

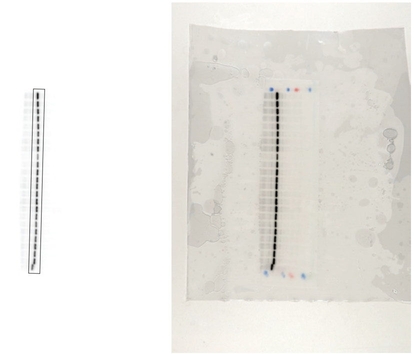

Extended Data Fig. 9c

ab: FLAG

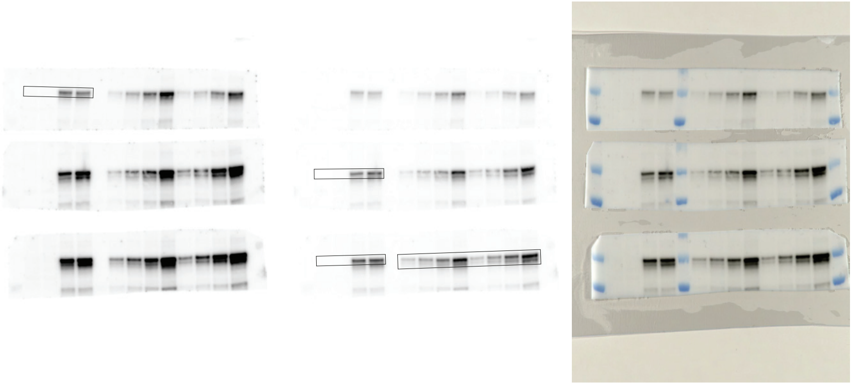

ab: RPLP0

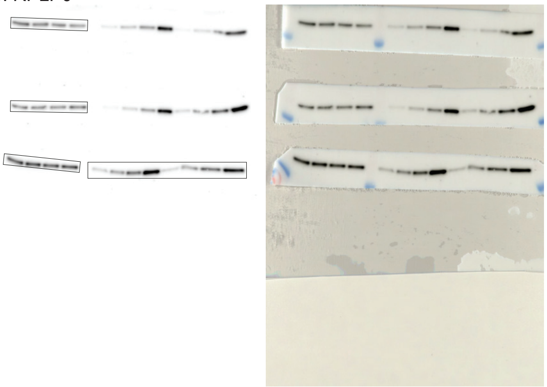

ab: ZFC3H1

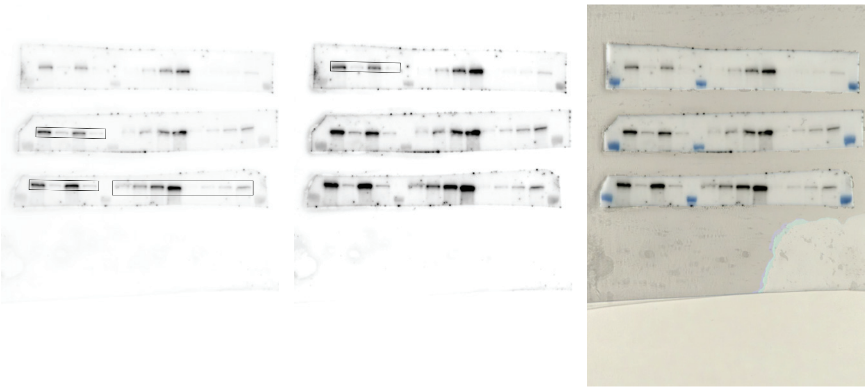

Size marker: PageRuler™ Plus Prestained Protein Ladder (Thermo Fisher)

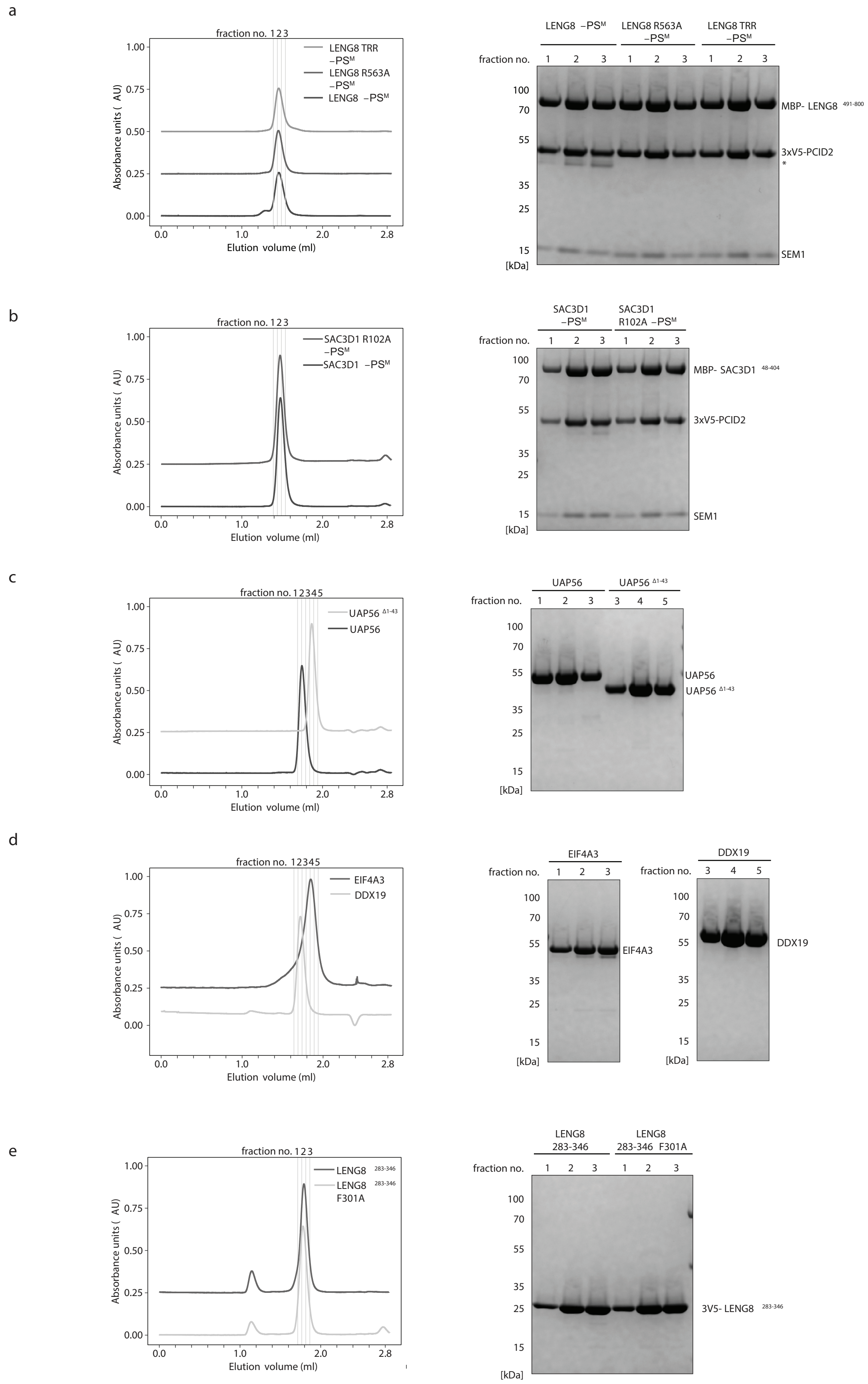
a
fraction no. 1 2 3
LENG8 TRR –PSM
LENG8 R563A –PSM
LENG8 –PSM
Absorbance units (AU)
Elution volume (ml)
fraction no.
MBP- LENG8 491-800
3xV5-PCID2
*
SEM1
[kDa]
b
SAC3D1 R102A –PSM
SAC3D1 –PSM
SAC3D1 –PSM
SAC3D1 R102A –PSM
MBP- SAC3D1 48-404
3xV5-PCID2
SEM1
c
fraction no. 1 2 3 4 5
UAP56 Δ1-43
UAP56
UAP56
UAP56 Δ1-43
d
EIF4A3
DDX19
EIF4A3
DDX19
e
LENG8 283-346
LENG8 283-346 F301A
LENG8 283-346
LENG8 283-346 F301A
3V5- LENG8 283-346

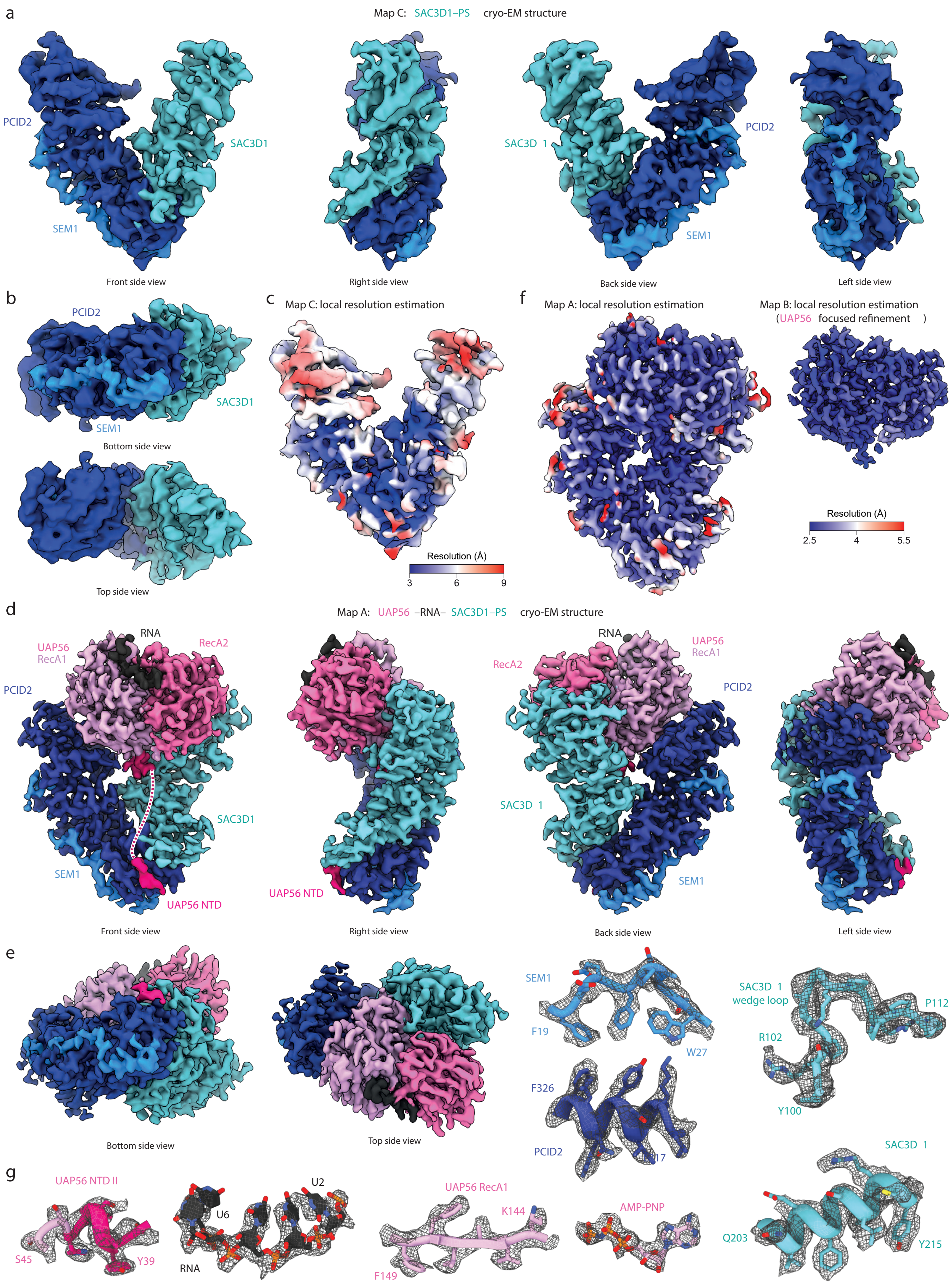
a
Map C: SAC3D1–PS cryo-EM structure
PCID2
SAC3D1
SEM1
Front side view
Right side view
SAC3D 1
PCID2
SEM1
Back side view
Left side view
b
PCID2
SAC3D1
SEM1
Bottom side view
Top side view
c
Map C: local resolution estimation
Resolution (Å)
3
6
9
f
Map A: local resolution estimation
Map B: local resolution estimation
(UAP56 focused refinement)
Resolution (Å)
2.5
4
5.5
d
Map A: UAP56 –RNA– SAC3D1–PS cryo-EM structure
UAP56
RecA1
RNA
RecA2
PCID2
SAC3D1
SEM1
UAP56 NTD
Front side view
UAP56 NTD
Right side view
RNA
RecA2
UAP56
RecA1
PCID2
SAC3D 1
SEM1
Back side view
Left side view
e
Bottom side view
Top side view
SEM1
F19
W27
F326
PCID2
SAC3D 1
wedge loop
P112
R102
Y100
g
UAP56 NTD II
S45
Y39
U6
U2
RNA
UAP56 RecA1
K144
F149
AMP-PNP
SAC3D 1
Q203
Y215

Supplementary Fig. 4 | LENG8–PS, UAP56-NTD–LENG8–PS, UAP56–RNA–LENG8–PS cryo-EM analysis

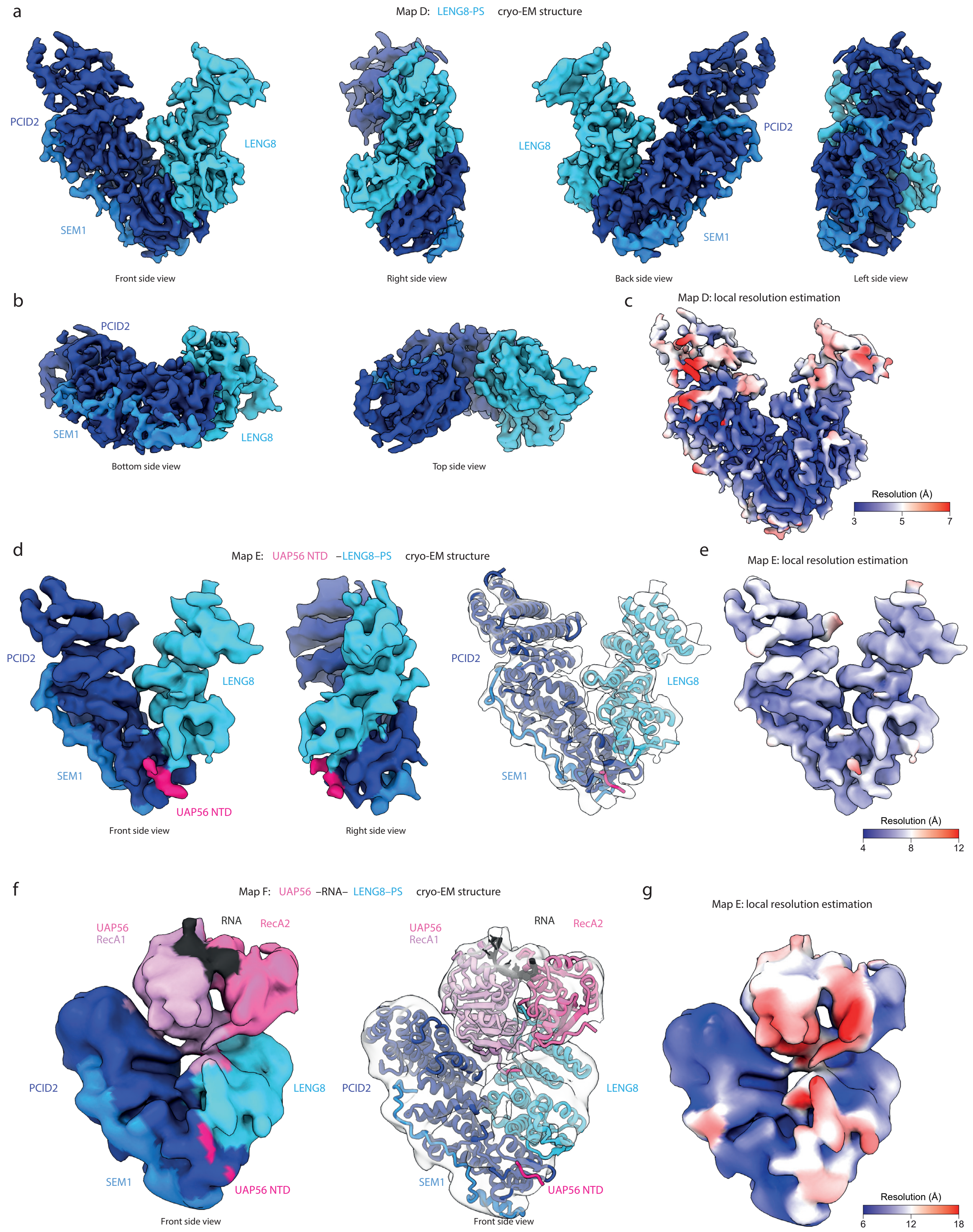

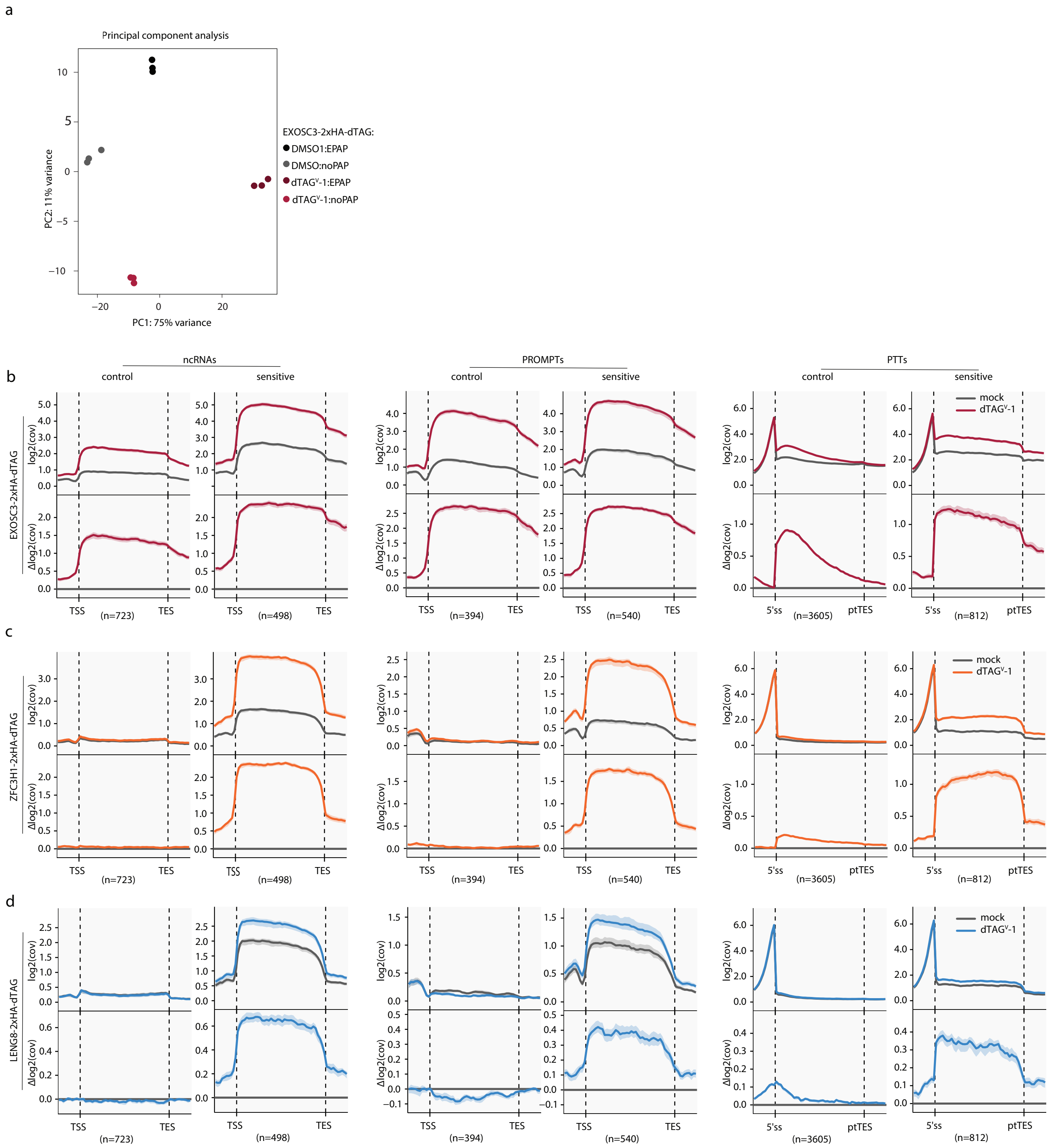
a
Principal component analysis
PC1: 75% variance
PC2: 11% variance
EXOSC3-2xHA-dTAG:
DMSO1:EPAP
DMSO:noPAP
dTAGV-1:EPAP
dTAGV-1:noPAP
b
ncRNAs
PROMPTs
PTTs
control
sensitive
EXOSC3-2xHA-dTAG
log2(cov)
Δlog2(cov)
mock
dTAGV-1
TSS
TES
5'ss
ptTES
(n=723)
(n=498)
(n=394)
(n=540)
(n=3605)
(n=812)
c
ZFC3H1-2xHA-dTAG
d
LENG8-2xHA-dTAG
