# Supplementary material for: Molecular basis of polyadenylated RNA fate determination in the nucleus
Source: Nature. 2026 Jun 17;655(8124):1070–8. doi: 10.1038/s41586-026-10650-0 (PMC13391367; doi:10.1038/s41586-026-10650-0)
Supplement: Supplementary file 10 — Peer Review File [file 41586_2026_10650_MOESM10_ESM.pdf]

nature portfolio

Peer Review File

Molecular basis of polyadenylated RNA fate determination in the nucleus

Corresponding Author: Professor Torben Heick Jensen

Any redactions in this file are there to maintain patient confidentiality, the confidentiality of unpublished data, or to remove third-party material.

This file contains all reviewer reports in order by version, followed by all author rebuttals in order by version.

Version 0:

Reviewer comments:

Referee #1

(Remarks to the Author)
Bugai et al have addressed the molecular basis for distinguishing the fate of polyA+ mRNA in the nucleus towards export to the cytoplasm or degradation in the nucleus. They propose that the RNA exosome targeting complex PAXT harbors a module, LENG8-PS, that is structurally and functionally similar to the GANP-PS trimer of TREX-2. They propose that fate of polyA+ RNA is determined by the competing actions of LENG8-PS and GANP-PS trimers that release mRNA from UAP56 to facilitate either RNA degradation via nuclear exosome, or export to the cytoplasm via the nuclear pore complex.
While the proposed model is attractive and may advance the understanding of polyA+ RNA fate in the nucleus, several aspects are rather speculative and require additional data to support the claims.

1. The authors claim that LENG8-PS constitutes a physical module of PAXT.
-There is convincing evidence to show that LENG8 and to some extent PCID2 interact with ZFC3H1 and MTR4 helicase. However, other subunits of PAXT are notably absent. Since MTR4 is not specific to PAXT and is associated with nuclear exosome, which is abundant among LENG8 and PCID2 interactants (Fig 2B), the only PAXT-specific interactant identified is ZFC3H1. The authors should provide evidence that the LENG8-PS associates with additional subunits of PAXT.
-The authors state that interactions were tested under stringent IP conditions to enrich for high affinity interactants (line 138). The conditions used were 150 mM NaCl and 10 mM KCl together with standard concentrations of detergents. These conditions are not especially ‘stringent’. Key interactions should be tested using more stringent conditions that might support the authors’ conclusion that LENG-PS is a module of PAXT (or ZFC3H1). Similarly, although the use of engineered cell lines is indeed useful for many of the experiments, there is no demonstration of interactions between the endogenous proteins (without tags).
- Supplementary Fig 3e, does depletion of ZFC3H1 alter the distribution of LENG8 in the nucleus ?

2. LENG8-PS functions in PAXT-mediated control of nuclear polyA+ RNAs
- The effect of LENG8 on ZFC3H1-sensitive RNAs is not compelling. In Fig 3C, if cut-offs are applied, how many RNAs are co-regulated by ZFC3H1 and LENG8 ?
- In Fig 3D and E, if I understand the figures correctly, the DE RNAs analysed are specific to each depletion condition separately, and not specifically those co-regulated by ZFC3H1 and LENG8. It would be instructive to analyze co-regulated RNAs. Additionally, in Fig 3d, an FC cut-off is applied, but not in Fig 3e. Indeed, very few LENG8-sensitive RNAs would pass should an FC cut-off be applied.
-in Supp Fig 5j and k, again, it would be useful to analyse the co-regulated RNAs in addition to the population by each factor individually. How many of these RNAs are co-regulated by both factors and do they share the same characteristics?

3. PAXT and TREX-2 contact UAP56-bound RNP to govern nuclear polyA+ RNA fate
- A central premise to the authors’ model is that LENG8 can release RNAs bound by UAP56 for degradation by ZFC3H1 and nuclear exosome. However, very little evidence for this is demonstrated in vivo. For example, in the experiment shown in Supplementary Fig 7a, it would be useful to perform RIP-seq to show that the differentially bound RNAs overlap with PAXT targets.
- While the suggestion that ZFC3H1 protein expression is increased by LENG8 depletion is convincing (Fig 4c, supp 5b),

the converse, that LENG8 protein synthesis is increased by ZFC3H1 depletion, is not compelling. Can the effect be shown by immunoblot ?

Referee #2

(Remarks to the Author)
The manuscript by Jensen, Brennecke, and Plaschka groups proposes that the nuclear RNA decay pathway mediated by PAXT complex contains a TREX-2-like module consisting of LENG8, PCID2, and SEM1 proteins that recognizes pA+ RNPs bound by UAP56 DEAD-box helicase and triggers decay, whereas the canonical TREX-2 complex consisting of GANP, PCID2, and SEM1 recognizes the same UAP56 cue at the nuclear pore to promote export of pA+ RNPs.

The work combines biochemistry, a high-resolution cryo-EM structure of a UAP56:SAC3D1:PS complex in a pre-release state, cell biology with endogenous tagging, and transcriptomics with acute degron depletions to support a unifying model of nuclear pA+ RNA fate. Conceptually, showing that decay and export share the same molecular mechanism comprising UAP56 clamping and SAC3-wedge loop-mediated release is both elegant and broadly relevant to RNA biology and nuclear transport.

The central idea of the study, that PAXT and TREX-2 deploy homologous SAC3-containing modules to act on the same UAP56 interface, biasing decay vs export, certainly appears original and novel. It extends and integrates recent advances in the TREX-2 mechanism and PAXT biology and generates a significant step forward in our understanding of mRNP fate determination in the nucleus. Prior work established TREX-2 association with the pore and UAP56 release in export, and implicated PAXT (ZFC3H1/MTR4) in nuclear retention/decay of short pA+ RNAs; this manuscript connects these mechanisms. Relevant antecedents (also cited by the authors) include TREX/TREX-2/UAP56 functions, and the PAXT/ZFC3H1 literature on nuclear retention and decay of short/intron-poor transcripts. The novelty here is the TREX-2-like module containing LENG8 within PAXT acting on UAP56, the structural pre-release state, and a competition model validated by reciprocal degron phenotypes.

The results presented here should be of immediate interest to multiple communities, including RNA processing/quality control, nuclear export, and structural biology, because they reframe export vs decay as competing outcomes of a shared UAP56-centered mechanism and provide high-resolution structural insight into the “pre-release” state.

I do not see fatal flaws. The study is strong and largely self-consistent, and the labs are leaders in their fields. The cryo-EM structural work is sound and appropriately validated.

Below, I describe major points for the authors to consider addressing to improve this work to the point where it is suitable for publication in Nature.

1) Add orthogonal structural or cross-linking evidence for UAP56:LENG8:PSM (or an endogenous cross-link map) to substantiate the direct parallel to SAC3D1/TREX-2. The authors could not obtain an interpretable map of the LENG8-containing complex due to biased particle distribution (a common and often insurmountable issue in single-particle cryo-EM). However, the study then becomes overly reliant on AlphaFold predictions. Even when validated biochemically through structure-guided analysis, this is risky, and the entire premise of the study rests on the comparison and competition of similar interfaces. More evidence is required, besides structural predictions, to support the mechanisms presented. I would also advise the authors to revisit cryo-EM and attempt adding detergents or exploit tilting to obtain sufficient representation of views for reconstruction. Have the authors considered crystallizing the LENG8:PSM? Sac3:Thp1:Sem1 complex from yeast was first structurally characterized by the Stewart/Hurt labs using crystallography. This appears to be a rigid complex, so perhaps it's worth a shot.

2) The authors really should test the requirement for the wedge loop and PS-binding at endogenous levels in cells (eg. CRISPR knock-ins for LENG8 R563A and H712F or PCID2 depletion) and read out PAXT-substrate behavior via sequencing.

3) The manuscript’s central read-outs, namely the transcriptome-wide shifts in nuclear-retained vs. exported poly(A)+ RNAs and the associated fractionation profiles after acute depletion of PAXT or TREX-2 components, are demonstrated solely in HeLa cells. HeLa harbors unusually high aneuploidy, copy-number variation, and deregulated RNA-processing pathways; its responses may not fully represent those of more genomically stable human cells. I therefore encourage the authors to reproduce the key RNA-seq and nuclear-cytoplasmic fractionation read-outs in at least one additional human cell line with a more typical karyotype (for example, RPE1, HEK293, or HCT116). Confirming that the same classes of short, low-exon transcripts accumulate in the nucleus upon PAXT loss and that reciprocal effects follow TREX-2 perturbation in a second cellular background would significantly increase confidence that the proposed UAP56-centered decay/export switch is a general mechanism rather than a HeLa-specific idiosyncrasy. This replication is strongly recommended to bolster the study’s impact, although I would not regard it as an absolute prerequisite for eventual acceptance.

4) Having read the study several times, I would argue that the authors should clarify the decay-versus-export competition with a focused epistasis experiment. The competition model puts forward that a single UAP56 clamp on nascent pA+ RNPs

can be captured by either the PAXT module (via LENG8:PCID2:SEM1) to route the RNA toward nuclear decay, or by the TREX-2 module (via GANP:PCID2:SEM1) to deliver it to the pore for export. Depleting LENG8 alone shifts short, intron-poor transcripts into the cytoplasm, while depleting GANP alone traps them in the nucleus. These two opposite phenotypes imply mutually exclusive engagement. To test this head-to-head in a single experiment, I suggest an epistasis assay in which both LENG8 and GANP are perturbed simultaneously and the fate of well-characterized reporter transcripts is monitored. For this, the authors can make use of their existing dTAG or auxin-inducible degron alleles for LENG8 and GANP to achieve synchronous, near-complete depletion within 2 to 4 hours. Alternatively, they could combine highly efficient siRNA pools (or CRISPRi guides) and harvest at the same time-point used for the individual knock-downs to control for indirect effects. Nuclear-cytoplasmic fractionation followed by RT-qPCR or short-read RNA-seq to quantify (i) classic PAXT substrates such as PROMPTs, snRNA-adjacent transcripts, or intron-poor lincRNAs; and (ii) canonical export substrates of comparable length as internal controls. smRNA FISH at a handful of loci, such as NEAT1_1, HIST1H1C, and a well-behaved short mRNA, can then visualize retention versus export. A UAP56 co-IP after double depletion can then test whether neither module can stably occupy the helicase, as predicted. If LENG8 and GANP truly compete for the same UAP56-bound RNPs, removing both should negatively impact both decay and export pathways. Transcripts (a few examples suggested above) would accumulate as unprocessed nuclear pA+ species, potentially yielding larger nuclear retention than either single knockdown. However, if the modules act on different RNP pools, the double knock-down should produce an additive or intermediate phenotype, not a synergistic block. Because the single-factor degron data already point strongly to competition, I do not suggest that this double-perturbation experiment is strictly essential for acceptance; however, even a small-scale test at a few loci would provide direct functional evidence that the two SAC3-domain assemblies are mutually exclusive gatekeepers of UAP56 and, in the opinion of this reviewer, would significantly strengthen the paper's central mechanistic claim.

5) Finally, the authors should test the model in the background of acute PABPN1 depletion to determine whether the proposed UAP56:LENG8 (PAXT) vs TREX-2 competition depends on protected, PABPN1-coated poly(A) tails. PABPN1 binds and organizes nuclear poly(A) tails, promotes canonical extension by PAP, and limits premature deadenylation; its loss yields shortened/heterogeneous tails and greater 3′-end exposure. If the mechanism preferentially engages well-protected pA+ RNPs, PABPN1 loss should diminish PAXT engagement or shift export/decay balance; conversely, if oligo-adenylated/poorly protected 3′ ends favor PAXT capture, depletion should promote and accentuate PAXT phenotypes. Concretely: perform dTAG/siRNA PABPN1 +/- LENG8 or ZFC3H1 (and/or GANP) perturbations; quantify nuclear/cytoplasmic distribution and decay rates for sentinel short/intron-poor transcripts; and profile poly(A) tail lengths (e.g., PAL-seq/TAIL-seq or long-read tail-length calling). Co-IP of UAP56 with PAXT vs TREX-2 under PABPN1 depletion would further test whether 3′-end protection modulates complex choice. This experiment seems quite essential, and PABPN1 is presently somewhat neglected in the current version of the manuscript, given its centrality in 3′ end processing.

Minor points are described below.

a) Whilst I appreciate the beauty and aesthetics of structural figures, the thickness of the cartoons representing secondary elements obscures relevant residues and makes interfaces difficult to discern. This concerns figures 1h, 2e, S1a (do all subunits need to be various shades of blue?). Please consider including more views in the SI. I would emphasize that the clarity and contrast in structural figures are more important than aesthetics.

b) In biochemical pulldown experiments, quite often the msot obvious explanation of the loss of binding can be a trivial one such as poor stability of a truncated construct or buffer conditions. The authors should consider their controls more carefully.

c) Lines 118-119: "...pre-positions R102 adjacent to F381… priming it to displace this residue…" Aside from a lack of clarity in what is priming or displacing what, there is essentially no evidence to support this structural claim. Nevertheless, it is quite central to the entire mechanism. How can one residue 'prime' the displacement of a neighboring one? Please either support with evidence or mark this statement as speculative.

d) The authors lean rather heavily into AlphaFold-based structure predictions, but, in my view, these require quite a bit more work to have confidence than simply including PAE plots, which are rather uninformative for interfaces. For key predictions, the authors should generate 25 independent predictions and assess their agreement by superposition.

e) Lines 165-166: "...a conserved motif of two alpha-helices…" Structural comparisons should be supported by structural predictions rather than sequence alignments.

f) Line 268: "...ensure specificity for UAP56," Here, the authors should test more than just EIF4A3 to make the case for specificity for UAP56.

g) Lines 288-289: "...due to overloading of ribosome by ncRNAs escaping from the nucleus." The authors should clarify here how and in what circumstances ribosomes can become overloaded by non-coding RNAs. Are these capped?

THERE ARE NO PRIVATE COMMENTS TO THE EDITOR.

Referee #3

(Remarks to the Author)
In this manuscript, Bugai et al. describe two TREX-2-like modules—LENG8-PS and SAC3D1-PS—and propose that

LENG8-PS competes with TREX-2 for binding to the export factor UAP56, thereby regulating nuclear retention and decay of typically short polyadenylated RNA species, which often result from spurious transcription (PROMPTs) or premature cleavage and polyadenylation (ptRNAs).

The authors were unable to solve the 3D cryoEM structure of UAP56-bound LENG8-PSM (in which only the SAC3 domain of LENG8 is included). However, they provided evidence of its cryoEM structure in 2D and AlphaFold predictions that are consistent with a cryoEM structure of UAP56-bound SAC3D1-PSM, which they successfully solved at 2.6 to 4.5 Å resolution. Combined with additional AlphaFold predictions with the core PAXT factor ZCF3H1, the authors generated multiple LENG8 variants defective in (i) activating UAP56 ATPase activity (i.e., LENG8(R563A)), (ii) binding PCID2 (i.e., LENG8(H712F)), and (iii) binding ZFC3H1 (i.e., LENG8(F301A)). They also generated a ZFC3H1 deletion variant unable to bind LENG8, i.e., ZFC3H1(Δ730-747). The authors then combined the use of these variants with rapid depletion of endogenously tagged proteins to nicely demonstrate how the LENG8-ZFC3H1 interaction is key to mediating the degradation of PAXT targets transcriptome-wide. This aspect of the manuscript is overall solid. The authors then use similar tools to propose that PAXT-mediated RNA decay is mediated by UAP56. However, we are unconvinced that the experiments provided here support this claim (see major comment #1). The authors also unveiled interesting feedback loops between LENG8 and ZFC3H1 that likely function to maintain PAXT activity when one or the other protein is depleted.

This manuscript combines numerous state-of-the-art approaches to enrich our understanding of how PAXT is (or could be) recruited onto target RNAs and, more importantly in our opinion, how nuclear RNA decay competes with nuclear RNA export. While most experiments are well conducted and interpreted, we identified flaws or uncertainties that need to be addressed.

Major comments
1) UAP56 can be recruited onto RNPs via ALYREF, which can be recruited via the EJC in the case of spliced RNPs, via RBM33 in the case of single-exon mRNAs with a 5'-GC-rich region (Thomas et al., Genes&Dev. 2022), or via SLBP in the case of replication-dependent histone mRNAs (Fan et al., EMBO J. 2019). However, it is unclear if and how UAP56 is recruited onto the RNA species that are typically degraded by PAXT/LENG8, i.e. PROMPTs and ptRNAs. Beyond this knowledge gap, other elements make it doubtful that PAXT/LENG8 binds and degrades its RNA targets via UAP56, which seems essential to explain functional competition with TREX-2:
1a) In Fig. S7a, reduced amounts of RNAs in anti-LENG8(R563A) IPs relative to anti-LENG8(WT) IPs are accompanied by a reduced IP efficiency of LENG8(R563A) relative to LENG8(WT). Thus, it is unclear if the R563A mutation impairs the ability of LENG8 to bind RNA via UAP56 in cells. Notably, we are unsure why the authors hypothesized that this mutation, which they claim blocks the release of UAP56 from RNA in vitro (although this was not demonstrated), would reduce UAP56-mediated binding of LENG8 to RNA. Wouldn't it more straightforward to test a LENG8 variant that does not bind UAP56? Available or predicted structure determinations of TREX-2-like complexes could very likely provide the structural insights required for the design of such a variant. Additionally, the authors should investigate the binding of LENG8 to PAXT targets rather than to total-cell RNAs, especially if, as suggested by data in Fig. S7f,g, LENG8 also binds EIF4A3, which is usually deposited on spliced mRNAs as an EJC constituent.
1b) Figs. S4i and S7b indicate that the R563A mutation reduces the association of LENG8 with the RNA exosome and possibly with MTR4 (but not with ZCF3H1). Thus, we are unconvinced that the "release of RNA from UAP56 is central for its PAXT-mediated turnover". Additionally, the authors should explain how UAP56 ATPase activity is linked to its release from RNA, and why they think that release of UAP56 from RNA would be required for exosome-mediated RNA decay.
1c) While the authors convincingly demonstrated the association in cells of ZFC3H1 with LENG8 and PCID2, they did not show that LENG8 (as part of LENG8-PS module), bridges ZFC3H1 to UAP56. Supporting this model, data from Table S2 indicate that UAP56/DDXD39B co-immunoprecipitates with ZFC3H1(WT) and less efficiently with ZFC3H1(Δ730-747). However, the authors did not investigate the significance of this result and did not test if the co-IP was resistant to RNase. As was shown for the ZFC3H1-binding-defective variant of LENG8 (i.e., LENG8(F301A)), it seems important to demonstrate that a UAP56-binding-deficient variant of LENG8 fails to support the decay of PAXT targets.
1d) Table S2 indicates that EIF4A3 (and other EJC constituents) as well as other RNA helicases that may share amino-acid similarity with UAP56 are also enriched in anti-ZFC3H1(WT) IPs relative to ZFC3H1(Δ730-747) IPs. This raises the possibility that LENG8 recruits PAXT onto target RNAs via different RNA helicases, the identity of which may depend on the RNA species. However, focusing on UAP56 would be sufficient if the authors can demonstrate UAP56 is key to mediate RNA decay by LENG8/PAXT.

2) The authors show in their cryoEM structures that UAP56 binds LENG8-PSM and SAC3D1-PSM in a closed RNA-bound conformation. This is in contrast to the cryoEM structure of TREX-2M, where UAP56 is not clamped onto RNA. First, how could the authors capture a UAP56–TREX-2M complex in which UAP56 was released from RNA if the complex was formed in the presence of the non-hydrolysable ATP analog AMP-PNP? Second, what is the biological relevance of the distinct RNA-binding states of UAP56 in TREX-2 and TREX-2-like modules? Third, the methods section, but not the main text or figures, mentions 'UCM1' and 'N-UBM' in the preparation of cryoEM samples. Please define these terms and their significance.

3) Fig. S1d,e show that much less UAP56 is engaged with than without LENG8-PSM or SAC3D1-PSM. Likewise, Fig. S7g shows that much less UAP56 (or EIF4A3) is engaged with LENG8(491-800) than with LENG8-PSM. How could the authors assume that lower UAP56/EIF4A3 concentrations would not influence the number of ATP molecules hydrolyzed per protein and per sec, as measured in Figs. 1c, 1d, and S7f?

4) Our interpretation of Fig. S6c is that only RNAs in Cluster 4 have increased C/N ratios after rapid ZFC3H1 depletion or, to a lesser extent, after rapid LENG8 depletion. In other words, ZFC3H1 and LENG8 retain only a very small fraction of PAXT

decay targets in the nucleus. The authors should clarify why they focus on Cluster 1 and do not discuss, in particular, Cluster 4.

5) What data support the statement in the Discussion that “TREX-2 seemingly grants export to any pA+ RNA provided that this pA+ RNP can make its way to the NPC”? Indeed, the main text reports that “[rapid reduction of GANP protein] resulted in the downregulation of short pA+ RNAs with few introns”, and only this set of RNAs seems to have been included in the “downregulated” genes shown and analyzed in Fig. 4b. Additionally, what data support the statement that “Both TREX-2 and PAXT engage UAP56-bound pA+ RNPs indiscriminately”? The authors argue at length that PAXT targets specific RNA species, making the “indiscriminately” comment contradictory.

6) The authors did not acknowledge the existence of a UAP56–LENG8-PSM cryoEM structure (Clarke et al., Nat Comm. 2025, where it is referred to as TREX-2.1/DDX39B) and a preprint investigating the role of LENG8 in nuclear RNA retention and PAXT-mediated RNA decay (Tian et al., bioRxiv. 2025, where LENG8-PS is referred to as the Repressor of EXport complex, or REX). Both papers were very recently published, so the authors possibly missed them during the submission of this manuscript.

Minor comments:
1) The Jensen lab recently demonstrated that PAXT-mediated decay of short RNA targets requires the binding of ZFC3H1 to cap-associated protein ARS2 (Polák et al., Cell Reports. 2023). It would be interesting to discuss whether ARS2-mediated and LENG8-mediated recruitment of PAXT typify different RNAs—for example, based on “how short” they are.

2) As a general comment, the structures might be better annotated. For example:
o Residues mentioned to describe the SAC3D1–UAP56 interaction should be shown in Fig. 1h. Minimally, SAC3D(P111) (and the corresponding residue in GANP), UAP56(R135), and UAP56(K334) should be added. Otherwise, it is difficult to understand/visualize how these proteins bind.
o Where are the ‘wedge loops’ in the AlphaFold predictions (Fig S1a) or the cryoEM structure (Fig. 1g).
o Where in Fig. S2e are the regions selected in S2g.
o Fig. S4d. It would be helpful to annotate where the ZFC3H1 amino acids 730-747 are localized in the predicted alignment error maps. We imagine that they align with the blue region in the A/B intersecting areas.

3) Unlike what is stated in the text, based on results shown in Figs. S3b and S3c, the co-IP of RNA exosome constituents with LENG8 or with PCID2, but not with ZFC3H1, is partially sensitive to RNase digestion (although sometimes below a selected P-value of 0.01). Could this reflect the simultaneous association of PAXT-bound LENG8-PS complexes with multiple UAP56 molecules (or other helicases?) separated by RNA stretches on given transcripts, only one/some of which engage the RNA exosome?

4) Page 5 lines 25-26. We believe that the authors mean that the alpha-helices are in a region N-terminal “to” (and not “of”) the SAC3 domain.

5) Efficient acute depletion of LENG8 was not demonstrated in Fig. S6a, but it should be.

6) In the Discussion, the authors propose that PAXT may release UAP56 from longer RNAs, thereby increasing their nuclear export via TREX-2. How could this be possible if TREX-2 requires UAP56 for docking?

7) Could the authors speculate on the molecular function of the presumably cytoplasmic UAP56–SAC3D1–PS complex?

8) The supplementary tables require a description of each column. In their current state, it is often hard and sometimes impossible to figure out what each column corresponds to.

9) Figure legends should specify the number of biological replicates used in each experiment.

Referee #4

(Remarks to the Author)
I co-reviewed this manuscript with one of the reviewers who provided the listed reports.

Version 1:

Reviewer comments:

Referee #1

(Remarks to the Author)
The authors have satisfactorily addressed the comments raised in the initial review. They have clarified several points in the text and included a number of additional experiments that further support their conclusions. Overall, these revisions have strengthened the manuscript and improved its clarity.

Referee #2

(Remarks to the Author)
I thank the authors for their thorough and constructive engagement with all of my comments. The revised manuscript is substantially strengthened by the inclusion of new cryo-EM structures, improved complementation assays at near-endogenous expression levels, and replication of key findings in HCT116 cells. The study now provides a compelling structural and functional framework for nuclear pA+ RNA fate determination centered on UAP56-bound RNPs. I discuss each of my original points below.

1) Structural evidence for UAP56:LENG8:PSM
The authors have gone well beyond what I envisaged. Four (!) new cryo-EM structures-apo LENG8-PS (3.5 A), apo SAC3D1-PS (3.6 A), LENG8-PS bound to the UAP56 NTD (4.9 A), and LENG8-PS bound to RNA-clamped UAP56 (6-12 A) now replce the AlphaFold predictions that were my primary concern. The experimental density for the clamped state, although at lower nominal resolution, unambiguously establishes that LENG8-PS engages RNA-clamped UAP56 in the same manner as SAC3D1-PS at 2.6A. Together with the new biochemical validation of the UAP56-LENG8-PS interface, this fully resolves my concern about over-reliance on computational predictions.

2) Endogenous-level complementation assays
I appreciate the authors' efforts to titrate doxcycline induction to achieve near-endogenous expression of all LENG8-3xFLAG variants. The strategy using the LENG8-3xFLAG line as a reference for endogenous levels is a reasonable alternative to CRISPR knock-in, and the new western blot data in supplementary section demonstrating comparable expression levels across constructs is reassuring. The recapitulation of the original rescue phenotypes under these controlled conditions with wild-type LENG8 rescuing, and F301A, R563A, and TRR mutants failing to rescue strengthens the mechanistic conclusions. I accept the authors' rationale that RT-qPCR across seven diverse PAXT targets is sufficient for this assay without full RNA-seq.

3) Replication in a second cell line
The generation of 2xHA-dTAG LENG8, GANP, and ZFC3H1 lines in HCT116 cells, together with IP-MS and fractionated pA+ RNA-seq, represents a significant addition of experimental data. The agreement between HeLa and HCT116 in protein-protein interaction profiles and in the nuclear/cytoplasmic redistribution of mono-exonic and low-exon-content RNAs upon LENG8, ZFC3H1, or GANP depletion effectively nullifies concerns about HeLa-specific artefacts. I consider this point fully and convincingly addressed.

4) LENG8/GANP double-depletion epistasis
I acknowledge the considerable effort the authors invested in attempting these experiments across multiple cell-line configurations. The observation that dual LENG8/GANP depletion (in HCT116) and dual ZFC3H1/GANP depletion (in HeLa) both result in severe growth impairment and rapid transcriptional shutdown is itself informative. The authors' interpretation that simultaneous loss of both SAC3-domain modules prevents UAP56 recycling, thereby collapsing the RNP biogenesis cycle is consistent with the competition model and with the published phenotype of the FLAG-UAP56 M1 mutant that cannot engage either module.

Although the intended epistasis readout could not be obtained, I would encourage the authors to incorporate the supporting data from the rebuttal (the RT-qPCR of nascent transcripts and the intron-count analysis from the ZFC3H1/GANP double depletion) into the supplementary material. The transcriptional shutdown phenotype upon dual depletion constitutes positive evidence for the UAP56-recycling model and, in my view, would meaningfully strengthen the paper's central claim. I leave this as a strong recommendation, not a requirement for acceptance. Maybe the authors are saving this for a follow-up, but I think it is really helpful to include here.

5) PABPN1 and poly(A) tail protection
The authors have performed the requested PABPN1/LENG8 co-depletion experiment and obtained a clear result showing co-depletion does not increase PAXT substrate accumulation beyond the individual depletions, which suggests that PABPN1 and LENG8 operate sequentially rather than in parallel. The proposed model in which PABPN1 acts downstream of LENG8-PS-mediated UAP56 release to facilitate exosome engagement with the RNA 3'end is logical and sits well with published findings from the Jensen laboratory and others.

I understand the authors' reluctance to open a full investigation of nuclear PABPs within this manuscript. However, I would strongly suggest (again) that the PABPN1/LENG8 co-depletion data be included in the supplementary material, accompanied by a few sentences contextualizing the sequential-action model. This finding provides a valuable mechanistic boundary condition that readers of the paper would benefit from, and its omission would leave the PABPN1 connection unnecessarily opaque given that the experiment has already been performed and the result is clean. These reports will be published together with the manuscript so why not include?

Pending these minor revisions, I am supportive of publication in Nature.

Referee #3

(Remarks to the Author)
Nature manuscript 2025-07-17305A

"Molecular basis of polyadenylated RNA fate determination in the nucleus".

The authors have performed additional work to address concerns raised by each reviewer. We believe that our comments were satisfactorily addressed in general. Nonetheless, we still have a few comments and a recommendation for improvement.

Major comments:
- We previously asked why release of UAP56 by LENG8-PS would be required for exosome-mediated decay. The authors responded as follows: “However, we would like to clarify that we do not propose that release of UAP56 from RNA is directly required for exosome-mediated RNA decay. Instead, we suggest that UAP56 release is necessary to prevent the RNA from engaging TREX-2 and being exported. In this model, UAP56 removal indirectly favors nuclear RNA retention and decay by blocking productive recruitment of the export machinery, rather than by actively promoting exosome function.” We are, however, unconvinced by this response. What would be the purpose of LENG8-PS simultaneously releasing UAP56 from RNA and recruiting the exosome via PAXT if both actions were not functionally connected? Are the authors suggesting that LENG8-PS sensitizes RNAs to decay in two independent mechanisms: possibly, (1) via the release of UAP56 to block nuclear RNA export, and (2) via the recruitment of PAXT on RNA, which would only directly lead to decay if PAXT were recruited close to the RNA 3’-end?
- The authors write on line 104 that the new « TRR » mutation targets the UAP56-binding interface of LENG8. We could not find any supporting information in the manuscript. The authors’ response to our comment #1 indicates that they use a “structure-guided approach” to identify which residues to mutate. This needs to be clarified and properly illustrated in the manuscript.

Minor comments:
- We find it confusing when the authors state in the Abstract that LENG8-PS and GANP-PS are “functionally” equivalent. We suggest they change “functionally” by “biochemically” (as used on line 152). Alternatively, the authors could clarify that what they refer to is the shared ability of LENG8-PS and GANP-PS to release UAP56 from RNA (as stated on lines 62-63). Otherwise, LENG8-PS and GANP-PS fundamentally differ in their ability to mediate nuclear RNA decay or export, respectively.
- As suggested by the authors in their response to our comment #1, it would be helpful to the reader if they included an explanation for how ATP hydrolysis in UAP56 ATPase activity assays relates to UAP56 rebinding to RNA after release.
- The statement that “only ZFC3H1WT efficiently suppressed the accumulation of PAXT-substrates” (Lines 236) does not seem correct. Fig. S9i shows that ZFC3H1Δ730-747, which fails to bind LENG8, retains about half the activity of ZFC3H1WT. It most likely reflects ARS2-dependent LENG8-independent activity of ZFC3H1, as discussed on lines 239-246. The authors concluded that LENG8 and ARS2 have redundant actions on ZFC3H1-mediated decay of the same pool of RNAs. We would argue that the actions are only “partially” redundant since mutating the sites of ZFC3H1 that bind LENG8 or mutating those that bind ARS2 is sufficient to partially stabilize PAXT targets.
- The authors should clarify in the main text how they define “PAXT substrates” (or “PAXT-sensitive RNAs”) when this concept is first introduced. We believe this occurs on line 236, which refers to Fig. S9i, which in turn refers to the Methods section. The methods state: “Due to the strong correlation between coverage changes of LENG8- and ZFC3H1-depletions (Fig. 3c), ‘PAXT sensitive’ TUs were defined as upregulated in either of the two depletions.” Additionally, the authors should make it clear if they included all the pA+ PPT transcripts defined based on ZFC3H1 depletion only (see lines 226-231).
- The number of biological replicates is still missing from most figure legends (for example Figs 1c, 1d, 2a, 2f, 2g, 3c, 3d, 3e, 3f, 4b, 4c, 4d). This information should be included.

Lynne Maquat and Xavier Rambout

Referee #4

(Remarks to the Author)
I co-reviewed this manuscript with one of the reviewers who provided the listed reports.

Version 2:

Reviewer comments:

Referee #3

(Remarks to the Author)
We are satisfied with the authors’ responses and support publication of their work in Nature.

However, we strongly recommend that the authors update their model figure to better reflect the proposed mechanism, which involves the dual role of LENG8: (i) preventing the nuclear export of short RNAs by displacing UAP56, and (ii) promoting their decay by recruiting PAXT.

Referee #4

(Remarks to the Author)
I co-reviewed this manuscript with one of the reviewers who provided the listed reports.

Open Access This Peer Review File is licensed under a Creative Commons Attribution 4.0 International License, which permits use, sharing, adaptation, distribution and reproduction in any medium or format, as long as you give appropriate credit to the original author(s) and the source, provide a link to the Creative Commons license, and indicate if changes were made.
In cases where reviewers are anonymous, credit should be given to 'Anonymous Referee' and the source.
The images or other third party material in this Peer Review File are included in the article's Creative Commons license, unless indicated otherwise in a credit line to the material. If material is not included in the article's Creative Commons license and your intended use is not permitted by statutory regulation or exceeds the permitted use, you will need to obtain permission directly from the copyright holder.
To view a copy of this license, visit https://creativecommons.org/licenses/by/4.0/

We thank the three referees for their genuine interest in our study and for the constructive and helpful comments. Responding to these has substantially improved the manuscript, which is now enriched with new data in two main areas:

1. **Substantiation and further characterization of the role of the LENG8-PS module in PAXT biology**
2. **Substantiation of our model that nuclear RNA sorting is centered around UAP56-bound RNPs**

Please find below the referee comments and our point-by-point responses in blue and black text, respectively.

Yours sincerely,
Clemens, Julius and Torben

Referee #1 (PAXT connection)

Remarks to the Author:
Bugai et al have addressed the molecular basis for distinguishing the fate of polyA+ mRNA in the nucleus towards export to the cytoplasm or degradation in the nucleus. They propose that the RNA exosome targeting complex PAXT harbors a module, LENG8-PS, that is structurally and functionally similar to the GANP-PS trimer of TREX-2. They propose that fate of polyA+ RNA is determined by the competing actions of LENG8-PS and GANP-PS trimers that release mRNA from UAP56 to facilitate either RNA degradation via nuclear exosome, or export to the cytoplasm via the nuclear pore complex.
While the proposed model is attractive and may advance the understanding of polyA+ RNA fate in the nucleus, several aspects are rather speculative and require additional data to support the claims.

We appreciate the referee's constructive feedback and for identifying aspects of our work in need of additional experimental support. In the revised manuscript, we have incorporated new experiments and data analyses to consolidate our conclusion that the LENG8-PS module is both physically and functionally connected to PAXT. Moreover, we include new data, providing a rationale why the effect size of the ZFC3H1-depletion is larger than that of the LENG8-depletion. Finally, we add new cellular data substantiating that targeting of nuclear RNA by LENG8-PS occurs through the release of clamped UAP56 from the respective pA^+ RNP.

1. The authors claim that LENG8-PS constitutes a physical module of PAXT.
-There is convincing evidence to show that LENG8 and to some extent PCID2 interact with ZFC3H1 and MTR4 helicase. However, other subunits of PAXT are notably absent. Since MTR4 is not specific to PAXT and is associated with nuclear exosome, which is abundant among LENG8 and PCID2 interactants (Fig 2B), the only PAXT-specific interactant identified is ZFC3H1. The authors should provide evidence that the LENG8-PS associates with additional subunits of PAXT.

The reviewer is rightfully confounded by the nature of the PAXT connection. Much of the unclarity arises from the circumstance that PAXT is not a well-defined complex in the literature. Based on prior work, we consider ZFC3H1 and MTR4 the only *core* PAXT components, forming a tight dimer capable of contacting the nuclear exosome. This has been demonstrated in multiple studies (*e.g.* Meola et al., *Mol Cell*, 2016; Ogami et al., *Genes Dev.*, 2017). Also in the present paper, our IP of endogenously FLAG-tagged ZFC3H1 co-purified stoichiometric amounts of MTR4 but only sub-stoichiometric quantities of other proteins contributing to PAXT function (new Supplementary Fig. 7e). Indeed, this is why we originally coined PAXT a 'connection' rather than a 'complex' (Meola et al., *Mol Cell*,

2016). Our later report on an *extended* PAXT connection (Silla et al., *Nucleic Acid Res*., 2020) further underscored that PAXT, beyond its ZFC3H1-MTR4 *core*, should not be viewed as a stable entity, but rather as a composite of transiently interacting pA^+ RNP components, the depletions of which affect PAXT activity to variable extents. Such *extended* PAXT components presently include, but are likely not restricted to, the nuclear pA^+ RNA binding protein (PABPN1) and the RNA-binding proteins ZC3H3, RBM26 and RBM27. Moreover, the cap-binding complex (CBC) and its associated ARS2 protein have been reported to influence PAXT function towards some pA^+ RNAs (Polak et al., *Cell Rep*., 2023) (see also response to point 2 below).

Based on the referee's feedback we have now revised our introduction of PAXT to better explain the above considerations (revised manuscript, lines 50-51). Other parts of the paper also reflect this aspect of PAXT biology, acknowledging that common pA^+ RNP components often affect diverse nuclear processes without being firm residents of defined complexes. For example, i) polyA tail-dependent association of PABPN1 is aiding PAXT specificity towards pA^+ RNAs, but PABPN1's established productive role in RNA polyA tail formation extends way beyond that of PAXT biology (Jenal et al. *Cell*, 2012; Muniz et al., *Mol Cell*, 2015), ii) the poorly studied low-abundance protein ZC3H3 is closely associated with PABPN1 and might be involved in some PABPN1-related processes, and iii) the RNA-binding proteins RBM26 and RBM27, which both harbor UAP56-binding motifs (UBMs) (Hohmann et al., *Nature*, 2025), may promote UAP56 recruitment and likely impact both PAXT-mediated RNA decay and TREX2-dependent RNA export. Hence, we consider *extended* PAXT components RNP constituents that the ZFC3H1-MTR4 dimer may, or may not, encounter upon engaging pA^+ transcripts. To emphasize this notion, we have now discriminated between *extended*- and *core*-PAXT components by their distinct color-coding in all relevant heatmaps and scatter plots in Fig. 2 and Supplementary Fig. 7.

The referee acknowledges the robust interaction between LENG8-PS and ZFC3H1 but expresses concern that *extended* PAXT components are absent from the LENG8-3xFLAG IP in Fig. 2b. We can offer two explanations. First, based on the concept of a PAXT 'connection', one would not readily expect to find high levels of transiently interacting *extended* PAXT components. If for example the roles of RBM26 and RBM27 were to recruit UAP56 to the early pA^+ RNP, these proteins may only be present in limited amounts when the same RNP encounters LENG8-PS and the ZFC3H1-MTR4 dimer. Consistently, our ZFC3H1–3xFLAG IP returned *extended* PAXT components at low levels (new Supplementary Fig. 7c, left panel). Second, our aim of the presented IPs in Fig. 2b was to highlight main differences between LENG8–PS and GANP–PS interactors. Hence, we performed these IP experiments using high stringency washing conditions (buffer C, containing 450 mM NaCl) to enrich for high-affinity interactions, which excluded *extended* PAXT components. To better detect low-affinity interactions, we now conducted a low stringency LENG8 IP–MS experiment, matching the ZFC3H1 low stringency IP condition (new Supplementary Fig. 7f). Conversely, we conducted a high-stringency ZFC3H1 IP-MS experiment to match the LENG8 IP (new Supplementary Fig. 7d). This allowed us to compare both of these ZFC3H1- and LENG8 IPs by heatmaps displaying the mean iBAQ values of precipitating proteins within each IP subtracted by their corresponding control (new Supplementary Fig. 7e). Emphasizing known PAXT related proteins, this now illustrates: i) an over-abundance of ZFC3H1 and MTR4 (*core* PAXT) in the ZFC3H1-3xFLAG IPs and the fact that LENG8, PCID2 and exosome subunits are ~10-fold more abundant than *extended* PAXT factors; ii) high amounts of PCID2, ZFC3H1 and MTR4 in the LENG8-3xFLAG IP, but with *extended* PAXT components in much lower abundance (revised manuscript lines 188-192). We consider these results to be consistent with our model of a PAXT connection as described above. That said, we propose that the LENG8–PS complex constitutes a central functional module for a substantial subset of PAXT activity.

This interpretation is supported by the abundance of ZFC3H1 and MTR4 in the LENG8–3xFLAG IPs, along with the robust interaction detected in the reciprocal ZFC3H1–3xFLAG IPs. Together, these findings support the view that LENG8–PS represents a distinct PAXT ‘module’ rather than an *extended* PAXT factor. For completeness and transparency, IP–MS data from all experimental conditions - including fold changes, p-values, and mean iBAQ values - have now been deposited in revised Supplementary table 2.

-The authors state that interactions were tested under stringent IP conditions to enrich for high affinity interactants (line 138). The conditions used were 150 mM NaCl and 10 mM KCl together with standard concentrations of detergents. These conditions are not especially ‘stringent’. Key interactions should be tested using more stringent conditions that might support the authors’ conclusion that LENG-PS is a module of PAXT (or ZFC3H1). Similarly, although the use of engineered cell lines is indeed useful for many of the experiments, there is no demonstration of interactions between the endogenous proteins (without tags).

As explained above, we employed stringent (450 mM NaCl) conditions for the experiments shown in Fig. 2b,c to resolve any differences between high affinity GANP-, LENG8- and PCID2-interactors. Importantly, the high stringency LENG8-3xFLAG IP returned abundant levels of ZFC3H1 and MTR4 as well as exosome subunits at lower abundance (new Supplemental Fig. 7e, column 4). Instead, and as the reviewer rightfully cites, the presented ZFC3H1 IP was conducted at low stringency. To accommodate the referee’s request, we repeated the ZFC3H1 IP at high stringency and detected significant levels of LENG8 (new Supplementary Fig. 7d, left panel, and Supplementary Fig. 7e). Together with the LENG8 high-stringency IP, this supports a robust physical interaction between LENG8 and ZFC3H1.

Concerning the referee’s request for interaction analyses of untagged proteins, there is an unfortunate lack of workable antibodies, preventing us from performing IPs of endogenous LENG8, PCID2, GANP or ZFC3H1 proteins. We are, however, confident that our method of C-terminally 3xFLAG-tagging has created functional fusion proteins as all these cell lines show no discernable growth defects before adding dTAG^{V}-1 to deplete the respective fusion protein. Moreover, the ZFC3H1 and LENG8 3xFLAG-tagged constructs readily rescue molecular phenotypes of rapid ZFC3H1- and LENG8-depletion, respectively (Fig. 3a,b). Still, to substantiate the interaction data from HeLa cells, we have now conducted IP-MS experiments using HCT116 cells, carrying endogenously 2xHA-tagged ZFC3H1 or LENG8 (new Supplementary Fig. 7g). Again, no growth defects were observed unless the dTAG^{V}-1 drug was added. Agreeably, these IP-MS experiments performed at high stringency recapitulated the nuclease-resistant enrichment of LENG8, PCID2 and exosome components in the ZFC3H1-2xHA IP and the ample presence of ZFC3H1, MTR4 and exosome components in the LENG8-2xHA IP (new Supplementary Fig. 7h,i, Supplementary table 2). Finally, as an additional indication that the LENG8-ZFC3H1 interaction is direct, we now provide *in vitro* evidence, using pulldown of purified peptide variants of ZFC3H1 and LENG8, harboring wild type or mutated helical interacting regions (new Supplementary Fig. 8f).

- Supplementary Fig 3e, does depletion of ZFC3H1 alter the distribution of LENG8 in the nucleus?

This is an interesting question. In unperturbed cells, LENG8 and ZFC3H1 co-localize across the nucleoplasm without forming apparent foci or bodies (Supplementary Fig. 7j). To test for a possible ZFC3H1-dependent LENG8 distribution, in the absence of a workable LENG8 antibody, we opted to localize 3xFLAG-tagged LENG8 after siRNA-mediated ZFC3H1 depletion. No striking localization

difference of LENG8 was observed (Supporting Data below). Given this 'negative' result we suggest to not include these data, unless the referee suggests otherwise.

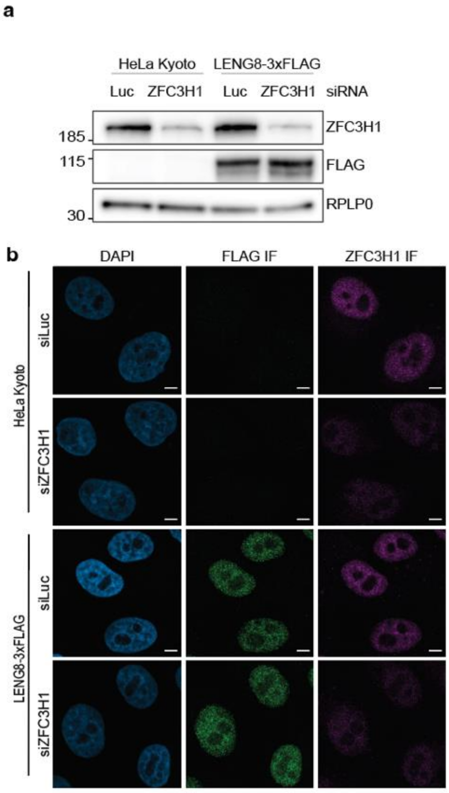

Supporting Data: Depletion of ZFC3H1 leaves LENG8 subnuclear localization unaffected. a. Western blotting analysis of cell extracts from untagged (left) or LENG8x3xFLAG (right) HeLa cells, subjected to Luc (control) or ZFC3H1 siRNA treatment as indicated. Membranes were probed with ZCF3H1-, FLAG (LENG8-3xFLAG)- and RPLP0 (control)-antibodies. b. Immunofluorescence imaging of cells from (a) probed with DAPI, FLAG- or ZFC3H1-antibodies as indicated.

In sum to this point 1 of the referee, we believe that our initial and new IP-MS analyses provide strong support that LENG8-PS constitutes an independent module of the PAXT connection. This is rationalized by the enrichment of LENG8-PS relative to *extended* PAXT components in ZFC3H1 IPs as well as by the high abundance of ZFC3H1 and MTR4 in LENG8 IPs. Orthogonal lines of evidence reinforce this notion, including nucleoplasmic co-localization of ZFC3H1 and LENG8, direct *in vitro* interaction between helical domains of the proteins, striking phenotypic concordance upon rapid depletion of the two factors, and a demonstrated requirement of their helical-domain interaction for proper *in vivo* function of both proteins.

2. LENG8-PS functions in PAXT-mediated control of nuclear polyA+ RNAs.
- The effect of LENG8 on ZFC3H1-sensitive RNAs is not compelling. In Fig 3C, if cut-offs are applied, how many RNAs are co-regulated by ZFC3H1 and LENG8?
- In Fig 3D and E, if I understand the figures correctly, the DE RNAs analysed are specific to each depletion condition separately, and not specifically those co-regulated by ZFC3H1 and LENG8. It would be instructive to analyze co-regulated RNAs. Additionally, in Fig 3d, an FC cut-off is applied, but not in Fig 3e. Indeed, very few LENG8-sensitive RNAs would pass should an FC cut-off be applied.
-in Supp Fig 5j and k, again, it would be useful to analyse the co-regulated RNAs in addition to the population by each factor individually. How many of these RNAs are co-regulated by both factors and do they share the same characteristics?

The referee raises a relevant point concerning the stronger accumulation of PAXT substrates upon ZFC3H1- *vs.* LENG8-depletion. When applying the stated fold change and adjusted p-value cut-offs in

our DESeq2 analysis, we indeed recover more targets for ZFC3H1 than for LENG8 (Fig. 3d). Importantly, however, of the set of DESeq2-defined LENG8-upregulated RNAs (n=652), almost all (n=634) are enclosed within the ZFC3H1-sensitive set (n=3916) - only with the ZFC3H1 depletion exerting a stronger effect. We now emphasize this in the text and provide data directly showing this significant overlap/co-regulation (new Supplementary Fig. 9j; revised manuscript lines 237-239). Additionally, although the ZFC3H1 depletion yields a larger number of differentially expressed RNAs, many of these are also upregulated upon LENG8 depletion – just without passing the applied cut-off. We now exemplify this by displaying substrate co-regulation applying a less stringent cut-off (new Supplementary Fig. 9j, low opacity Venn diagram in the background). In our first submission, we opted to show a correlation plot to illustrate these concordant effects between the ZFC3H1- and LENG8-depletions (Fig. 3c). With the new added data, we believe the point stands out even clearer. Finally, as requested by the referee, we have now not only analyzed RNA features for the ZFC3H1- and LENG8-sensitive transcripts (Fig. 3d,e) but also for the co-regulated (n=634) cohort (new Supplementary Fig. 9m,n,p). Reassuringly, we find similar characteristics for all these substrate classes and conclude that ZFC3H1- and LENG8-depletions yield qualitatively similar results but with different effect sizes.

We gathered that possible reasons for the difference in effect sizes could be technical (two different cell lines were generated with possible clonal effects) and/or biological (in the absence of LENG8, ZFC3H1 may still retain some ability to promote RNA turnover). To address a possible technical bias, we now performed rapid depletions of ZFC3H1 and LENG8 in HCT116 cells and conducted pA^+ RNAseq after cellular fractionation. This yielded a similarly strong co-regulation of ZFC3H1- and LENG8-sensitive transcripts, but again with the former yielding a more severe phenotype (new Supplementary Fig. 10g). Further hinting at a biological explanation, the ZFC3H1 Δ730-747 mutant, that cannot bind LENG8, still exhibited some activity in our complementation assay (Supplemental Fig. 9i), implying LENG8-independent ZFC3H1 function.

We previously reported that the ARS2 protein, which is co-transcriptionally targeted to nascent RNA by the Cap Binding Complex (CBC), can recruit ZFC3H1 through an interaction of the ARS2 effector domain with a so-called ARS2 Recruitment Motif (ARM) of the ZFC3H1 N-terminus and that this interaction can be relevant for targeting select PAXT substrates (Polak et al., *Cell Rep.*, 2023). We therefore speculated that ARS2 might target a subset of early transcripts to ZFC3H1 before UAP56-RNP complexes are readily formed. Hence, to assess whether a ZFC3H1-ARS2 interaction might contribute to the different LENG8- and ZFC3H1-depletion effect sizes, we compared ARS2- and LENG8-dependent PAXT targets by intersecting RNAseq data from a ZFC3H1 complementation assay using the ZFC3H1 ARM mutant, that cannot bind ARS2 (Polak et al., *Cell Rep.*, 2023), with the RNAseq data from the present paper obtained using the LENG8 binding-deficient ZFC3H1 Δ730-747 mutant (Supplemental Fig. 9i). Despite the different depletion methods (siRNA *vs.* dTAG) and rescue protocols (this paper *vs.* Polak et al., *Cell Rep.*, 2023), LENG8- and ARS2-dependent ZFC3H1 substrates significantly overlap (new Supplementary Fig. 9k). This is consistent with the notion that LENG8 and ARS2 can act on the same pool of ZFC3H1-regulated RNAs. We therefore suggest that, at least part of the effect size difference between LENG8- and ZFC3H1-depletion phenotypes is explained by the ability of ARS2 to 'enhance' the PAXT pathway.

In sum to this point 2 of the referee, we are grateful for the suggestion to look into the disproportional effects of the ZFC3H1- and LENG8-depletion phenotypes. By revisiting PAXT substrates using newly obtained data (HCT116 fractionated RNAseq upon rapid ZFC3H1 *vs.* LENG8 depletion as well as by comparing ZFC3H1-mutant complementation RNAseq assays), we suggest that this phenomenon is of biological significance and impacted by an ARS2-ZFC3H1 linkage, capable of suppressing RNAs in

the absence of LENG8. Although further work is required, we speculate that direct recruitment of ZFC3H1, through the CBC-ARS2 axis, will affect a fraction of early transcripts not yet properly bound by UAP56. This information has now been incorporated into the Results section accordingly (revised manuscript lines 239-246).

3. PAXT and TREX-2 contact UAP56-bound RNP to govern nuclear polyA+ RNA fate
- A central premise to the authors' model is that LENG8 can release RNAs bound by UAP56 for degradation by ZFC3H1 and nuclear exosome. However, very little evidence for this is demonstrated in vivo. For example, in the experiment shown in Supplementary Fig 7a, it would be useful to perform RIP-seq to show that the differentially bound RNAs overlap with PAXT targets.

The referee raises an important point for which we now provide additional experimental evidence to support our hypotheses. To start, we remind the referee about the LENG8 complementation assay, using the R563A LENG8 mutant (Fig. 4a), which retains all major interactions, including with UAP56 (new Supplementary Fig. 11c), yet is unable to release UAP56 from RNA (new Fig. 1c). This mutant cannot complement LENG8 depletion, which we consider strong *in vivo* support for our model.

To provide additional support and to address the referee's question, we took the advice and generated new LENG8 iCLIP libraries, using the endogenously tagged LENG8-3xFLAG cell line (Supplementary Fig. 11e,f; Supplementary table 6). To directly test whether LENG8 engages PAXT substrates, we quantified its iCLIP coverage, normalized to transcript abundance, across three distinct classes of PAXT targets; polyadenylated non-coding RNAs (ncRNAs), PROMPTs and PTTs. As a carefully matched and also abundance-normalized control sample, we quantified LENG8 iCLIP coverage across non-polyadenylated ncRNAs, PROMPTs and PTTs, that are upregulated upon rapid depletion of the nuclear exosome subunit EXOSC3, but remain unaffected by depletion of either ZFC3H1 or LENG8 (Supplementary Figure 12 b-d). Strikingly, LENG8 showed a robust and selective enrichment across the PAXT-sensitive substrate classes (new Supplementary Fig. 11h). This strongly supports a specific role of LENG8-PS in PAXT-mediated RNA decay, rather than in general nuclear exosome function. To further reinforce the proposed role of LENG8 in targeting UAP56-bound RNAs, we compared LENG8 iCLIP coverage profiles with newly generated UAP56 iCLIP data, produced from HeLa cells using a UAP56-specific antibody (new Supplementary Fig. 11d,f). Although the majority of the UAP56 iCLIP signal mapped to exonic regions of mRNAs, consistent with its established role in mRNP maturation and export (new Supplementary Fig. 11f, Supplementary table 6), UAP56 also preferentially crosslinked to canonical PAXT substrates, including pA^{+} ncRNAs, PROMPTs, and PTTs (new Supplementary Fig. 11g). We speculate that this preference is related to the fact that pA^{-} RNA substrates of the nuclear exosome are primarily processed via the NEXT complex (Lubas et al., *Mol Cell*, 2011), whose early recruitment through the CBC likely limits the access of UAP56-recruiting factors. These new iCLIP data are presented in the revised manuscript lines 311-323.

In sum to this point 3 of the referee, we trust that these complementary datasets demonstrate the *in vivo* interaction of both LENG8 and UAP56 with PAXT substrates and provide strong support of our model that LENG8-PS serves to release pA^{+} from UAP56-bound RNPs. Additional clarifications and mechanistic considerations related to this model are provided below in our response to referee 3.

- While the suggestion that ZFC3H1 protein expression is increased by LENG8 depletion is convincing (Fig 4c, supp 5b), the converse, that LENG8 protein synthesis is increased by ZFC3H1 depletion, is not compelling. Can the effect be shown by immunoblot ?

We agree with the referee that the presented SILAC data only show a modest increase in LENG8 levels upon ZFC3H1 depletion (Supplementary Table 6). Of note, our previously published whole-cell MS data from ZFC3H1-depleted cells (using an auxin-inducible degron HeLa ZFC3H1-mAID cell line) demonstrated a similar modest increase in LENG8 protein levels (Gockert et al., *Nucleic Acid Res*., 2023). However, we remind the referee that ZFC3H1-depleted cells exhibit a general defect in nascent protein production (Supplementary Fig. 13a, note generally decreased puromycin production upon ZFC3H1 depletion; also observed in Wu, et al., *Nat. Commun*., 2024), which will counteract the predicted LENG8 upregulation. Regardless, we took the referee's advice and conducted FLAG western blotting analysis of our LENG8-FLAG cells, following ZFC3H1 siRNA-mediated depletion. With an achieved ZFC3H1 depletion efficiency of ~85%, a significant increase of LENG8-3xFLAG protein could now be detected across triplicate experiments (new Supplementary Fig. 13c,d).

Referee #2 (cryo-EM, RBPs)

Remarks to the Author :
The manuscript by Jensen, Brennecke, and Plaschka groups proposes that the nuclear RNA decay pathway mediated by PAXT complex contains a TREX-2-like module consisting of LENG8, PCID2, and SEM1 proteins that recognizes pA+ RNPs bound by UAP56 DEAD-box helicase and triggers decay, whereas the canonical TREX-2 complex consisting of GANP, PCID2, and SEM1 recognizes the same UAP56 cue at the nuclear pore to promote export of pA+ RNPs.

The work combines biochemistry, a high-resolution cryo-EM structure of a UAP56:SAC3D1:PS complex in a pre-release state, cell biology with endogenous tagging, and transcriptomics with acute degron depletions to support a unifying model of nuclear pA+ RNA fate. Conceptually, showing that decay and export share the same molecular mechanism comprising UAP56 clamping and SAC3-wedge loop-mediated release is both elegant and broadly relevant to RNA biology and nuclear transport.

The central idea of the study, that PAXT and TREX-2 deploy homologous SAC3-containing modules to act on the same UAP56 interface, biasing decay vs export, certainly appears original and novel. It extends and integrates recent advances in the TREX-2 mechanism and PAXT biology and generates a significant step forward in our understanding of mRNP fate determination in the nucleus. Prior work established TREX-2 association with the pore and UAP56 release in export, and implicated PAXT (ZFC3H1/MTR4) in nuclear retention/decay of short pA+ RNAs; this manuscript connects these mechanisms. Relevant antecedents (also cited by the authors) include TREX/TREX-2/UAP56 functions, and the PAXT/ZFC3H1 literature on nuclear retention and decay of short/intron-poor transcripts. The novelty here is the TREX-2-like module containing LENG8 within PAXT acting on UAP56, the structural pre-release state, and a competition model validated by reciprocal degron phenotypes.

The results presented here should be of immediate interest to multiple communities, including RNA processing/quality control, nuclear export, and structural biology, because they reframe export vs decay as competing outcomes of a shared UAP56-centered mechanism and provide high-resolution structural insight into the “pre-release” state.

I do not see fatal flaws. The study is strong and largely self-consistent, and the labs are leaders in their fields. The cryo-EM structural work is sound and appropriately validated.

Below, I describe major points for the authors to consider addressing to improve this work to the point where it is suitable for publication in Nature.

We thank the referee for the positive assessment of our work and for valuable suggestions on how to improve the manuscript. Based on the comments, we have incorporated substantial new data and analyses. Specifically, we have carried out additional *in vitro* experiments and determined an experimental cryo-EM 3D structure of LENG8-PS complexed with RNA-clamped UAP56, replacing previously provided AlphaFold2 predictions. We have also improved our *in vivo* LENG8 complementation assays, confirming that all constructs were expressed at levels comparable to the native protein. Additionally, newly added experiments, using human HCT116 cells, replicated major protein-protein interactions and rapid depletion effects, extending the study’s primary conclusions. Finally, we explored suggestions from the referee, including a LENG8-PS/TREX-2 co-depletion experiment and a potential link between these machineries and the nuclear polyA-binding protein PABPN1.

1) Add orthogonal structural or cross-linking evidence for UAP56:LENG8:PSM (or an endogenous cross-link map) to substantiate the direct parallel to SAC3D1/TREX-2. The authors could not obtain an interpretable map of the LENG8-containing complex due to biased particle distribution (a common and often insurmountable issue in single-particle cryo-EM). However, the study then becomes overly reliant on AlphaFold predictions. Even when validated biochemically through structure-guided analysis, this is risky, and the entire premise of the study rests on the comparison and competition of similar interfaces. More evidence is required, besides structural predictions, to support the mechanisms presented. I would also advise the authors to revisit cryo-EM and attempt adding detergents or exploit tilting to obtain sufficient representation of views for reconstruction. Have the authors considered crystallizing the LENG8:PSM? Sac3:Thp1:Sem1 complex from yeast was first structurally characterized by the Stewart/Hurt labs using crystallography. This appears to be a rigid complex, so perhaps it's worth a shot.

We thank the referee for these suggestions. To strengthen our mechanistic claims, we have now carried out additional cryo-EM analysis and biochemical experiments. Through this additional cryo-EM work, we now provide four additional sets of cryo-EM structures: i) three additional LENG8-PS cryo-EM structures: A structure of the apo LENG8-PS complex (3.5 Å resolution, replacing the AF2 prediction in former Supplementary Fig. 1a, now Supplementary Figs. 3a, 4e-i, 6a-c), LENG8-PS bound to the UAP56 NTD (4.9 Å resolution, Supplementary Fig. 4e-i, 6d,e) and a structure of LENG8-PS bound to RNA-clamped UAP56 (resolution ranging from 6-12 Å, Fig. 1e and Supplementary Fig. 4e-i; 6f,g), ii) a cryo-EM structure of the apo SAC3D1-PS complex (3.6 Å resolution, replacing the AF2 prediction in Fig. S1a, now Supplementary Figs. 3a; 4a-d;5a-c). Further, we provide new biochemical data validating the interface between UAP56 and LENG8-PS (Supplementary Fig. 2j).

The newly added structural data confirms key features and the high level of similarity to the yeast Thp3-Csn12-Sem1 apo complex (Kuang et al., *Nucleic Acid Res.*, 2023) of the homologous LENG8–PS complex. For LENG8–PS–UAP56, we found that the UAP56 NTD binds LENG8–PS the same way it binds to TREX-2^{M} or SAC3D1–PS. Further, despite the lower nominal resolution of 6.2 Å of the LENG8–PS–$UAP56^{clamped}$ complex, we could unambiguously assign the clamped state to UAP56, demonstrating that LENG8–PS can engage RNA-clamped UAP56, similar to what we observed at 2.6 Å for SAC3D1–PS–$UAP56^{clamped}$. Finally, we wish to point out that recently the laboratory of Yi Ren published a cryo-EM structure of the human LENG8–PS bound to UAP56 which however differed in a key feature (Clarke et al., *Nat. Comms.*, 2025): in their structure, UAP56 was found ADP-bound, but not clamped on RNA, mirroring the post-RNA release state we had previously described for TREX-2^{M}–UAP56 previously (Hohmann et al., *Nature*, 2025). Our new data thus provides additional structural confirmation for the proposed SAC3-domain protein mechanism (revised manuscript lines 119-123, 133-142).

2) The authors really should test the requirement for the wedge loop and PS-binding at endogenous levels in cells (eg. CRISPR knock-ins for LENG8 R563A and H712F or PCID2 depletion) and read out PAXT-substrate behavior via sequencing.

The referee raises the important concern of potential dominant effects arising from overexpression of LENG8-3xFLAG constructs in our complementation assays. We agree that the conditions used in the original manuscript (48 hours of doxycycline induction) led to elevated expression of LENG8 variants, which might have influenced the analyses. Since we were concerned that a CRISPR base-editing strategy would be associated with accumulating secondary effects and possible clonal adaptation during cell line establishment, we opted to titrate the doxycycline induction times of the existing piggyBac constructs to achieve near-endogenous expression levels of all exogenous LENG8-3xFLAG variants. To do so, the LENG8-3xFLAG cell line was used as a reference for endogenous LENG8 expression

and by calibration to this, we found that 3 hours of doxycycline induction for wild-type LENG8-3xFLAG and 6 hours induction for all mutant constructs resulted in comparable expression levels (new Supplementary 11a). Under these conditions of LENG8 variant expression, endogenous LENG8-2xHA-dTAG was depleted for 4 hours using dTAG^{V}-1 and harvested RNA was subjected to RT-qPCR analysis. Notably, these experiments recapitulated our previously provided results and showed a statistically significant rescue by wild-type LENG8-3xFLAG, whereas the F301A, R563A and UAP56-binding deficient TRR mutant variants failed to do so (new Fig. 4a). Interrogating a representative set of 7 diverse PAXT targets, we deemed that additional RNAseq data would not provide additional insight and hope the reviewer agrees.

Additionally, and in line with our previous observation (original Fig. 4a), the PCID2-binding deficient H712F LENG8 mutant rescued the LENG8 depletion phenotype (Supporting Data below), which is consistent with the ability of isolated LENG8 to unclamp UAP56 *in vitro* (original Supplemental Fig. 7f,g). For now, we have decided against including this result in the revised manuscript to have it focused on the LENG8-UAP56 axis. Considering our new *in vitro* and structural data, demonstrating the importance of PCID2–SEM1 for the specificity of TREX-2-like complexes towards UAP56 (new Supplementary Fig. 3e,f,g), we feel that its inclusion might dilute the central conclusions and potentially confuse the reader.

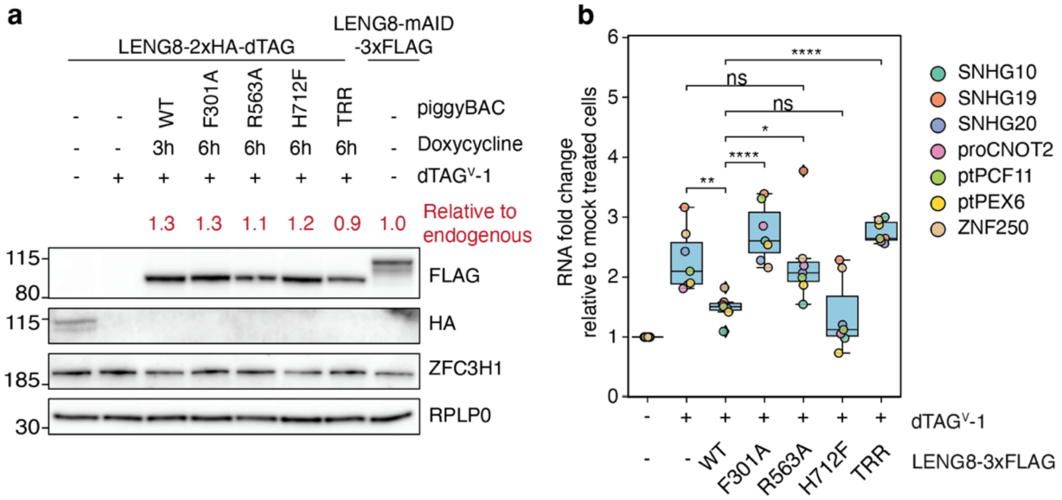

Supporting Data. Western blotting analysis and complementation assays as in Supplementary Fig. 11a and Fig. 4a, respectively, but including the H712F mutant.

3) The manuscript's central read-outs, namely the transcriptome-wide shifts in nuclear-retained vs. exported poly(A)+ RNAs and the associated fractionation profiles after acute depletion of PAXT or TREX-2 components, are demonstrated solely in HeLa cells. HeLa harbors unusually high aneuploidy, copy-number variation, and deregulated RNA-processing pathways; its responses may not fully represent those of more genomically stable human cells. I therefore encourage the authors to reproduce the key RNA-seq and nuclear-cytoplasmic fractionation read-outs in at least one additional human cell line with a more typical karyotype (for example, RPE1, HEK293, or HCT116). Confirming that the same classes of short, low-exon transcripts accumulate in the nucleus upon PAXT loss and that reciprocal effects follow TREX-2 perturbation in a second cellular background would significantly increase confidence that the proposed UAP56-centered decay/export switch is a general mechanism rather than a HeLa-specific idiosyncrasy. This replication is strongly recommended to bolster the study's impact, although I would not regard it as an absolute prerequisite for eventual acceptance.

We agree with the referee that inclusion of a parallel model system is appropriate. As also mentioned above in response to referee 1 (points 1 and 2), we therefore generated 2xHA-dTAG modified LENG8-

, GANP- and ZFC3H1-HCT116 cell lines, with which we performed IP-MS experiments as well as rapid factor depletions followed by nuclear/cytoplasm fractionated pA^{+} RNAseq. These experiments replicated our main findings from HeLa cells. First, the new high stringency IP-MS data returned i) LENG8-2xHA: LENG8-PS, ZFC3H1, MTR4 and exosome components; ii) ZFC3H1-2xHA: the ZFC3H1-MTR4 dimer as well as significantly enriched LENG8-PS and exosome components (new Supplementary Fig. 7g,h,i); iii) GANP-2xHA-dTAG: TREX-2 components (Supplementary table 2). Second, LENG8- and ZFC3H1-depletions in HCT116 cells (new Supplementary Fig. 10e) resulted in the 'HeLa-like' nuclear and cytoplasmic accumulation of mono-exonic and low-exon content RNAs (compare **c**, left part and **g** panels in the new Supplementary Fig. 10). Third, depletion of GANP in HCT116 cells mimicked the same HeLa cell experiment (Fig. 4b, right panel) by showing similar cohorts of low-exon content transcripts being downregulated in both cellular compartments and nuclear upregulation of multi-exonic mRNAs (new Supplementary Fig. 11l). Taken together, these complementary HCT116 results have now allowed us to dismiss potential technical biases linked to HeLa cell usage.

4) Having read the study several times, I would argue that the authors should clarify the decay-versus-export competition with a focused epistasis experiment. The competition model puts forward that a single UAP56 clamp on nascent pA+ RNPs can be captured by either the PAXT module (via LENG8:PCID2:SEM1) to route the RNA toward nuclear decay, or by the TREX-2 module (via GANP:PCID2:SEM1) to deliver it to the pore for export. Depleting LENG8 alone shifts short, intron-poor transcripts into the cytoplasm, while depleting GANP alone traps them in the nucleus. These two opposite phenotypes imply mutually exclusive engagement. To test this head-to-head in a single experiment, I suggest an epistasis assay in which both LENG8 and GANP are perturbed simultaneously and the fate of well-characterized reporter transcripts is monitored. For this, the authors can make use of their existing dTAG or auxin-inducible degron alleles for LENG8 and GANP to achieve synchronous, near-complete depletion within 2 to 4 hours. Alternatively, they could combine highly efficient siRNA pools (or CRISPRi guides) and harvest at the same time-point used for the individual knock-downs to control for indirect effects. Nuclear-cytoplasmic fractionation followed by RT-qPCR or short-read RNA-seq to quantify (i) classic PAXT substrates such as PROMPTs, snRNA-adjacent transcripts, or intron-poor lincRNAs; and (ii) canonical export substrates of comparable length as internal controls. smRNA FISH at a handful of loci, such as NEAT1_1, HIST1H1C, and a well-behaved short mRNA, can then visualize retention versus export. A UAP56 co-IP after double depletion can then test whether neither module can stably occupy the helicase, as predicted. If LENG8 and GANP truly compete for the same UAP56-bound RNPs, removing both should negatively impact both decay and export pathways. Transcripts (a few examples suggested above) would accumulate as unprocessed nuclear pA+ species, potentially yielding larger nuclear retention than either single knockdown. However, if the modules act on different RNP pools, the double knock-down should produce an additive or intermediate phenotype, not a synergistic block. Because the single-factor degron data already point strongly to competition, I do not suggest that this double-perturbation experiment is strictly essential for acceptance; however, even a small-scale test at a few loci would provide direct functional evidence that the two SAC3-domain assemblies are mutually exclusive gatekeepers of UAP56 and, in the opinion of this reviewer, would significantly strengthen the paper's central mechanistic claim.

The referee suggests a double depletion experiment to directly test whether dual GANP/LENG8 depletion would further accumulate PAXT-substrates in the nucleus. To investigate this, we attempted to generate cell lines to achieve the synchronous rapid depletion of LENG8 and GANP by combining the respective dTAG constructs. Despite multiple attempts we were (only) able to achieve this in the HCT116 background and here the resulting cell line appeared extremely growth impaired upon addition of $dTAG^{V}$-1. Even so, we proceeded with analysis and, notably, observed the strong downregulation of

exon-intron borders of several pre-mRNAs, which were otherwise insensitive to individual LENG8- or GANP-depletion (Supporting Data a,b below). We take this to imply that dual LENG8/GANP depletion induces a rapid transcriptional shut down, which is in line with the severe growth impairment of these cells. Regrettably, this prevented the intended analyses.

Beyond our unsuccessful attempts in generating dual LENG8/GANP-dTAG HeLa cells, we were indeed able to generate a ZFC3H1/GANP double dTAG line, which again grew poorly upon addition of dTAG^{V}-1. Still, we subjected these cells to fractionated RNAseq, but as with the double LENG8/GANP depletion of HCT116 cells, double depletion of ZFC3H1 and GANP resulted in transcriptional impairment as measured through the global decrease of intron counts in the nuclear RNA fraction (Supporting Data c,d below).

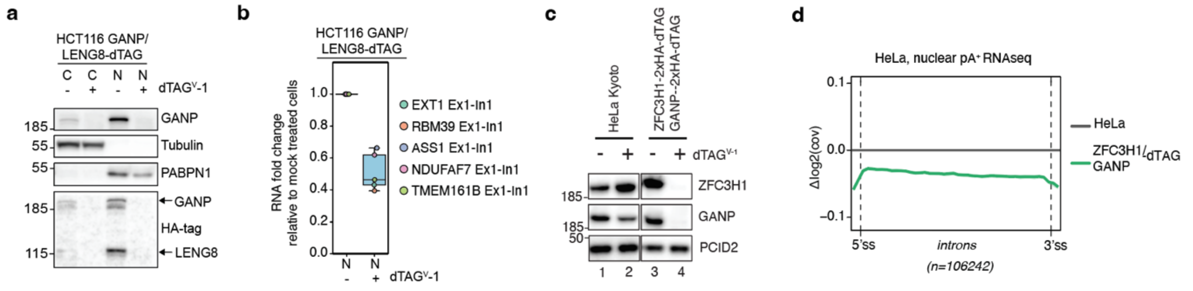

Supporting Data. a. Western blotting analysis of cytoplasmic (C) and nuclear (N) fractions of HCT116 GANP/LENG8-2xHA-dTAG cells treated with dTAG^{V}-1 (+), or not (-), for 4 hours. Membranes were probed with GANP, tubulin, PABPN1, and HA-tag Abs as indicated. b. Boxplots of RT-qPCR analysis of selected nascent transcripts (employing amplicons targeting exon-intron junctions). These targets were non-affected by individual GANP- or LENG8-depletion. c. Western blotting analysis as in **a**, but for whole cell extracts of maternal HeLa- or ZFC3H1/GANP-2xHA-dTAG cell lines, treated, or not, with dTAG^{V}-1 as indicated. Membranes were probed with GANP, ZFC3H1 and PCID2 Abs as indicated. d. Aggregate plot displaying nuclear pA^{+} RNA coverage changes across introns upon dTAG^{V}-1 treatment (4 hours) of ZFC3H1/GANP-2xHA-dTAG cells relative to the HeLa control.

We can only speculate why we have this difficulty of combining LENG8 (ZFC3H1)- and GANP-depletions and why resulting cells are severely transcriptionally challenged. We consider that it might reflect their inability to recycle UAP56. The LENG8-PS and GANP-PS modules do not only compete for RNPs but are also redundant in their abilities to release UAP56 from RNP complexes. When depleting LENG8, RNPs can still be engaged by GANP-PS and *vice versa* when depleting GANP, LENG8-PS will recycle UAP56. In both cases nuclear UAP56 turnover might allow for new RNA to be compacted into RNPs. Consistent with this possibility, the FLAG-UAP56 M1 mutant (Hohmann et al., *Nature*, 2025) was incapable of interacting with either LENG8 or GANP but instead trapped UAP56 in a post-THO complex state, accumulating RNP components. We speculate that this imbalance might provoke the observed transcriptional shutdown.

5) Finally, the authors should test the model in the background of acute PABPN1 depletion to determine whether the proposed UAP56:LENG8 (PAXT) vs TREX-2 competition depends on protected, PABPN1-coated poly(A) tails. PABPN1 binds and organizes nuclear poly(A) tails, promotes canonical extension by PAP, and limits premature deadenylation; its loss yields shortened/heterogeneous tails and greater 3′-end exposure. If the mechanism preferentially engages well-protected pA+ RNPs, PABPN1 loss should diminish PAXT engagement or shift export/decay balance; conversely, if oligo-adenylated/poorly protected 3′ ends favor PAXT capture, depletion should promote and accentuate PAXT phenotypes. Concretely: perform dTAG/siRNA PABPN1 +/- LENG8 or ZFC3H1 (and/or GANP) perturbations; quantify nuclear/cytoplasmic distribution and decay rates for sentinel short/intron-poor transcripts; and

profile poly(A) tail lengths (e.g., PAL-seq/TAIL-seq or long-read tail-length calling). Co-IP of UAP56 with PAXT vs TREX-2 under PABPN1 depletion would further test whether 3′-end protection modulates complex choice. This experiment seems quite essential, and PABPN1 is presently somewhat neglected in the current version of the manuscript, given its centrality in 3′ end processing.

We thank the referee for raising this point. Indeed, in the original manuscript, we did not directly investigate 3′ end processing or the role of poly(A)-binding proteins (PABPs) in nuclear RNA sorting. Although, we consider this a relevant question, it is also a very complex one. The presence of multiple nuclear PABPs, including PABPN1, ZC3H14, and ZC3H11A, with partially overlapping functions in RNA polyadenylation and possibly decay (e.g. Latour et al., *Nucleic Acids Res*., 2025; Insco et al., *Science*, 2023), together with the evidence for nucleo-cytoplasmic shuttling of cytoplasmic PABPs, PABPC1 and PABPC4, raises the likelihood of redundancy, complicating investigations of 'easy' relationships between PABPs, transcript 3′ end formation and downstream RNA metabolic events. In this light, we respectfully suggest that a comprehensive analysis is beyond the scope of the present work. That said, we agree that the question of whether depletion of PABPN1 protects PAXT substrates from nuclear degradation, or conversely enhances their decay, is interesting. We note here that our original study, identifying PAXT as an exosome adaptor for polyadenylated nuclear RNAs, established PABPN1 to be actively involved in PAXT function (Meola et al., *Mol Cell*, 2016). Consistent with this, multiple studies have since shown that depletion of PABPN1 - using either siRNA- or rapid-depletion approaches – provokes the accumulation of PAXT substrates (*e.g.* Silla et al., *Nucleic Acid Res*., 2020; Huang et al., *EMBO Rep*., 2023). Additionally, recent work has further characterized PABPN1 as a nuclear retention factor, with its depletion resulting in the leakage of polyadenylated nuclear transcripts into the cytoplasm (Kwiatek et al., *RNA*, 2023).

With the above considerations in mind, we therefore performed co-depletion experiments combining siRNA- and dTAG-mediated depletions of PABPN1 and LENG8, respectively (Supporting Data a below). In agreement with the present paper and published studies, depletion of either PABPN1 or LENG8 led to the accumulation of selected PAXT substrates in both nuclear and cytoplasmic fractions of HeLa cells (Supporting Data b below). However, PABPN1/LENG8 co-depletion did not result in a further increase beyond that observed in single depletion samples. We interpret these findings to indicate that PABPN1 and LENG8 operate sequentially in PAXT function. A possible interpretation could be that PABPN1 is required for PAXT to identify target RNA 3′ ends after their LENG8-PS-mediated release from UAP56-bound RNPs. Notably, the observed cytoplasmic accumulation of PAXT targets implies that in the absence of PABPN1, nuclear transcripts can be exported. Whether these RNAs have undergone aberrant polyadenylation, but despite this are capable of nuclear export, perhaps by associating with other PABPs, remains an open question. We are doubtful, however, that measurements of polyA-tail lengths in these conditions will provide sufficient new insight without more dedicated research on PABPN1 and the other nuclear PABPs.

For now, we have chosen not to include these data in the manuscript, as in our view it does not provide substantial additional insight beyond current knowledge. However, we are happy to reconsider if the referee deems that its inclusion would strengthen the manuscript.

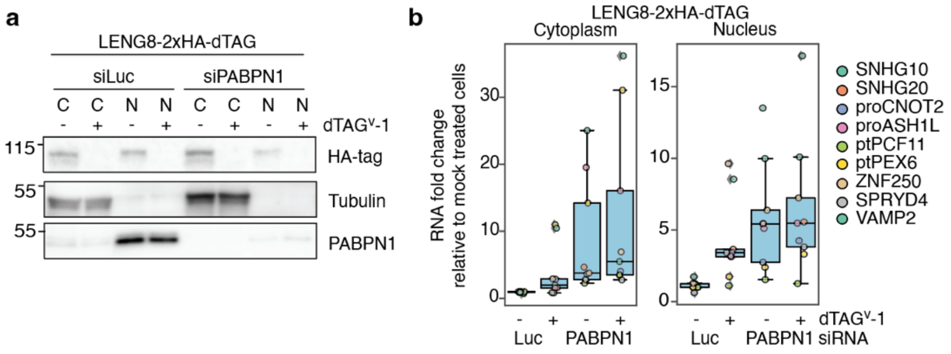

Supporting Data. PABPN1 and LENG8 both operate in the PAXT pathway. a. Western blotting analysis of cytoplasmic (C) and nuclear (N) fractions of HeLa LENG8-2xHA-dTAG cells treated with siRNAs to luciferase (siLuc) or PABPN1 (siPABPN1) for 48 hours and/or treated with dTAG^{V}-1 for 4 hours (+) or not (-). Membranes were probed with HA-, tubulin- and PABPN1-Abs as indicated. b. Boxplots of RT-qPCR analysis of selected PAXT substrates from nuclear or cytoplasmic fractions of biochemically fractionated HeLa LENG8-2xHA-dTAG cells subjected to dTAG^{V}-1-induced depletion of LENG8 and siRNA-induced depletion of PABPN1 as in **a**. RNA fold changes were calculated relative to levels in non-treated controls and normalized to levels of GAPDH mRNA, separately for cytoplasmic (left) and nuclear (right) fractions.

Minor points are described below.

a) Whilst I appreciate the beauty and aesthetics of structural figures, the thickness of the cartoons representing secondary elements obscures relevant residues and makes interfaces difficult to discern. This concerns figures 1h, 2e, S1a (do all subunits need to be various shades of blue?). Please consider including more views in the SI. I would emphasize that the clarity and contrast in structural figures are more important than aesthetics.

Clarity is indeed imperative in structural figures. We have therefore taken the referee's suggestions and i) reduced the thickness of the cartoons in the structure figures, ii) included more labelling in Fig. 2e and iii) provided additional views in Supplemental Fig. 5,6. Our color code was chosen for consistency with other papers, so changing it now would cause problems for accessibility and pair-wise comparisons among publications.

b) In biochemical pulldown experiments, quite often the msot obvious explanation of the loss of binding can be a trivial one such as poor stability of a truncated construct or buffer conditions. The authors should consider their controls more carefully.

We agree with the referee about this possible concern. In all our pulldown experiments, we therefore included a positive control to ensure that the experimental conditions (buffer composition, incubation time, number of washes) in general support the interaction interrogated in the respective experiment. We have in addition validated interactions probed by pulldowns, using an orthogonal method (Grating Coupled Interferometry) to ensure that the observed effects are not pulldown artefacts. That said, we now also included analytical size exclusion chromatography traces with Coomassie-stained SDS-PAGE gels for peak fractions for all used recombinant proteins (new Supplementary Fig. 1). These confirm that proteins used in our biochemical experiments were well-behaved (migrating near their expected molecular weights), indicating properly folding and absence of truncations.

c) Lines 118-119: "...pre-positions R102 adjacent to F381… priming it to displace this residue…" Aside from a lack of clarity in what is priming or displacing what, there is essentially no evidence to support this structural claim. Nevertheless, it is quite central to the entire mechanism. How can one residue 'prime' the displacement of a neighboring one? Please either support with evidence or mark this statement as speculative.

We apologize for not being clearer on this aspect. We found the central wedge loop arginine, R678 in GANP, to stack onto the base of the bound nucleotide in the post-RNA release state observed previously in our TREX2^{M}–UAP56 structure (Hohmann et al, *Nature*, 2025). In contrast, in the high-resolution structure of the pre-release state for SAC3D1–PS–UAP56 reported here, we found that the equivalent wedge loop R in SAC3D1, R102, is forming a hydrogen bond with the UAP56 residue E354. This interaction allows the wedge loop to already engage UAP56 in the clamped state and positions SAC3D1 R102 in close proximity to the nucleotide binding site, potentially enabling the subsequent conformational change where SAC3D1 R102 displaces UAP56 F381 in the nucleotide binding site, ultimately enabling ADP, Pi, and RNA release. We have now adjusted the text to provide further detail on this mechanism. As we can currently not with certainty assign the order of events between 'wedge loop engaged with clamped UAP56' and 'wedge loop engaged with post-release UAP56', we phrased this as a speculative statement.

d) The authors lean rather heavily into AlphaFold-based structure predictions, but, in my view, these require quite a bit more work to have confidence than simply including PAE plots, which are rather uninformative for interfaces. For key predictions, the authors should generate 25 independent predictions and assess their agreement by superposition.

Additional cryo-EM data provided in the revised version of this manuscript allowed us to replace the AF2-predicted structures of the apo complexes of LENG8–PS and SAC3D1–PS. This leaves the LENG8–ZFC3H1 interaction (Fig. 2e) the only remaining AF2 prediction in the manuscript for which we do not provide an experimental structure. Following the referee's suggestion, we have now run the LENG8–ZFC3H1 complex in 25 independent predictions (new Supplementary Fig. 8e). We found that the LENG8–ZFC3H1 interface was predicted in 25/25 predictions, and the resulting predicted structures were virtually indistinguishable with RMSDs ranging from 0.16 Å to 1.1 Å (Supplementary Fig. 8e). We now also provide biochemical validation for the predicted LENG8–ZFC3H1 complex. For this experiment, we purified the wildtype or mutant ZFC3H1-binding domain of LENG8 (residues 283-346) and probed its interaction with a minimal wildtype or mutant ZFC3H1 synthetic peptide (new Supplementary Fig. 8f). In line with the AF2 prediction, this *in vitro* pulldown shows that wildtype LENG8 and ZFC3H1 interact *in vitro*, while point mutations in either interaction partner abolished complex formation, supporting our cellular results.

e) Lines 165-166: "...a conserved motif of two alpha-helices..." Structural comparisons should be supported by structural predictions rather than sequence alignments.

We thank the referee for this suggestion. We now displayed the superposition of the ZFC3H1-interacting domain of LENG8 from selected model species in new Supplementary Fig. 8d. We found that these domains show a high degree of structural similarity, with RMSDs ranging from 0.35 to 0.96 Å. The sequence alignment in addition showed that key ZFC3H1 interacting residues are conserved between species. We thus prefer to show the new structural comparison in addition to the existing sequence alignment (new Supplementary Fig. 8c,d).

f) Line 268: "...ensure specificity for UAP56," Here, the authors should test more than just EIF4A3 to make the case for specificity for UAP56.

We thank the reviewer for the comment, based on which we have made several revisions. First, we have clarified that, based on the available data, only UAP56 (DDX39A/B) is known to bind SAC3-domain containing complexes, but not other DExD-box ATPases: Only UAP56 harbors the N-terminal domain (NTD) to specifically bind the three SAC3-domain containing complexes (Hohmann et al., *Nature*

2025; and revised manuscript lines 127-133). In addition, only UAP56 is predicted to bind LENG8-PS in two available proteome-wide AlphaFold Multimer screens (Schmid EW, Walter JC, *Mol Cell*, 2025; Zhang, Humphreys et al. *Science*, 2025). Further, the available cellular biochemical data suggest that the SAC3-domain containing proteins (GANP, LENG8, SAC3D1) exist only as part of PCID2-SEM1 assemblies, but not in isolation. This is important, since this would preclude SAC3-containing complexes from acting, for example on EIF4A3 (see new Supplementary Fig. 3e,f,g). We thus apologize for giving the impression that specificity of SAC3-domain complexes to UAP56 or other DExD-box ATPases is in question. We simply pointed out the specificity to address the technical question whether isolated LENG8 (which does not occur in cells) has altered specificity *in vitro* (former Supplementary Fig. 7f,g).

We have therefore not included additional DExD-box ATPases in this assay. We have, however, made the following other additions: i) we now include a pulldown assay to demonstrate that LENG8-PS interacts with UAP56, but does not bind the DExD-box proteins EIF4A3 or DDX19 *in vitro* (new Supplementary Fig. 3f), ii) we simplified the ATPase assay in the original Supplementary Fig. 7f,g to limit the data for LENG8-PS (*i.e.* the cellularly meaningful assembly) *vs.* UAP56 or EIF4A3 (now Supplementary Fig. 3g) and iii) we adapted the main text discussing these experiments (revised manuscript lines 147-151). We hope the reviewer agrees with these changes and clarifications.

g) Lines 288-289: "...due to overloading of ribosome by ncRNAs escaping from the nucleus." The authors should clarify here how and in what circumstances ribosomes can become overloaded by non-coding RNAs. Are these capped?

This is an interesting and complex question. Multiple studies have shown that translational defects arise when abundant amounts of ncRNAs accumulate in the cytoplasm (Ogami et al., *Genes Dev*., 2017; Wu et al., *Nat. Commun*., 2024). One plausible mechanism is ribosome exhaustion caused by competition between capped ncRNAs and mRNAs, particularly given evidence that certain ncRNAs, such as PTTs, can be translated in eukaryotic cells (*e.g.* Insco et al., *Science*, 2023). However, we cannot exclude alternative mechanisms by which cytoplasmic ncRNAs may impair mRNA translation, including competition for cytoplasmic RNA-binding proteins or activation of cellular stress-response pathways. Regarding the capping status, we note that the majority, it not all, RNAs targeted by PAXT are capped as this occurs very early during transcription initiation by RNA polymerase II. Consistent with this, our own previous data, including CAGE-seq analyses (Lykke-Andersen et al., *Mol. Cell*, 2021) and iCLIP studies of the CBC (Cordiner et al., *Nat. Commun*., 2023), demonstrated the capping of PROMPTs and other PAXT-targeted ncRNAs.

Referee #3 (Remarks to the Author):

In this manuscript, Bugai et al. describe two TREX-2-like modules—LENG8-PS and SAC3D1-PS—and propose that LENG8-PS competes with TREX-2 for binding to the export factor UAP56, thereby regulating nuclear retention and decay of typically short polyadenylated RNA species, which often result from spurious transcription (PROMPTs) or premature cleavage and polyadenylation (ptRNAs).
The authors were unable to solve the 3D cryoEM structure of UAP56-bound LENG8-PSM (in which only the SAC3 domain of LENG8 is included). However, they provided evidence of its cryoEM structure in 2D and AlphaFold predictions that are consistent with a cryoEM structure of UAP56-bound SAC3D1-PSM, which they successfully solved at 2.6 to 4.5 Å resolution. Combined with additional AlphaFold predictions with the core PAXT factor ZCF3H1, the authors generated multiple LENG8 variants defective in (i) activating UAP56 ATPase activity (i.e., LENG8(R563A)), (ii) binding PCID2 (i.e., LENG8(H712F)), and (iii) binding ZFC3H1 (i.e., LENG8(F301A)). They also generated a ZFC3H1 deletion variant unable to bind LENG8, i.e., ZFC3H1(Δ730-747). The authors then combined the use of these variants with rapid depletion of endogenously tagged proteins to nicely demonstrate how the LENG8-ZFC3H1 interaction is key to mediating the degradation of PAXT targets transcriptome-wide. This aspect of the manuscript is overall solid. The authors then use similar tools to propose that PAXT-mediated RNA decay is mediated by UAP56. However, we are unconvinced that the experiments provided here support this claim (see major comment #1). The authors also unveiled interesting feedback loops between LENG8 and ZFC3H1 that likely function to maintain PAXT activity when one or the other protein is depleted.
This manuscript combines numerous state-of-the-art approaches to enrich our understanding of how PAXT is (or could be) recruited onto target RNAs and, more importantly in our opinion, how nuclear RNA decay competes with nuclear RNA export. While most experiments are well conducted and interpreted, we identified flaws or uncertainties that need to be addressed.

We thank the referee for the overall positive assessment of our work and the critique questioning the centrality of UAP56 in PAXT-mediated RNA decay. Based on the comments, we have focused on providing additional experimental support for our model that LENG8-PS interacts with RNP-bound UAP56 to release it from pA^+ RNA both *in vitro* and *in vivo*. We believe this has been achieved and has substantially served to strengthen the conclusions of the study.

Major comments

1) UAP56 can be recruited onto RNPs via ALYREF, which can be recruited via the EJC in the case of spliced RNPs, via RBM33 in the case of single-exon mRNAs with a 5'-GC-rich region (Thomas et al., Genes&Dev. 2022), or via SLBP in the case of replication-dependent histone mRNAs (Fan et al., EMBO J. 2019). However, it is unclear if and how UAP56 is recruited onto the RNA species that are typically degraded by PAXT/LENG8, i.e. PROMPTs and ptRNAs.

The referee raised the important point that if UAP56 is to have any impact on nuclear pA^+ RNA decay, the protein has to be recruited to the affected pA^+ RNPs. As the referee also mentions, the best studied mediator of UAP56 recruitment is the ubiquitous RNA binding protein ALYREF. Indeed, we consider it likely that ALYREF can be involved in recruiting UAP56 to PAXT targets. This is because ALYREF is well-positioned to do so via its interaction with the Cap Binding Complex (CBC) situated at the 5′ cap of all RNA polymerase II-derived transcripts, including mono-exonic RNAs, PROMPTs, eRNAs and PTTs (Shi et al., *Nucleic Acid Res*., 2017; Cordiner et al., *Nat. Comm*., 2023; Clarke et al., *eLife*, 2024). However, we would also like to draw the attention of the referee to the fact that, beyond

ALYREF, multiple RNA-binding proteins contain so-called UAP56 Binding Motifs (UBMs) (Gromadzka et al., *NAR* 2016; Hohmann et al., *Nature*, 2025; Thomas et al., *Genes&Dev*. 2022), suggesting that diverse features of an RNA might enable UAP56 recruitment. Interestingly, UBM-containing proteins include CHTOP, PHAX, RBM26 and RBM27. Of these, the two latter proteins have previously been shown to interact with PAXT substrates (Fan et al. *Mol Cell*, 2024) and to impact PAXT-mediated degradation of nuclear pA^{+} RNAs (Silla et al., *Cell Rep.,* 2023). Hence, there are several candidate factors that might recruit UAP56 to target RNAs. We have now emphasized these central considerations in the Results section of the manuscript (revised manuscript lines 320-323).

To provide direct evidence addressing the referee's concern whether UAP56 is recruited to PAXT targets, we assessed UAP56-RNA binding *in vivo* using iCLIP of endogenous UAP56 (new Supplementary Fig. 11d,f,g). As also stated above in point 3 of the response to referee 1, the resulting iCLIP data demonstrated the substantial binding of UAP56 to PAXT substrates, including polyadenylated ncRNAs, PROMPTs and PTTs (new Supplementary Fig. 11g). In addition, LENG8 also binds these RNAs, which we demonstrated by new iCLIP experiments using endogenously 3xFLAG-tagged LENG8 (new Supplementary Fig. 11e,f,h). While these two new datasets provide direct evidence for recruitment of UAP56 and LENG8 to PAXT substrates, we note that the mechanisms governing UAP56 recruitment to different RNA species remain an important area for future investigation. These new iCLIP data are presented in the revised manuscript lines 311-320.

Beyond this knowledge gap, other elements make it doubtful that PAXT/LENG8 binds and degrades its RNA targets via UAP56, which seems essential to explain functional competition with TREX-2:
1a) In Fig. S7a, reduced amounts of RNAs in anti-LENG8(R563A) IPs relative to anti-LENG8(WT) IPs are accompanied by a reduced IP efficiency of LENG8(R563A) relative to LENG8(WT). Thus, it is unclear if the R563A mutation impairs the ability of LENG8 to bind RNA via UAP56 in cells. Notably, we are unsure why the authors hypothesized that this mutation, which they claim blocks the release of UAP56 from RNA in vitro (although this was not demonstrated), would reduce UAP56-mediated binding of LENG8 to RNA. Wouldn't it more straightforward to test a LENG8 variant that does not bind UAP56? Available or predicted structure determinations of TREX-2-like complexes could very likely provide the structural insights required for the design of such a variant.
Additionally, the authors should investigate the binding of LENG8 to PAXT targets rather than to total-cell RNAs, especially if, as suggested by data in Fig. S7f,g, LENG8 also binds EIF4A3, which is usually deposited on spliced mRNAs as an EJC constituent.

We thank the referee for these comments, which we have addressed below.

First, we quantified the results in a new Supplementary Fig. 11b (old Supplementary Fig. 7a), which recapitulated a decrease in the RNA-crosslinking efficiency of the R563A mutant. Our interpretation of this result is that LENG8 is less likely to crosslink to RNA if LENG8 cannot engage the UAP56 RecA1-A2 core with the clamped RNA. If the LENG8 wedge loop is mutated (R563A), LENG8 will still contact UAP56 via the interaction with the UAP56 NTD; but due to the flexible linker between the UAP56 NTD and the clamped core (RecA1-RNA-RecA2) LENG8 will not come in close enough proximity to the RNA to enable efficient crosslinking. Related to this, and rightfully pointed out by the referee, our initial manuscript did not provide sufficient proof that the R563A mutant is defective in RNA release. In the new Fig. 1c,d, we now demonstrate that wedge loop mutants of both LENG8 and SAC3D1 fail to release clamped UAP56 from RNA *in vitro*. This, together with newly published, and similar, data for the GANP-PS module of TREX-2 (Hohmann et al., *Nature*, 2025), provides a clear functional homology of the three TREX-2-like complexes in their abilities to release UAP56 from RNA.

Taken together, we consider the correlating inabilities of the R563A mutant to 'wedge' open UAP56 to release RNA *in vitro* (Fig. 1c) and to complement LENG8 depletion *in vivo* (Fig. 4a) as strong support for the involvement of UAP56 in PAXT-mediated RNA decay.

Second, to address the referee's comment to investigate a LENG8 variant that does not bind UAP56, we employed a structure-guided approach to locate residues in different parts of LENG8, the mutations of which would predictably affect UAP56 interaction. Implanting these triple (T645W, R749D, R791D; 'TRR') changes into a LENG8 variant constructs markedly decreased the interaction of LENG8-PS^M with UAP56 *in vitro* (new Supplementary Fig. 2j) making it uncapable to stimulate UAP56 ATPase activity (new Supplementary Fig. 2h, lane 7) and release UAP56 from RNA (new Supplementary Fig. 2k, lane 8). To assay whether the UAP56-interacting mutants would complement LENG8 function *in vivo*, we expressed this LENG8-TRR mutant in LENG8-2xHA-dTAG cells upon depletion of endogenous LENG8 (Supplementary Fig. 11a). Like the R563A mutant, the LENG8-TRR was incapable of rescuing the phenotype of LENG8 loss (Fig. 4a). This therefore provides orthogonal evidence that UAP56 is central for PAXT activity.

Third, to address directly whether LENG8 binds to PAXT targets, we took the referee's advice and produced new LENG8 iCLIP data (as also mentioned to referee 1 in point 3 above). These results are now presented in new Supplementary Fig. 11d-h and demonstrate that both LENG8 and UAP56 bind PAXT substrates directly. Hence, both factors are loaded onto spliced transcripts as well as onto mono-exonic PROMPTs, PTTs and other ncRNAs consistent with our proposed model.

Lastly, we wish to clarify that LENG8 does not bind EIF4A3 *in vitro*. In the original Supplementary Fig. 7g we did not show an *in vitro* pulldown experiment. This panel instead showed the composition of the samples (reaction mixture) used for the ATPase assay in the original Supplementary Fig. 7f, which revealed that the LENG8–PS complex stimulates the apparent ATPase rate of UAP56, but not the ATPase rate of EIF4A3. We now: i) simplified the ATPase assays and their discussion by showing only experiments with the full LENG8–PS complex (new Supplementary Fig. 3g; revised manuscript lines 149-151) and ii) provided clearer labelling and show gels and ATPase experiments always side-by-side in one figure to improve the interpretability of these experiments. Finally, we now also provided an *in vitro* pulldown experiment, showing that indeed LENG8–PS does not bind EIF4A3 or the closely related DExD-box protein DDX19 (new Supplementary Fig. 3f).

1b) Figs. S4i and S7b indicate that the R563A mutation reduces the association of LENG8 with the RNA exosome and possibly with MTR4 (but not with ZCF3H1). Thus, we are unconvinced that the "release of RNA from UAP56 is central for its PAXT-mediated turnover". Additionally, the authors should explain how UAP56 ATPase activity is linked to its release from RNA, and why they think that release of UAP56 from RNA would be required for exosome-mediated RNA decay.

We wish to clarify an apparent misunderstanding that the IP-MS analysis of the LENG8(R563A) mutant displayed reduced levels of MTR4 and nuclear exosome subunits. In the Chymotrypsin-digested samples of new Supplementary Fig. 8i, the differences between the wild type LENG8 (first row) and the LENG(R563A) mutant (last row) were non-significant. Indeed, this was also the case when comparing Trypsin-digested samples in the original Supplementary Fig. 7b (now Supplementary Fig. 11c). We believe that the reason for the misunderstanding might be that we, in the initial submission, opted to not normalize protein interactant- to bait-levels between wild type and mutant IPs (note that LENG8 was not on the '0' mid-line). Regrettably, this presumably led to the impression that exosome subunits were downregulated in the LENG8(R563A) IP. To remedy this, we have now updated all

comparative IP-MS data to be displayed in normalized fashions, yielding better estimations of possible mutant effects. This can now be exemplified by the LENG8(R563A) *vs.* LENG8 wild type IP, where the R563A mutant precipitates relatively more UAP56 while exosome subunit changes are largely non-existing or non-significant (new Supplementary Fig. 11c).

The referee also wonders how UAP56 ATPase activity might be linked to its release from RNA, and why this would be required for PAXT-mediated RNA decay. In our conducted ATPase assays, the measured readout is the apparent ATPase rate. Importantly, this readout does not resolve which step of the ATPase cycle is being stimulated. The ATPase cycle of UAP56 comprises: i) binding of ATP and RNA, ii) ATP hydrolysis and iii) release of ADP, inorganic phosphate, and RNA. Thus, an increased ATPase rate cannot be directly assigned to enhanced RNA release without additional mechanistic context. UAP56 forms highly stable 'clamped' complexes with RNA, that can persist for more than 30 min at room temperature (Hohmann et al., *Nature*, 2025). In these complexes, ATP is hydrolyzed immediately upon RNA binding, such that UAP56 predominantly exists in an ADP·Pi-bound state rather than an ATP-bound state (Hohmann et al., *Nature*, 2025). Consequently, the apparent ATPase activity of UAP56 in the presence of RNA is low, because the enzyme remains associated with the same RNA molecule for prolonged periods. The rate-limiting step in the disassembly of the UAP56–RNA complex is the release of ADP and Pi from UAP56. ATPase activity and RNA release are therefore indirectly linked. TREX-2 and the related complexes described in the present study weaken the interaction between UAP56 and the bound nucleotide, thereby promoting release of both nucleotide and RNA. This release allows UAP56 to re-enter a new ATP-binding cycle, forming a new UAP56–ATP–RNA complex in which ATP is again rapidly hydrolyzed, regenerating the stable UAP56–ADP·Pi–RNA state. We hope that this explanation satisfies the referee and would be happy to include aspects in the paper upon request. Finally, we would like to clarify that we do not propose that release of UAP56 from RNA is directly required for exosome-mediated RNA decay. Instead, we suggest that UAP56 release is necessary to prevent the RNA from engaging TREX-2 and being exported. In this model, UAP56 removal indirectly favors nuclear RNA retention and decay by blocking productive recruitment of the export machinery, rather than by actively promoting exosome function.

1c) While the authors convincingly demonstrated the association in cells of ZFC3H1 with LENG8 and PCID2, they did not show that LENG8 (as part of LENG8-PS module), bridges ZFC3H1 to UAP56. Supporting this model, data from Table S2 indicate that UAP56/DDXD39B co-immunoprecipitates with ZFC3H1(WT) and less efficiently with ZFC3H1(Δ730-747). However, the authors did not investigate the significance of this result and did not test if the co-IP was resistant to RNase. As was shown for the ZFC3H1-binding-defective variant of LENG8 (i.e., LENG8(F301A)), it seems important to demonstrate that a UAP56-binding-deficient variant of LENG8 fails to support the decay of PAXT targets.

The referee is correct that the originally presented IP samples of wild type and Δ730-747 mutant ZFC3H1 were not conducted in the presence of nuclease, which prevented any interpretation of whether LENG8-PS would bridge UAP56 to ZFC3H1. To address this comment, we now also performed ZFC3H1 variant IP-MS experiments in nuclease-containing conditions. While the direct binding of LENG8 to ZFC3H1 was resistant to nuclease treatment, a partial loss of UAP56 and other RNA-binding proteins, like PABPN1, was observed (new Supplemental Fig. 7c-d, right panels; and Supplemental Table 2). We surmise that this loss reflects that multiple UAP56 molecules are often present in the same RNP but that most of these are not directly targeted by the LENG8-PS module. More importantly, comparing our new nuclease treated IP-MS samples between wild type and mutant ZFC3H1 revealed the significant loss of both LENG8-PS and UAP56 in the Δ730-747 mutant IP - while MTR4 and

exosome subunits were unaffected (new Supplementary Fig. 8h). This reinforces the notion made by the referee and confirms that LENG8-PS mediates an interaction between ZFC3H1 and UAP56. In further support of our model, we also now show that the UAP56-binding deficient TRR mutant of LENG8 fails to rescue the accumulation of PAXT targets; similarly to the ZFC3H1-binding and UAP56-unclamping LENG8 mutants (Fig. 4a, see also above response to point 1a). Finally, our new iCLIP data of LENG8 and UAP56 (new Supplementary Fig. 11d-h) demonstrated direct and specific *in vivo* binding of both proteins to PAXT substrates, providing an additional angle of support for LENG8-ZFC3H1 substrate targeting via UAP56.

1d) Table S2 indicates that EIF4A3 (and other EJC constituents) as well as other RNA helicases that may share amino-acid similarity with UAP56 are also enriched in anti-ZFC3H1(WT) IPs relative to ZFC3H1(Δ730-747) IPs. This raises the possibility that LENG8 recruits PAXT onto target RNAs via different RNA helicases, the identity of which may depend on the RNA species. However, focusing on UAP56 would be sufficient if the authors can demonstrate UAP56 is key to mediate RNA decay by LENG8/PAXT.

We thank the referee for allowing us to expand on the topic of LENG8–PS specificity. As the referee points out, multiple DExD ATPases exist in human cells and share significant amino-acid similarity. However, amongst all the DExD-box ATPases only UAP56 (DDX39A/B) is predicted confidently to interact with LENG8 or a LENG8–PS complex (Schmid EW, Walter JC, *Mol Cell*, 2025). Structural super-positions with our LENG8–PS–UAP56 structure revealed that likely all other DExD-box ATPases, except UAP56, would clash with the PCID2–SEM1 (PS) module of the LENG8–PS complex, making complex formation between these other ATPases and LENG8–PS unlikely (new Supplementary Fig. 3e). In addition, the UAP56 NTD is required for high-affinity binding to LENG8–PS complexes and their paralogs. This UAP56 NTD is highly conserved among eukaryotes, yet found only in orthologs of UAP56 and no other DExD-box ATPase. To further support this, we had performed *in vitro* ATPase assays in the original manuscript, showing that LENG8–PS indeed acts only on UAP56, but not on EIF4A3, an evolutionarily closely related DExD-box ATPase, which however serves a different function in mRNA biogenesis (original Supplementary Fig. 7f,g). In addition, we now show, through *in vitro* pulldowns, that LENG8–PS binds only UAP56 and not EIF4A3 (new Supplementary Fig. 3f). These data suggest that LENG8–PS targets only UAP56 and not other ATPases. So why are multiple ATPases, including EIF4A3, lost upon the IP of the LENG8-binding deficient ZFC3H1 $^{\Delta730–747}$ mutant? We propose here that UAP56 is the main connector between pA^{+} RNPs and LENG8–PS–ZFC3H1. Thus, when ZFC3H1 cannot bind LENG8 anymore the connection to RNPs is (by large) lost, and thus all RNP components are depleted. This includes the EJC with EIF4A3 as well as other potentially RNA-engaged ATPases (new Supplementary table 2).

Finally, our new orthogonal iCLIP data, showing UAP56 and LENG8 enrichment on pA^{+} PPT transcripts, mono-exonic ncRNAs and PROMPTs (new Supplementary Fig. 11d-h) argues against EIF4A3-mediated LENG8-PS recruitment, while supporting the direct involvement of UAP56.

2) The authors show in their cryoEM structures that UAP56 binds LENG8-PSM and SAC3D1-PSM in a closed RNA-bound conformation. This is in contrast to the cryoEM structure of TREX-2M, where UAP56 is not clamped onto RNA. First, how could the authors capture a UAP56–TREX-2M complex in which UAP56 was released from RNA if the complex was formed in the presence of the non-hydrolysable ATP analog AMP-PNP? Second, what is the biological relevance of the distinct RNA-binding states of UAP56 in TREX-2 and TREX-2-like modules? Third, the methods section, but not the

main text or figures, mentions ‘UCM1’ and ‘N-UBM’ in the preparation of cryoEM samples. Please define these terms and their significance.

We thank the reviewer for these comments, which we have addressed below.

First, we note that UAP56 reaches a stable RNA-clamped conformation after the hydrolysis of ATP. This means that the RNA-clamped complex consists of UAP56–ADP–Pi–RNA, and that the rate limiting step in UAP56’s ATPase cycle is not hydrolysis but rather product release (see also Hohmann et al., *Nature*, 2025). TREX2, as well as SAC3D1–PS and LENG8–PS, do not act on UAP56 by impacting ATP hydrolysis, but rather by facilitating the release of the reaction products ADP and Pi. When we attempted to analyze the cryo-EM complexes of UAP56 and TREX-2M or LENG8–PS in the presence of ATP (which would have been hydrolyzed to ADP+Pi in clamped UAP56), we found no evidence of any engagement of the RecA lobes of UAP56 with TREX-2 or LENG8–PS, suggesting that in this sample UAP56 had been unclamped and ADP removed from UAP56, effectively completing the cycle. Only in the presence of the non-hydrolysable analogue AMP-PNP did we found engagement of the RecA lobes (post release for TREX-2, pre-release for LENG8–PS and SAC3D1–PS), presumably because AMP-PNP is removed less efficiently from the nucleotide binding site as compared to ADP.

Second, the fact that we found by cryo-EM, under otherwise identical conditions, pre-release complexes for SAC3D1–PS–UAP56 and LENG8–PS–UAP56 and post-release complexes for TREX-2M–UAP56 was first and foremost a lovely technical curiosity, providing us with novel insights as we could now visualize and analyze these different states. Comparison of the structures suggested that the association of the UAP56 RecA2 lobe might be slightly less favorable in case of TREX-2, potentially shifting the equilibrium in our cryo-EM sample towards the post-release state. However, biochemically we did observe that the three complexes behave similarly with UAP56 (strong stimulation of the apparent ATPase rate as observed in ATPase assays (Supplementary Fig. 2h-i) and robust unclamping of UAP56 from RNA (Fig. 1c,d), suggesting that the observation of different states by cryo-EM might be interesting for the analysis of the biological mechanism but not beyond.

Third, to prepare complex samples for cryo-EM we used fusion proteins where UAP56 was fused to two small UAP56-binding peptides: the ‘UAP56 Clamping Motif’, UCM1, and the N’-terminal type of the UAP56 Binding Motif’, N-UBM, which we reasoned would further promote the RNA-clamped conformation of UAP56 and more accurately mimic UAP56’s native mRNP-bound state, where N-UBM and UCM are present at high local concentrations to engage RNA-bound UAP56 (Hohmann et al., *Nature*, 2025). However, as we did not observe these peptides in our cryo-EM structures, despite being compatible with the resulting LENG8–PS–UAP56 structure, they bear no influence on our conclusions. We thank the referee for pointing out this oversight and have now adjusted the Methods section to be clearer on the sample (revised manuscript lines 799-807).

3) Fig. S1d,e show that much less UAP56 is engaged with than without LENG8-PSM or SAC3D1-PSM. Likewise, Fig. S7g shows that much less UAP56 (or EIF4A3) is engaged with LENG8(491-800) than with LENG8-PSM. How could the authors assume that lower UAP56/EIF4A3 concentrations would not influence the number of ATP molecules

We apologize for the misunderstanding. The figure panels mentioned by the referee do not show pulldown experiments to test protein-protein interactions or engagement. They instead show the composition of the samples used for the ATPase assays in former Fig. 1c,d and Supplementary Fig. 7f. In these ATPase assays we needed to adjust experimental conditions so that the observed ATPase rate was within certain parameters: with too slow ATP turnover we could not determine the ATPase rate

reliable against background, with too high ATP turnover the ATP in the sample would be exhausted before reliable measurements could be done. UAP56 can form stable, RNA-clamped complexes in the presence of ATP with a half-life of over 30 min at 37°C. Thus, the observed ATPase rate of isolated UAP56 with RNA was very slow and we needed to conduct the experiment with a high UAP56 concentration of 2 µM. LENG8–PS and SAC3D1–PS led to a substantial increase in the observed ATPase rate, and we conducted the experiment with the much lower concentration of only 0.1 µM. The same reasoning held for EIF4A3, hence on the gel depicting the ATPase assay sample (old Supplementary Fig. 7g), UAP56 / EIF4A3 appeared much more prominent in isolation compared to the samples with LENG8–PS or SAC3D1–PS. In the analysis of the ATPase assays the ATPase rate was normalized for the input protein concentration, making measured rates across different conditions comparable despite the different ATPase concentrations in the samples. We add that we have now also: i) improved the ATPase assay sample SDS-PAGE gels and their labeling for better clarity, and now show assay results and sample gels always as part of one figure panel side-by-side; ii) included a pulldown experiment to show that UAP56, but not EIF4A3, binds LENG8–PS *in vitro* (Supplementary Fig. 3f).

4) Our interpretation of Fig. S6c is that only RNAs in Cluster 4 have increased C/N ratios after rapid ZFC3H1 depletion or, to a lesser extent, after rapid LENG8 depletion. In other words, ZFC3H1 and LENG8 retain only a very small fraction of PAXT decay targets in the nucleus. The authors should clarify why they focus on Cluster 1 and do not discuss, in particular, Cluster 4.

The clustergrams of log2 fold changes of pA^+ RNA coverage in former Supplementary Fig. 6c (now Supplementary Fig. 10c) were shown to achieve two main purposes: i) reveal the transcriptome-wide effects of ZFC3H1- and LENG8-depletion and ii) report in which cell compartment PAXT-sensitive RNAs accumulate. The reason that we start out in the paper by discussing Cluster 1 is that we want to first emphasize the large portion of pA^+ RNAs, that is inert to PAXT-mediated decay. Apart from the upper part of the Cluster 1 heatmap, where somewhat sensitive and low exon count transcripts reside, this cluster is dominated by multiexonic mRNAs, which are largely resistant to ZFC3H1 and LENG8 depletions. This reflects a central part of our model, that longer and exon-dense RNAs are capable of overriding nucleoplasmic decay to reach TREX2 at the nuclear membrane for subsequent export. Again, we believe Cluster 1 illustrates this point really well. To explain the behavior of PAXT-sensitive transcripts (Clusters 2–4), we opted to not simply rely on C/N ratios. Instead, we focused on significant coverage changes providing information on both RNA decay as well as in which compartment upregulated RNAs would accumulate. Here, most PAXT substrates accumulated in both the nucleus and the cytoplasmic (Cluster 2, n=2751), which reiterates the roles of LENG8-PS and ZFC3H1 in both nuclear decay and retention of primarily short and low exon content RNA species. Another fraction of PAXT substrates (Cluster 3, n=1484) showed significant upregulation only in the nucleus, while the fraction noted by the referee (Cluster 4, n=615) accumulated mostly in the cytoplasm. Now it is important to note, that both RNA export capacity and cytoplasmic half-life will dictate the C/N ratio of a transcript. Therefore, we can only reliably suggest a role for PAXT in the turnover of these substrates, while elevated cytoplasmic signal might partly be due to a lower cytoplasmic decay rate. Even so, the appearance of a transcript in the cytoplasm upon ZFC3H1/LENG8 depletion will mean that these factors retained the RNA in the first place. With these caveats in mind, it indeed appears that Cluster 4 RNAs extensively leak to the cytoplasm upon ZFC3H1 or LENG8 depletions. We interpret this to mean that these RNAs differ from those in *e.g.* Cluster 2 by being subjected to faster export when they are no longer retained and degraded in the nucleus. Cluster 2 RNAs are also retained, however, here retention is followed by efficient nuclear decay. These matters are discussed in the manuscript (lines 266-269).

5) What data support the statement in the Discussion that “TREX-2 seemingly grants export to any pA+ RNA provided that this pA+ RNP can make its way to the NPC”? Indeed, the main text reports that “[rapid reduction of GANP protein] resulted in the downregulation of short pA+ RNAs with few introns”, and only this set of RNAs seems to have been included in the “downregulated” genes shown and analyzed in Fig. 4b. Additionally, what data support the statement that “Both TREX-2 and PAXT engage UAP56-bound pA+ RNPs indiscriminately”? The authors argue at length that PAXT targets specific RNA species, making the “indiscriminately” comment contradictory.

We thank the referee for reflecting on these wordings, which agreeably were not sufficiently clear. Concerning the statement ‘TREX-2 seemingly grants export to any pA^+ RNA provided that this pA^+ RNP can make its way to the NPC’, our intention was to convey that pA^+ transcripts, that are normally retained and degraded in the nucleus, can access the cytoplasm via TREX-2–mediated export, in conditions where their nuclear turnover is prevented. This suggests that LENG8-PS and GANP-PS have the capacities to target similar pA^+ RNPs. We have now revised the Discussion text accordingly to illustrate this interpretation (revised manuscript lines 368-371). The mentioned data from Fig. 4b indeed inspired the statement; primarily short pA^+ RNAs displayed significant downregulation in this total pA^+ RNAseq dataset and the conducted rapid GANP depletion did not cause a widespread change in pA^+ RNA abundance. This may reflect i) the slower kinetics in accumulating long, fully processed transcripts, that were predominantly cytoplasmic prior to GANP depletion and/or ii) that only a subset (short) of pA^+ RNAs are affected by GANP depletion. In any case, we hope the revised wording now reflect the obtained data better.

Concerning our use of the term ‘indiscriminately’, we intended to indicate that both TREX-2 and PAXT can engage UAP56-bound pA^+ RNPs without clear sequence specificity at the level of UAP56 binding. This was not meant to suggest identical functional outcomes. Our data imply that while both pathways can engage similar RNPs, efficient decay is preferentially achieved for shorter transcripts. Because we cannot formally determine whether subsets of RNPs are preferentially targeted by one pathway over the other, we have revised the sentence in the Discussion to keep this question open (revised manuscript lines 372-373, 377-378).

6) The authors did not acknowledge the existence of a UAP56–LENG8-PSM cryoEM structure (Clarke et al., Nat Comm. 2025, where it is referred to as TREX-2.1/DDX39B) and a preprint investigating the role of LENG8 in nuclear RNA retention and PAXT-mediated RNA decay (Tian et al., bioRxiv. 2025, where LENG8-PS is referred to as the Repressor of EXport complex, or REX). Both papers were very recently published, so the authors possibly missed them during the submission of this manuscript.

We thank the referee for pointing out these recent studies, which became available after the submission of our current manuscript. These two studies independently support aspects of our findings, albeit from different perspectives. The first study from the Yi Ren laboratory describes a “TREX-2.1”/DDX39B (UAP56) or LENG8–PCID2-SEM1–UAP56 complex (Clarke et al., *Nat. Commun.*, 2025). Their cryo-EM structure captured UAP56 in a post-release state, similar to what we had previously shown for TREX-2–UAP56 (Hohmann et al., *Nature* 2025). In this study, we instead provide structures of the novel pre-release complexes, where UAP56 is found in its RNA-clamped form. The structural data from the Ren lab is thus complementary to our previous data and allow us in this study to report the missing intermediate in the UAP56–LENG8–PS cycle through cryo-EM. The second study from the Yongsheng Shi laboratory, currently available as a preprint and released during the revision process, identifies LENG8 as a nuclear RNA retention factor through a CRISPR-based screen (Tian *et al.*, *bioRxiv*, 2025).

The paper further links LENG8 to the PAXT pathway, referring to the LENG8–PS module as the 'Repressor of Export (REX)' complex. This paper focuses on nuclear RNA retention, suggesting that this is mediated through an interaction of LENG8 with splicing factors, particularly U1 snRNP components. This connection is indeed interesting and may help explain how PAXT targets with retained introns are prevented from accessing the export machinery, complementing our findings.

As these two papers became available during our manuscript submission, we have now included them in the relevant Results sections and commented on how these findings complement our own (revised manuscript 115-116, 255-256). At the same time, we refrain from introducing a new nomenclature for the different TREX-2-like complexes at this time. We anticipate that additional naming will run the risk to add conceptual ambiguity before the full functions of all three complexes are known. In particular, we consider the 'REX' designation of LENG8–PS to be premature as it: i) does not capture its central role in PAXT-mediated RNA decay and ii) might hinder future unbiased investigation of additional LENG8–PS functions, as we discuss in our manuscript:

> *"... targeting of LENG8-PS to decay-insensitive transcripts might nevertheless play a role in maintaining sufficient levels of free nuclear UAP56, without which mRNA biogenesis defects, R-loop formation and genomic instability would prevail. The importance of such UAP56 recycling might in fact explain why budding yeast, where the PAXT complex has been lost, still harbors the LENG8-PS homolog."*

Minor comments:
1) The Jensen lab recently demonstrated that PAXT-mediated decay of short RNA targets requires the binding of ZFC3H1 to cap-associated protein ARS2 (Polák et al., Cell Reports. 2023). It would be interesting to discuss whether ARS2-mediated and LENG8-mediated recruitment of PAXT typify different RNAs—for example, based on "how short" they are.

As also mentioned above (referee 1, point 2), we previously reported that the ARS2 protein can recruit ZFC3H1 through an interaction of the ARS2 effector domain with the so-called ARS2 Recruitment Motif (ARM) of the ZFC3H1 N-terminus and that this interaction can be relevant for targeting select PAXT substrates (Polak et al., *Cell Rep.*, 2023). We now explored if ARS2-targeting of early transcripts to ZFC3H1 may enhance the activity of PAXT, perhaps affecting RNPs where UAP56 is not yet engaged. As discussed above, this might help explain the difference in effect sizes of the LENG8 and ZFC3H1 depletions. Hence, we compared ARS2- and LENG8-dependent PAXT targets by intersecting RNAseq data from a ZFC3H1 complementation assay using the ZFC3H1 ARM mutant, that cannot bind ARS2 (Polak et al., *Cell Rep*., 2023), with the RNAseq data from the present paper obtained using the LENG8 binding-deficient ZFC3H1 Δ730-747 mutant (Supplemental Fig. 9i). This revealed a significant overlap between LENG8- and ARS2-dependent ZFC3H1 substrates (new Supplementary Fig. 9k), consistent with the notion that LENG8 and ARS2 can act on the same pool of ZFC3H1-regulated RNAs. We therefore suggest that, at least part of, the effect size difference between LENG8- and ZFC3H1-depletion phenotypes is explained by the ability of ARS2 to 'enhance' the PAXT pathway (revised manuscript lines 239-246).

2) As a general comment, the structures might be better annotated. For example:
o Residues mentioned to describe the SAC3D1–UAP56 interaction should be shown in Fig. 1h. Minimally, SAC3D(P111) (and the corresponding residue in GANP), UAP56(R135), and UAP56(K334) should be added. Otherwise, it is difficult to understand/visualize how these proteins bind.

We thank the referee for raising this concern. When preparing figures to illustrate structural data we tried to focus panels on one key finding to avoid overloading the figures. We have now also included and labelled additional residues mentioned in the Results section. In Fig. 1g we want to specifically focus on the residues interacting with the nucleotide. We thus now in addition include a panel showing the positioning and interactions of the SAC3D1 wedge loop (Supplementary Fig. 3d).

o Where are the 'wedge loops' in the AlphaFold predictions (Fig S1a) or the cryoEM structure (Fig. 1g).

The structures in original Supplementary Fig. 1a depicted the apo complexes of TREX2^{M}, SAC3D1–PS or LENG8–PS. In these structures the wedge loop is flexible and thus cannot be observed by cryo-EM. We hence depicted the wedge loop with a dotted line which is now also labelled 'wedge loop' (Supplementary Fig. 3a). In Fig. 1g the wedge loop was hidden behind UAP56 and can only be visualized once the structure is rotated by -90° around the y-axis and obstructing features are stripped away. This is the view shown in Fig. 1g and Supplementary Fig. 3d.

o Where in Fig. S2e are the regions selected in S2g.

The goal of original Supplementary Fig. 2g (now Supplementary Fig. 5g) was to provide a sample of the cryo-EM data quality. These sample regions are usually taken from rather interior sections of the respective proteins, as they tend to have slightly higher resolution. Consequently, it is impossible to reasonably illustrate in these panels where these sections were taken from, and we thus did not provide a visual guide for this.

o Fig. S4d. It would be helpful to annotate where the ZFC3H1 amino acids 730-747 are localized in the predicted alignment error maps. We imagine that they align with the blue region in the A/B intersecting areas.

We thank the referee for this suggestion and have now added additional annotation in the PAE plot outlining the interacting regions in LENG8 and ZFC3H1 (new Supplementary Fig. 8e).

3) Unlike what is stated in the text, based on results shown in Figs. S3b and S3c, the co-IP of RNA exosome constituents with LENG8 or with PCID2, but not with ZFC3H1, is partially sensitive to RNase digestion (although sometimes below a selected P-value of 0.01). Could this reflect the simultaneous association of PAXT-bound LENG8-PS complexes with multiple UAP56 molecules (or other helicases?) separated by RNA stretches on given transcripts, only one/some of which engage the RNA exosome?

This is an interesting suggestion. We indeed consider that LENG8-PS and by extension ZFC3H1-MTR4-exosome might engage UAP56 as part of an RNP where additional UAP56 molecules are bound throughout the transcript (also mentioned above in our response to point 1c). A good indication for this exists via the RNase sensitivity of UAP56, and other RNP components, in both the LENG8 and ZFC3H1 IPs. The RNase-dependent changes observed for exosome components are only modest in magnitude and do not reach statistical significance. We therefore decided to interpret these with caution.

4) Page 5 lines 25-26. We believe that the authors mean that the alpha-helices are in a region N-terminal "to" (and not "of") the SAC3 domain.

Correct, we have now adjusted the text.

5) Efficient acute depletion of LENG8 was not demonstrated in Fig. S6a, but it should be.

We thank the referee for pointing this out. In the new Supplementary Fig. 10a, we have now included anti–HA-tag western blotting analysis to demonstrate efficient depletion of LENG8–2xHA–dTAG in the endogenously tagged HeLa cells.

6) In the Discussion, the authors propose that PAXT may release UAP56 from longer RNAs, thereby increasing their nuclear export via TREX-2. How could this be possible if TREX-2 requires UAP56 for docking?

We apologize for the apparent ambiguity of our phrasing. In fact, we propose, as the referee correctly states, that export relies on the presence of UAP56 on the RNA. We assume the referee is referring to this section in the Discussion:

> '*Both TREX-2 and PAXT may engage UAP56-bound pA$^+$ RNPs. Owing to the nucleoplasmic adaptor of the 3'-5' exonucleolytic exosome, PAXT targeting only translates into efficient decay*
> '

We agree that the phrasing, particularly of the sentence highlighted in bold face, was not ideal. The point we tried to make was: long (m)RNAs will harbor multiple UAP56 molecules. Therefore, an encounter with PAXT could still remove a fraction of UAP56 molecules from the RNA; however, for longer RNAs the PAXT encounter would be less likely to be in the vicinity of the RNA 3′ end, which engages the exosome. Thus, the RNA would not be degraded and the remaining UAP56 molecules can promote export via TREX-2. We have reworked the wording of this section to avoid the perception that a PAXT encounter directly promotes export (revised manuscript lines 376-378).

7) Could the authors speculate on the molecular function of the presumably cytoplasmic UAP56–SAC3D1–PS complex?

Thank you for this interesting question. IP-MS experiments and Alphafold screens have not revealed any prominent putative interactors for SAC3D1, unlike for LENG8 or GANP. In line with this, and again unlike LENG8 or GANP, SAC3D1 has no additional elements besides the SAC3 domain required for complex formation with PCID2–SEM1. During mRNA export UAP56 engages TREX-2 at the nuclear pore, and this in turn releases UAP56 from the mRNA to diffuse back into nucleoplasm. However, there is no mechanism to ensure that all UAP56 molecules are removed from the mRNA in the nucleus prior to export by TREX-2 (or LENG8–PS). Together, these observations could suggest that the function of SAC3D1–PS might be the removal of residual UAP56 molecules from the cytoplasmic mRNA in the cytoplasm, yet future work will be needed to investigate this hypothesis. We have now included a sentence on this speculated activity in the revised Discussion (revised manuscript lines 384-386).

8) The supplementary tables require a description of each column. In their current state, it is often hard and sometimes impossible to figure out what each column corresponds to.

We thank the referee for pointing out this issue. In the revised Supplementary tables, where applicable, column descriptions are now provided in the first sheet of each .xls file.

9) Figure legends should specify the number of biological replicates used in each experiment.

We have now provided the number of biological replicates in the figure legends where this information was previously missing.

Referees' comments:

Referee #1 (Remarks to the Author):

The authors have satisfactorily addressed the comments raised in the initial review. They have clarified several points in the text and included a number of additional experiments that further support their conclusions. Overall, these revisions have strengthened the manuscript and improved its clarity.

We thank the Referee for the constructive feedback, clearly improving the quality of the manuscript.

Referee #2 (Remarks to the Author):

I thank the authors for their thorough and constructive engagement with all of my comments. The revised manuscript is substantially strengthened by the inclusion of new cryo-EM structures, improved complementation assays at near-endogenous expression levels, and replication of key findings in HCT116 cells. The study now provides a compelling structural and functional framework for nuclear pA+ RNA fate determination centered on UAP56-bound RNPs. I discuss each of my original points below.

1) Structural evidence for UAP56:LENG8:PSM

The authors have gone well beyond what I envisaged. Four (!) new cryo-EM structures-apo LENG8-PS (3.5 A), apo SAC3D1-PS (3.6 A), LENG8-PS bound to the UAP56 NTD (4.9 A), and LENG8-PS bound to RNA-clamped UAP56 (6-12 A) now replce the AlphaFold predictions that were my primary concern. The experimental density for the clamped state, although at lower nominal resolution, unambiguously establishes that LENG8-PS engages RNA-clamped UAP56 in the same manner as SAC3D1-PS at 2.6A. Together with the new biochemical validation of the UAP56-LENG8-PS interface, this fully resolves my concern about over-reliance on computational predictions.

2) Endogenous-level complementation assays

I appreciate the authors' efforts to titrate doxcycline induction to achieve near-endogenous expression of all LENG8-3xFLAG variants. The strategy using the LENG8-3xFLAG line as a reference for endogenous levels is a reasonable alternative to CRISPR knock-in, and the new western blot data in supplementary section demonstrating comparable expression levels across constructs is reassuring. The recapitulation of the original rescue phenotypes under these controlled conditions with wild-type LENG8 rescuing, and F301A, R563A, and TRR mutants failing to rescue strengthens the mechanistic conclusions. I accept the authors' rationale that RT-qPCR across seven diverse PAXT targets is sufficient for this assay without full RNA-seq.

3) Replication in a second cell line

The generation of 2xHA-dTAG LENG8, GANP, and ZFC3H1 lines in HCT116 cells, together with IP-MS and fractionated pA+ RNA-seq, represents a significant addition of experimental data. The agreement between HeLa and HCT116 in protein-protein interaction profiles and in the nuclear/cytoplasmic redistribution of mono-exonic and low-exon-content RNAs upon LENG8, ZFC3H1, or GANP depletion effectively nullifies concerns about HeLa-specific artefacts. I consider this point fully and convincingly addressed.

4) LENG8/GANP double-depletion epistasis

I acknowledge the considerable effort the authors invested in attempting these experiments across multiple cell-line configurations. The observation that dual LENG8/GANP depletion (in HCT116) and dual ZFC3H1/GANP depletion (in HeLa) both result in severe growth impairment and rapid transcriptional shutdown is itself informative. The authors' interpretation that simultaneous loss of both SAC3-domain modules prevents UAP56 recycling, thereby collapsing the RNP biogenesis cycle is consistent with the competition model and with the published phenotype of the FLAG-UAP56 M1 mutant that cannot engage either module.

Although the intended epistasis readout could not be obtained, I would encourage the authors to incorporate the supporting data from the rebuttal (the RT-qPCR of nascent transcripts and the intron-count analysis from the ZFC3H1/GANP double depletion) into the supplementary material. The transcriptional shutdown phenotype upon dual depletion constitutes positive evidence for the UAP56-recycling model and, in my view, would meaningfully strengthen the paper's central claim. I leave this as a strong recommendation, not a requirement for acceptance. Maybe the authors are saving this for a follow-up, but I think it is really helpful to include here.

5) PABPN1 and poly(A) tail protection

The authors have performed the requested PABPN1/LENG8 co-depletion experiment and obtained a clear result showing co-depletion does not increase PAXT substrate accumulation beyond the individual depletions, which suggests that PABPN1 and LENG8 operate sequentially rather than in parallel. The proposed model in which PABPN1 acts downstream of LENG8-PS-mediated UAP56 release to facilitate exosome engagement with the RNA 3'end is logical and sits well with published findings from the Jensen laboratory and others.

I understand the authors' reluctance to open a full investigation of nuclear PABPs within this manuscript. However, I would strongly suggest (again) that the PABPN1/LENG8 co-depletion data be included in the supplementary material, accompanied by a few sentences contextualizing the sequential-action model. This finding provides a valuable mechanistic boundary condition that readers of the paper would benefit from, and its omission would leave the PABPN1 connection unnecessarily opaque given that the experiment has already been performed and the result is clean. These reports will be published together with the manuscript so why not include?

Pending these minor revisions, I am supportive of publication in Nature.

We thank the Referee for the constructive and insightful comments, and for acknowledging our efforts during this fruitful revision. Given the choice, we would respectfully prefer not to incorporate the obtained results from points 4) and 5) above. Although consistent with the proposed models, our interpretations of the results from both 4) and 5) remain, in our opinion, too speculative to include without further experimental support. As standalone biological insights, we do not find it sufficiently robust at this stage but would like to develop both lines of research further.

Referee #3 (Remarks to the Author):

Nature manuscript 2025-07-17305A

"Molecular basis of polyadenylated RNA fate determination in the nucleus".

The authors have performed additional work to address concerns raised by each reviewer. We believe that our comments were satisfactorily addressed in general. Nonetheless, we still have a few comments and a recommendation for improvement.

We are grateful for their positive evaluation of our revisions. Below we provide a point-by-point response to the remaining criticism.

Major comments:

- We previously asked why release of UAP56 by LENG8-PS would be required for exosome-mediated decay. The authors responded as follows: “However, we would like to clarify that we do not propose that release of UAP56 from RNA is directly required for exosome-mediated RNA decay. Instead, we suggest that UAP56 release is necessary to prevent the RNA from engaging TREX-2 and being exported. In this model, UAP56 removal indirectly favors nuclear RNA retention and decay by blocking productive recruitment of the export machinery, rather than by actively promoting exosome function.” We are, however, unconvinced by this response. What would be the purpose of LENG8-PS simultaneously releasing UAP56 from RNA and recruiting the exosome via PAXT if both actions were not functionally connected? Are the authors suggesting that LENG8-PS sensitizes RNAs to decay in two independent mechanisms: possibly, (1) via the release of UAP56 to block nuclear RNA export, and (2) via the recruitment of PAXT on RNA, which would only directly lead to decay if PAXT were recruited close to the RNA 3’-end?

We thank the Referees for this central question and for allowing us to clarify our model.

As summarized by the Referees in their final sentence, we do indeed suggest that LENG8-PS may impact UAP56-bound RNP in these two ways. UAP56 acts as an export promoting mark as it allows the RNA to reach the NPC via TREX-2. Thus, the mere removal of UAP56 will counteract export and favor decay. For longer RNAs the removal of a fraction of UAP56 might be without consequence as long as the RNA is not turned over and as long as some UAP56 remains to allow TREX-2 binding. Such UAP56 removal might even be beneficial to guarantee a sufficient pool of free UAP56. From these interactions engagement of LENG8-PS with RNA

close to its polyadenylated 3'end will likely lead to RNA degradation through PAXT-exosome. This sensitizes shorter pA^+ RNAs (like PROMPTs and PTTs) hosting fewer UAP56 molecules.

To provide clarification, we have now altered the Discussion section, lines 380-382:

'Owing to the nucleoplasmic localization of newly made pA+ RNPs, they would first encounter PAXT. However, being an adaptor of the 3'-5' exonucleolytic exosome, PAXT targeting only translates into efficient decay if it occurs in the vicinity of the RNA 3'end. This condition greatly sensitizes short transcripts. In contrast, for longer RNAs, which are compacted into larger RNPs with multiple UAP56s, a PAXT encounter may merely result in the release of an UAP56 molecule. This, on itself, would counteract export indirectly, however, as long as a sufficient number of UAP56 molecules remains on the transcript, nuclear export via TREX-2 is still possible.'

- The authors write on line 104 that the new « TRR » mutation targets the UAP56-binding interface of LENG8. We could not find any supporting information in the manuscript. The authors' response to our comment #1 indicates that they use a "structure-guided approach" to identify which residues to mutate. This needs to be clarified and properly illustrated in the manuscript.

We thank the Referees for pointing this out. The TRR mutant was designed (1) to target the LENG8–UAP56 NTD interaction and (2) to lead to steric hinderance between LENG8 and the RecA2 domain of UAP56, thus effectively preventing the interaction of LENG8–PS and UAP56.

We are now:

- more specific in the manuscript (main text lines 105-107):

'Of note, mutating three residues targeting the UAP56-NTD and UAP56-RecA2-binding interfaces of LENG8 ($LENG8^{TRR}$) led to diminished LENG8–PSM–UAP56 interaction (Extended Data Fig. 1j,k. Supplementary Fig. 2a)…'

- providing a figure showing the targeted residues (new Extended Data Fig. 1j)
- including a more detailed description in the legend for Extended Data Fig. 1j.

Minor comments:

- We find it confusing when the authors state in the Abstract that LENG8-PS and GANP-PS are "functionally" equivalent. We suggest they change "functionally" by "biochemically" (as used on line 152). Alternatively, the authors could clarify that what they refer to is the shared ability of LENG8-PS and GANP-PS to release UAP56 from RNA (as stated on lines 62-63). Otherwise, LENG8-PS and GANP-PS fundamentally differ in their ability to mediate nuclear RNA decay or export, respectively.

We have changed “functionally” to “biochemically” as suggested.

- As suggested by the authors in their response to our comment #1, it would be helpful to the reader if they included an explanation for how ATP hydrolysis in UAP56 ATPase activity assays relates to UAP56 rebinding to RNA after release.

We agree that a more detailed explanation of the effects observed in the ATPase assay would be beneficial. We now include a minimal description in the manuscript main text (lines 91-93):

“TREX-2^{M} facilitates the release of ADP and Pi from UAP56, thus accelerating the rate-limiting step in the disassembly of UAP56–RNA complexes, releasing free UAP56 available for RNA re-binding, and resulting in an increased apparent ATPase activity[7].”

We further include a detailed description, as provided in our first-round comment #1 response, in the figure legend for the first ATPase assay, Extended Data Fig. 1c,d.

- The statement that “only ZFC3H1^{WT} efficiently suppressed the accumulation of PAXT-substrates” (Lines 236) does not seem correct. Fig. S9i shows that ZFC3H1$^{\Delta 730\text{-}747}$, which fails to bind LENG8, retains about half the activity of ZFC3H1WT. It most likely reflects ARS2-dependent LENG8-independent activity of ZFC3H1, as discussed on lines 239-246. The authors concluded that LENG8 and ARS2 have redundant actions on ZFC3H1-mediated decay of the same pool of RNAs. We would argue that the actions are only “partially” redundant since mutating the sites of ZFC3H1 that bind LENG8 or mutating those that bind ARS2 is sufficient to partially stabilize PAXT targets.

Thanks for this comment. We now clarified this in the text to ensure against misinterpretation.

Main text lines 239-240: “only ZFC3H1^{WT} efficiently suppressed the accumulation of PAXT-substrates, whereas the ZFC3H1$^{\Delta 730\text{-}747}$ construct only retained partial activity “

Main text line 249: We now have used “…partially redundant LENG8 and ARS2 actions…” in line 250 of the main revised manuscript text.

- The authors should clarify in the main text how they define “PAXT substrates” (or “PAXT-sensitive RNAs”) when this concept is first introduced. We believe this occurs on line 236, which refers to Fig. S9i, which in turn refers to the Methods section. The methods state: “Due to the strong correlation between coverage changes of LENG8- and ZFC3H1-depletions (Fig. 3c), ‘PAXT sensitive’ TUs were defined as upregulated in either of the two depletions.” Additionally, the authors should make it clear if they included all the pA+ PPT transcripts defined based on ZFC3H1 depletion only (see lines 226-231).

We thank the Referees for pointing out this issue, which we have now clarified in both the main text and the Methods section. Indeed, the reference in Extended Data Fig. 8i (previously Fig. S9i) refers to “ZFC3H1 substrates” only. In the manuscript, our first use of “PAXT substrates”

refers to previously established ZFC3H1-sensitive RNAs. We have now added “formerly established” upon the first text appearance and referenced to published studies. The later term “PAXT-sensitive RNAs” is referring to RNAs upregulated by either ZFC3H1- or LENG8-depletion as stated in the Methods and also now in the main text lines 263-265.

To annotate pA^+ PTTs several stages of filtering were applied, including the initial identification of TUs harboring increased promoter-proximal RNA signal upon either LENG8- or ZFC3H1-depletion as well as their intersection with pA^+ peaks from published 3’end-seq data obtained after ZFC3H1-depletion. We have now clarified this briefly given main text constraints (lines 229-232):

“To incorporate these in our analysis, we identified TUs displaying such increased pA+ RNA coverage upon ZFC3H1 or LENG8-depletion (Extended Data Fig. 8e) and intersected data with pA+ RNA 3’end peaks previously identified upon depletion of ZFC3H1 (ref.[28], see Methods).”

- The number of biological replicates is still missing from most figure legends (for example Figs 1c, 1d, 2a, 2f, 2g, 3c, 3d, 3e, 3f, 4b, 4c, 4d). This information should be included.

We now state the number of biological replicates when it was used for quantification and statistical testing. For experiments using SDS-PAGE gels as readouts, we now state in the Method’s Statistics and Reproducibility section that every such experiment was reproduced at least three times.

Referee #4 (Remarks to the Author):

I co-reviewed this manuscript with one of the reviewers who provided the listed reports.
